# Spurious alignment between large language models and brains can emerge from non-robust methods and overlooked confounds

**DOI:** 10.1038/s41467-026-72253-7

**Published:** 2026-04-27

**Authors:** Nima Hadidi, Ebrahim Feghhi, Bryan H. Song, Idan A. Blank, Jonathan C. Kao

**Affiliations:** 1 Department of Electrical and Computer Engineering, University of California, Los Angeles, Los Angeles, CA USA; 2 Neuroscience Interdepartmental Program, University of California, Los Angeles, Los Angeles, CA USA; 3 Department of Computer Science, University of California, Los Angeles, Los Angeles, CA USA; 4 Department of Linguistics, University of California, Los Angeles, Los Angeles, CA USA; 5 Department of Psychology, University of California, Los Angeles, Los Angeles, CA USA; 6 Department of Neurobiology, University of California, Los Angeles, Los Angeles, CA USA

**Keywords:** Neural encoding, Language

## Abstract

Emerging research seeks to draw neuroscientific insights from the neural predictivity of large language models (LLMs). However, as results rapidly proliferate, there is a growing need for large-scale assessments of their robustness. Here, we analyze a wide range of models and methodological approaches across three widely used neural datasets. We find that the use of shuffled train-test splits has contributed to findings that are influential but spurious. Furthermore, how activations are extracted from LLMs can bias results in favor of specific model classes. Lastly, we find that confounding variables, particularly positional signals and word rate, perform competitively with trained LLMs and fully account for the neural predictivity of untrained LLMs on these neural datasets. Although many studies in the field avoid these pitfalls, our results indicate that some apparent alignment between LLMs and brains has emerged from non-robust methods and overlooked confounds.

## Abstract (web summary)

Whether large language models (LLMs) process language in a human-like manner remains unclear. Here, the authors re-examine prior LLM-to-brain mapping results, demonstrating that LLM-brain alignment can result from non-robust methods.

## Introduction

Do large language models (LLMs) process language like humans? One way to investigate this question is through a neural encoding framework, in which an LLM’s internal representations of linguistic stimuli are used to predict brain responses to the same stimuli. Across multiple studies, such predictions have been highly effective^1–5^. An emerging research program now seeks to understand what properties of LLMs drive this neural predictivity.

In the most cited of these studies, Schrimpf et al.^2^ evaluated the neural predictivity of 43 models—including transformer-based LLMs, smaller recurrent neural network (RNN) models, and static (i.e., non-contextual) word embeddings—across three neural datasets and reported three main results. First, LLMs trained to predict the next word, specifically *GPT2XL*^6^, provided the best neural predictivity across all 43 models, accounting for 100% of the explainable neural variance in one dataset. Second, the neural predictivity of models was positively correlated with one model property in particular, their next-word prediction ability. These two findings were interpreted as “computationally explicit evidence that predictive processing fundamentally shapes the language comprehension mechanisms in the human brain"^2^. Third, untrained (i.e., randomly initialized) transformer-based LLMs demonstrated surprisingly high neural predictivity relative to their trained counterparts, which was interpreted as evidence that the transformer architecture may play a role in biasing computations to be more brain-like. Taken together, these findings paint a picture in which the artificial intelligence community is “rapidly converging on architectures that might capture key aspects of language processing in the human mind and brain"^2^.

Schrimpf et al.^2^ laid the groundwork for follow-up work which viewed LLMs not only as predictive tools, but also as candidate explanatory models of biological language processing. For example, Hosseini et al.^7^ demonstrated that LLMs achieve high neural predictivity even when the scale of natural language data they are trained on mirrors the developmental language exposure of humans. Aw et al.^8^ showed that instruction tuning LLMs enhances both their alignment with neural data and their integration of world knowledge. AlKhamissi et al.^9^ further investigated the neural predictivity of untrained transformer-based LLMs, attributing it to tokenization strategy and multi-headed attention. The authors proposed to “conceptualize language processing in the human brain as an untrained feature encoder providing representations to a downstream trainable decoder that produces language output".

While these studies represent the growing enthusiasm for using LLMs to gain neuroscientific insights, caution is warranted. LLMs are a relatively recent technology, and the field is still in the process of establishing robust methodologies to relate their representations to brain responses. Moreover, some researchers have noted that the excitement surrounding LLMs creates a drive to identify theoretically compelling parallels between artificial and biological language processing, sometimes at the risk of overlooking simpler confounds that may drive these mappings^10^. In this study, we empirically demonstrate that the results from Schrimpf et al.^2^ depend on non-robust methods and can be largely accounted for by simple confounds. Importantly, the issues we highlight have direct implications for several follow-up LLM-to-brain mapping studies^7–9,11–14^. Through this work, we hope to provide a course correction towards caution in drawing correspondences between LLMs and brains, and encourage future research that critically evaluates parallels between artificial intelligence and neuroscience. It is worth noting that many LLM-to-brain mapping studies do not exhibit these methodological issues and take care to account for potential confounds, as we summarize in Supplementary Table 1. Our study should therefore not be interpreted as a blanket critique of the field. Nonetheless, given the influence of Schrimpf et al.^2^ and its extensive follow-up work, we believe it is important to highlight these methodological pitfalls and offer a rigorous re-analysis of these results.

## Results

We evaluated the neural predictivity of several models on three neural datasets: (1) *Pereira2018* (fMRI - passages)^15^, (2) *Fedorenko2016* (ECoG - sentences)^16^, and (3) *Blank2014* (fMRI - stories)^17^. In *Pereira2018*, *n* = 10 participants read short passages presented one sentence at a time. Each sentence was presented for 4 s, followed by a 4 s fixation period, and sentence-level brain responses were extracted using a mass univariate analysis (see ‘Statistical testing’ for further details). *Pereira2018* consists of two sub-experiments, and we combined results across both experiments following Schrimpf et al.^2^. In *Fedorenko2016*, ECoG recordings were made while *n* = 5 participants read 52 sentences, presented word-by-word. In *Blank2014*, fMRI signals were acquired while *n* = 5 participants listened to 8 stories. We focus all analyses on language-selective voxels (*Pereira2018*) / electrodes (*Fedorenko2016*) / functional regions of interest (fROIs) (*Blank2014*). For additional details on these three neural datasets, see ‘Experimental data’.

When evaluating the neural predictivity of a model, we first passed as input to the model the same natural language stimuli that the participants received. We extracted model activations by aggregating across tokens corresponding to a given neural recording sample (see ‘LLM activation extraction and pooling’), and trained linear regressions to predict brain responses from each layer of the model.

To assess a model’s neural predictivity, we used nested cross-validation. In nested cross-validation, an outer loop partitions the data into an outer train set and test set, and an inner loop further partitions the outer train set into an inner train set and validation set. The validation set is used to select ridge and banded ridge regression hyperparameters, and the regression is then fit using these hyperparameters on the outer train set and evaluated on the test set. For models with multiple layers or hyperparameter settings, we focus our analyses on the layer/hyperparameter setting which achieves the highest neural predictivity on the test set (averaged across outer folds). Unless otherwise noted, all our results are generated by averaging values within voxels/electrodes/fROIs within each participant, and then reporting the mean of the participant averages.

### A baseline model of temporal autocorrelation achieves higher neural predictivity than *GPT2XL* when using shuffled splits

Most studies evaluating the neural predictivity of language models have employed contiguous train-test splits [e.g.,^4,5,18–22^], where a temporally contiguous chunk of brain responses (and the associated model activations) are held-out for testing. By contrast, several studies using the neural datasets we study here^7–9,11–13^, most notably Schrimpf et al.^2^, used shuffled splits, where samples are arbitrarily placed into the test set without regard for temporal adjacency. To provide an example with the *Pereira2018* dataset, contiguous splits imply that entire passages are held out for testing, whereas shuffled splits allow that some sentences from a given passage are placed in the train set, and other sentences from *the same passage* are placed in the test set.

Shuffled splits are known to be problematic in the field of language neural encoding because brain responses are temporally autocorrelated for reasons that are not entirely stimulus-driven^23^. A model can achieve high neural predictivity when using shuffled splits purely because it represents nearby time points similarly. To demonstrate this phenomenon, we constructed a baseline model which operates only on the principle that stimuli nearby in time should be assigned similar representations. We term this model the Orthogonal Autocorrelated Sequences Model (*OASM*) because the activations of *OASM* are orthogonal for distinct passages/sentences/stories, and autocorrelated for stimuli within a passage/sentence/story (see ‘Orthogonal Autocorrelated Sequences Model (OASM)’ for additional details). By construction, therefore, *OASM* exhibits zero neural predictivity under contiguous train-test splits.

**Fig. 1:**
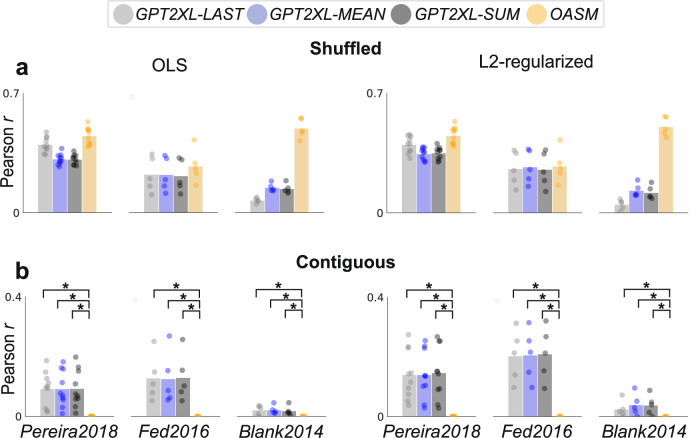
When using shuffled splits *OASM* predicts more neural variance than *GPT2XL.* **a** Neural predictivity of *OASM* and *GPT2XL* across three different activation extraction methods when using shuffled splits: last token pooling (*-LAST*), mean pooling (*-MEAN*), and sum pooling (*-SUM*). Each dot shows the mean predictivity across voxels/electrodes/fROIs for a given participant, and the bar displays the mean value across participants. The left side displays neural predictivity when using OLS regression, while the right side shows L2-regularized regression. **b** Same as (**a**) but when using contiguous splits. * denotes that *GPT2XL* performs significantly better (*α* = 0.05) than *OASM* across participants (*n* = 10, 5, 5 for *Pereira2018*, *Fed2016*, *Blank2014*) using a one-sided Wilcoxon signed-rank test. For both the OLS and Ridge models, p-values were 0.00098 for *Pereira2018* and 0.03125 for *Fedorenko2016*; for *Blank2014*, the p-value was consistently 0.03394 for all Ridge configurations and the base OLS model, and 0.03125 for the OLS mean and sum pooled variants. Source data are provided as a Source Data file.

We compared the neural predictivity of *OASM* to that of *GPT2XL* when using largely the same methodological choices implemented in Schrimpf et al.^2^: fitting the mapping between model activations and brain responses with ordinary least squares (OLS) regression and extracting activations from *GPT2XL* using the last token of the stimuli, which we term *GPT2XL-LAST*. The neural predictivity of *GPT2XL-LAST* with OLS regression is similar to what was reported in Schrimpf et al.^2^ before noise ceiling correction. *OASM* predicted brain responses qualitatively on-par with or better than *GPT2XL-LAST* across all three datasets, and there was no case where *GPT2XL-LAST* predicted brain responses significantly better than *OASM* (Fig. 1a, top left panel).

To evaluate the robustness of this finding, we compared the performance of *OASM* to *GPT2XL* when using two other activation extraction methods: (1) taking the mean across tokens (mean pooling, referred to as *GPT2XL-MEAN*), which was used in Kauf et al.^12^; and (2) taking the sum across tokens (sum pooling, referred to as *GPT2XL-SUM*), somewhat analogous to the process of convolving the hemodynamic response function (HRF) with model activations. Results were consistent, with *OASM* qualitatively achieving the same or higher neural predictivity than *GPT2XL-MEAN* and *GPT2XL-SUM* across all three neural datasets.

We hypothesized that the gap in neural predictivity between *GPT2XL* and *OASM* was partially due to the lack of regularization in OLS regression. Regularization tends to help higher-dimensional models, and a given layer of *GPT2XL* is higher-dimensional than *OASM*. However, while using L2-regularized regression reduced the gap between *OASM* and *GPT2XL* on *Pereira2018*, *GPT2XL* still did not explain significantly more neural variance than *OASM* on any of the three neural datasets (Fig. 1a, top right panel).

When switching to contiguous splits, the neural predictivity of *OASM* was 0, which is expected given that *OASM* represents distinct passages/sentences/stories orthogonally (Fig. 1b). The neural predictivity of *GPT2XL* decreased, but remained significantly above that of *OASM*. Here, too, L2-regularized regression led to higher neural predictivity for all *GPT2XL* activation extraction variants relative to OLS regression. For this reason, and because L2-regularized regression is the standard in the field^3–5,18–22^, we used this method for the remainder of our analyses.

### *OASM* accounts for the majority of the neural predictivity of *GPT2XL*

While *OASM* explains similar or more neural variance than *GPT2XL* when using shuffled splits, it remains unclear to what extent *OASM* accounts for the same neural variance that *GPT2XL* explains. Is the neural predictivity of *GPT2XL* on shuffled splits attributable to the fact that it simply represents nearby timepoints similarly?

We addressed this question in three ways. First, we examined whether the neural predictivities of *OASM* and *GPT2XL* were positively correlated with one another across voxels/electrodes/fROIs. Second, we performed a variance partitioning analysis at the participant-level using *R*^2^. In brief, we first quantified the neural predictivity of a model which combines activations from both *OASM* and *GPT2XL* (*OASM+GPT2XL*). We then quantified the fraction of the neural variance that *GPT2XL* explains that is not explained by *OASM* by subtracting the neural predictivity of *OASM* from that of *OASM+GPT2XL*, and dividing this difference by the neural predictivity of *GPT2XL* alone. Finally, we subtracted this fraction from 1 and converted it to a percentage to quantify the neural variance explained by *GPT2XL *that is also explained by *OASM*. This metric is termed Ω_*GPT*2*XL*_(*OASM*), or simply Ω (see ‘Percentage of LLM neural predictivity accounted for’). Third, we quantified the fraction of voxels in which *GPT2XL* explained significant additional variance beyond what OASM explained (i.e., where *OASM+GPT2XL* achieved significantly lower mean squared error than *OASM*).

**Fig. 2:**
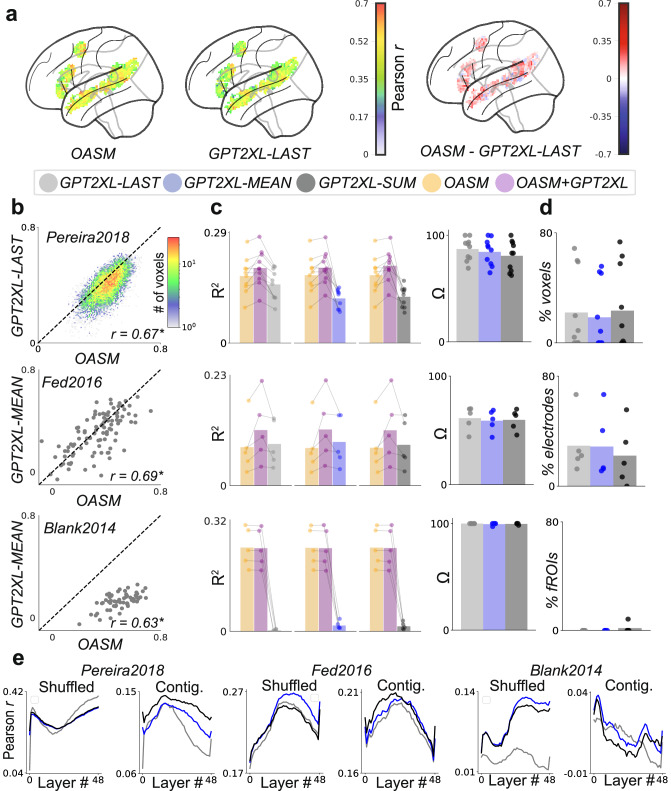
When using shuffled splits, *OASM* explains the majority of neural variance predicted by *GPT2XL.* All results are generated using shuffled splits, except for as noted in panel (**e**). **a** Glass brain plots showing Pearson *r* values across the left hemispheric language network in *Pereira2018*. Plots are generated by averaging Pearson *r* values within each voxel of the language network across all participants. Glass brain plots for individual participants are displayed in Supplementary Fig. 6a. **b**–**d** Top row is *Pereira2018*, middle row is *Fedorenko2016*, and bottom row is *Blank2014*. **b** Comparison of Pearson *r* values for *OASM* and *GPT2XL* at the voxel (*n* = 13553), electrode (*n* = 97), and fROI (*n* = 60) level. Bottom right corner shows the correlation in neural predictivity between the two models, with ^*^ indicating *p* < 0.05 using a two-sided test without adjustment for multiple comparisons (*p* = 5 × 10^−308^, 9 × 10^−15^, and 8 × 10^−8^ for *Pereira2018*, *Fed2016*, and *Blank2014*, respectively). A 2*D* histogram is used for *Pereira2018*, rather than a scatter-plot, due to the high number of voxels. **c** Left-side shows the neural predictivity of *OASM*, *OASM+GPT2XL*, and *GPT2XL* in *R*^2^. Lines connect dots from the same participant. Purple bars show *GPT2XL* banded onto *OASM* for each respective activation extraction method. These neural predictivity values are used to compute the percentage of *GPT2XL* neural predictivity that is accounted for by *OASM*, or *Ω*_GPT2XL_(*OASM*), displayed on the right side. **d** Percentage of voxels/electrodes/fROIs where *OASM+GPT2XL* explains significantly more neural variance than *OASM* alone after FDR correction within participant. **e** Across-layer neural predictivity of *GPT2XL* when using shuffled and contiguous splits. For all bar plots, each dot represents a participant, and each bar shows the mean across participants. Results in panels (**a**) and (**b**) are only shown for the best activation extraction variant of *GPT2XL*. Source data are provided as a Source Data file.

Across all three datasets, neural predictivity across voxels/electrodes/fROIs was correlated between *OASM* and *GPT2XL* (Fig. 2a, b). Furthermore, *OASM* accounted for over 80% of the neural variance that *GPT2XL* explains in *Pereira2018*, over 50% in *Fedorenko2016*, and nearly 100% in *Blank2014* (Fig. 2c). *OASM+GPT2XL* explained significant neural variance over *OASM* alone in around 20% of voxels, 30% of electrodes, and 0 fROIs (Fig. 2d; see also Supplementary Fig. 7a). Therefore, when using shuffled splits, the majority of the neural predictivity of *GPT2XL* is confounded with a model that simply represents nearby time points similarly.

### Across-layer neural predictivity patterns between shuffled and contiguous splits are anti-correlated

The previous analyses showed that shuffled splits are not a reliable method to assess neural predictivity. To what extent does switching from shuffled splits to contiguous splits impact LLM-to-brain mapping findings? To begin answering this question, we examined the pattern of neural predictivity across the layers of *GPT2XL* when using shuffled vs. contiguous splits. The across-layer pattern of neural predictivity is important because studies which employed shuffled splits using these datasets exclusively focus their analyses on either the LLM layer which achieves the highest neural predictivity^2,7,8,12^ or the last LLM layer^11^.

In *Pereira2018*, the neural predictivity across layers (*n* = 48) was highly anti-correlated between shuffled and contiguous splits: *r* = − 0.60, − 0.86, and  − 0.85 (all *p* < 0.05) for *LAST*, *MEAN*, and *SUM* (Fig. 2e). When using shuffled splits, the early and late layers of *GPT2XL* achieved the highest neural predictivity, whereas when using contiguous splits, the intermediate layers achieved the best neural predictivity. Because we selected the best layer of *GPT2XL* for each of the two experiments in *Pereira2018* separately, we also show that these same trends hold when evaluating across-layer patterns within each experiment separately (Supplementary Fig. 1b). In *Fedorenko2016*, the across-layer trends were more similar between shuffled and contiguous splits (*r* = 0.61, 0.57, 0.52; all *p* < 0.05), with later intermediate layers generally performing the best on shuffled splits and early intermediate layers performing the best on contiguous splits (Fig. 2e). Finally, in *Blank2014*, the across-layer performance was also anti-correlated between shuffled and contiguous splits for *GPT2XL-MEAN* and *GPT2XL-SUM* (*r* = 0.46, − 0.65, − 0.44; all *p* < 0.05); later intermediate layers performed the best when using shuffled splits, whereas the early layers performed the best when using contiguous splits (Fig. 2e). Thus, on 2 out of the 3 datasets, the pattern of neural predictivity across layers flips between shuffled and contiguous splits.

### Key model comparison results do not replicate with contiguous splits

We next asked whether key model comparison analyses from Schrimpf et al.^2^ hold under contiguous splits. Of the models evaluated by Schrimpf et al.^2^, we use 28 of the 29 bidirectional transformer models, 6 of the 9 unidirectional transformer models (excluding those that were previously mislabeled as bidirectional, see ‘Language models’), and 2 of the 3 static word embedding models. We also added more recent, unidirectional models: 4 transformers (*Llama-3.2* family) and 4 RNNs (*RWKV-4* family). Schrimpf et al.^2^ reported that unidirectional transformer models (specifically those from the *GPT2* family) predicted neural responses better than both bidirectional transformers and recurrent models, concluding that unidirectional transformers were uniquely “brain-like”. However, these findings relied on shuffled splits and last-token activation extraction, which may have introduced biases.

**Fig. 3:**
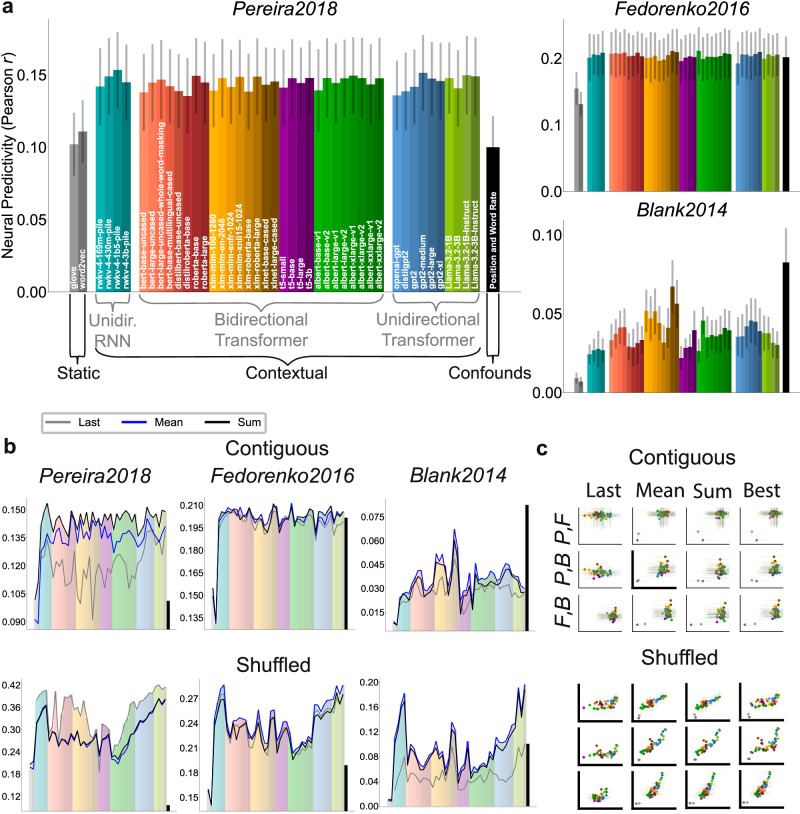
Model comparisons are not robust to activation extraction and splitting choices. **a** Neural predictivity shown across a wide array of models when using contiguous splits and the best-performing activation extraction method for each model. Bars and error bars correspond to the mean and standard error of the mean across participants, respectively (*n* = 10 for *Pereira2018*, *n* = 5 for *Fedorenko2016*, *n* = 5 for *Blank2014*). **b** Lines represent neural predictivity for each activation extraction method. Colored bars depict best-performing activation extraction method. Top row shows results when using contiguous splits; bottom row shows shuffled splits. Note that the y-axes for these plots do not necessarily begin at 0. **c** Correlation in neural predictivity between datasets. Scatter plots depicting the relation in neural predictivity trends across models between each pair of datasets. *P*, *F*, and *B* refer to *Pereira2018*, *Fedorenko2016*, and *Blank2014*, respectively. Rows are labeled with the datasets plotted along the *x* and *y* axes, respectively. Columns correspond to different activation extraction methods. Axes are bolded if the Pearson correlation is significant (*p* < 0.05) when static embedding models are excluded (*n* = 42 models). All correlations are significant when static embedding models are included (*n* = 44 models). Note that neural predictivity scores are not reported for static embedding models with the last-token method, as this method is ill-suited for non-contextual models. Individual per-participant scores are shown for all datasets and evaluation settings in Supplementary Fig. 4. Exact p-values are shown in Supplementary Table 4. All significance tests are one-sided tests of Pearson correlation, without adjustment for multiple comparisons. Source data are provided as a Source Data file.

Using contiguous splits and the best-performing activation extraction method for each model, we do not find that unidirectional transformers consistently achieve higher neural predictivity scores than bidirectional transformers on any dataset, nor do we find that they consistently outperform unidirectional RNNs (Fig. 3a). We do replicate the finding from Schrimpf et al.^2^ that static word embedding models consistently exhibit lower neural predictivity than all contextual models (i.e., LLMs) across all datasets.

However, the gap in predictivity between contextual models and static embeddings might reflect factors other than contextual processing of linguistic content. Here, we introduce a simple baseline model which encodes only Positional signals and Word Rate information (*PWR*, see ‘Positional signals and word rate’ for more details). Positional signals encode stimuli by their position within a passage, sentence, or story (unlike *OASM*, stimuli across passages, sentences, or stories are not represented orthogonally). While *PWR* matches static models in the *Pereira2018* dataset, it rivals contextual models in the *Fedorenko2016* dataset and outperforms them in the *Blank2014* dataset, revealing the potential impact of simple confounds in these datasets (Fig. 3a).

We next examined how the choice of activation extraction method can influence model comparisons. When examining the neural predictivity of each model under each method (Fig. 3b top, per-participant results shown in Supplementary Fig. 4), we find that the last-token method biases the results in the *Pereira2018* dataset against bidirectional models. We assessed significance between each unidirectional transformer model and each bidirectional transformer model across participants (see 'Statistical testing') (Supplementary Fig. 5). Whereas 51.1% of comparisons showed significantly greater neural predictivity for unidirectional transformer models when using the commonly used last-token activation extraction method, less than 10% of comparisons were significant when using either mean pooling, sum pooling, or the best activation extraction method per model. Hence, even under contiguous splits, the choice of activation extraction method can greatly affect model comparison results. This observation is typically underappreciated in the field, as the majority of LLM-to-brain mapping findings depend on a single activation extraction method (Supplementary Table 1).

Compared to contiguous splits, in shuffled splits (Fig. 3b bottom) a greater fraction of comparisons between unidirectional transformers and all other classes of models (bidirectional transformers, unidirectional RNNs, and static word embeddings) were significant, for all activation extraction methods on all datasets (Supplementary Fig. 5). This finding suggests that the use of shuffled splits drove the apparent superiority of unidirectional transformer models in Schrimpf et al.^2^.

### Relative performance trends across LLMs are uncorrelated between datasets when using contiguous splits

Schrimpf et al.^2^ also reported that relative performance trends across models were consistent between datasets. Following their analyses, we examined whether this pattern held under contiguous splits, by calculating Pearson correlations across model neural predictivity scores (Pearson *r*) between datasets (Fig. 3c). Using the best activation extraction method for each model, significant correlations emerged when all models were included, but vanished when static word embedding models were excluded. This pattern persisted when using the same activation extraction method across all models: significant correlations were always found when including all models, but when excluding the two static word embedding models, a significant correlation was found in only 1 out of 9 cases—between *Pereira2018* and *Blank2014* when mean-pooling. Under shuffled splits, significant correlations appeared universally across datasets and activation extraction methods, regardless of whether static models were included. Thus, shuffled splits in Schrimpf et al.^2^ likely drove the consistent trends in neural predictivity across LLMs between datasets.

### Correlation between neural predictivity and next-word prediction does not hold under contiguous splits

**Fig. 4:**
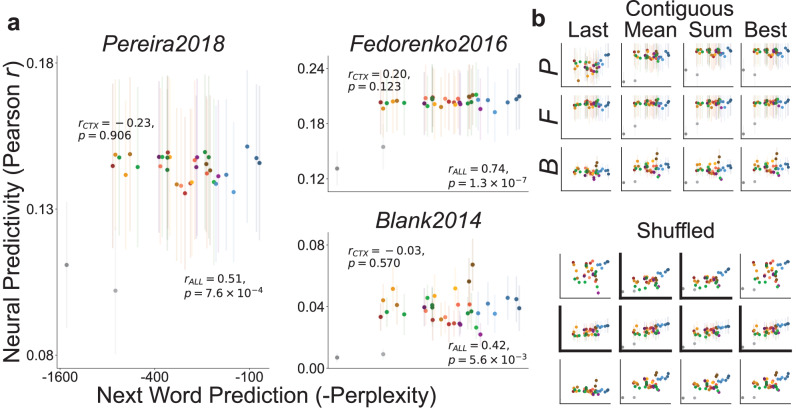
Correlation between neural predictivity and next-word prediction ability across LLMs depends on shuffled splits on these datasets. **a** Scatter plots depict the relation between next-word prediction and neural predictivity on each dataset when using contiguous splits and the best-performing activation extraction method for each model. Pearson correlations and p-values are given when including all models (*r*_*ALL*_) and when only including contextual models (*r*_*CTX*_). **b** Scatter plots depict the relation between next-word prediction and neural predictivity across models for each dataset (rows) with each activation extraction method (columns) with contiguous (top) and shuffled (bottom) splits. Axes are bolded when *r*_*CTX*_ is significant (*p* < 0.05, *n* = 34). *r*_*ALL*_ is significant in all cases where applicable (*n* = 32); *r*_*ALL*_ is not defined when using the last-token method as static embedding model scores are omitted in this case. Exact p-values are shown in Supplementary Table 5. All significance tests are one-sided tests of Pearson correlation, without adjustment for multiple comparisons. Source data are provided as a Source Data file.

Finally, we reassessed the most impactful result from Schrimpf et al.^2^: the relationship between an LLM’s next-word prediction (quantified as negative perplexity) and its neural predictivity. We only tested the 36 models that were also used by Schrimpf et al.^2^, and used the same next-word prediction values they had previously reported (see ‘Next-word prediction metric’ for more detail). Using contiguous splits and the best activation extraction method per model, we found significant correlations in all datasets only when static word embedding models were included. When restricted to contextual models, these correlations were not significant (Fig. 4a). These results were consistent across all activation extraction methods (Fig. 4b), suggesting that the correlation between neural predictivity and next-word prediction on these datasets is not robust, and is driven by a difference between static word embedding models and contextual models. By contrast, when using shuffled splits, the correlation between neural predictivity and next-word prediction across contextual models appeared in 6 out of 12 cases. (Fig. 4a).

Even when separately analyzing unidirectional and bidirectional model classes, we still found minimal evidence for correlations between next-word prediction and neural predictivity under contiguous splits. Among bidirectional models, no significant correlations emerged; for unidirectional models, only one significant correlation was observed (*p* = 0.022)—when using the *Pereira2018* dataset with last-token feature extraction.

### Positional information, word rate, and static embeddings account for the majority of the neural predictivity of trained LLMs

Our model comparison analyses reveal that Position and Word Rate (*PWR*), as well as static word embedding models, perform surprisingly well relative to LLMs. Motivated by this finding, we quantified the percentage of the neural predictivity of LLMs that was accounted for by *PWR* and static models. We used *GloVe* and *GPT2XL* as representative examples of static and contextual models, respectively.

**Fig. 5:**
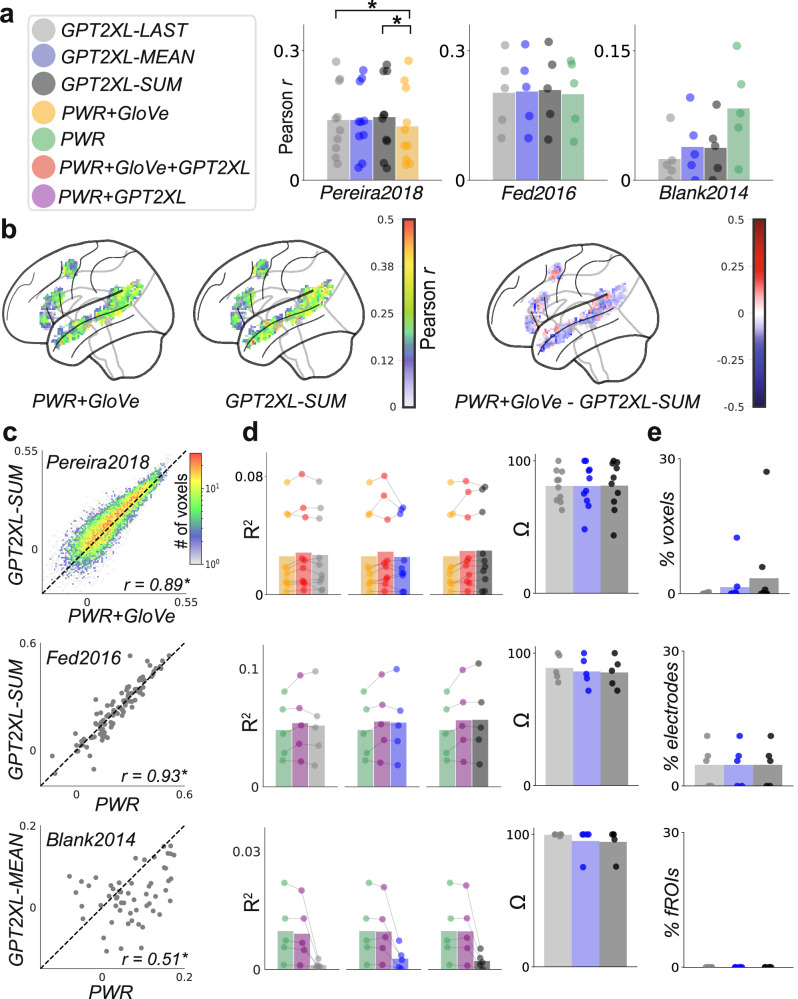
Positional information, word rate, and *GloVe* explain the majority of *GPT2XL* neural predictivity. All results are generated using contiguous splits. **a** Neural predictivity of different models, in Pearson *r*, for each neural dataset. Asterisks indicate that *GPT2XL* neural predictivity is significantly higher than that of *PWR+GloVe* / *PWR* (*α* = 0.05) across participants (*n* = 10, 5, 5 for *Pereira2018*, *Fed2016*, *Blank2014*), with *p* = 0.0020 and *p* = 0.0097 for *GPT2XL-LAST* and *GPT2XL-SUM* on *Pereira2018* when using a one-sided Wilcoxon signed-rank test. **b** Glass brain plots showing Pearson *r* values across the left hemisphere of the language network in *Pereira2018*. Plots are generated by averaging Pearson *r* values within the language network across all participants. Glass brain plots for individual participants are displayed in Supplementary Fig. 6b. **c** 2*D* histogram (for *Pereira2018*) or scatter-plot (for *Fedorenko2016* and *Blank2014*) comparing Pearson *r* values for *PWR+GloVe* / *PWR* and *GPT2XL* at the voxel (*n* = 13553), electrode (*n* = 97), and fROI (*n* = 60) level. Bottom right corner shows the correlation in neural predictivity between the two models, with ^*^ indicating *p* < 0.05 using a two-sided test without adjustment for multiple comparisons (*p* = 5 × 10^−308^, 9 × 10^−39^, and 3 × 10^−5^ for *Pereira2018*, *Fed2016*, and *Blank2014*, respectively). **d** Left side shows the neural predictivity of *PWR+GloVe* / *PWR*, *PWR+GloVe+GPT2XL* / *PWR+GPT2XL*, and *GPT2XL* with *R*^2^. Lines connect dots from the same participant. These neural predictivity values are used to compute percentage of *GPT2XL* neural predictivity that is accounted for by *PWR+GloVe / PWR* displayed on the right side. **e** Percentage of voxels/electrodes/fROIs where *PWR+GloVe+GPT2XL* / *PWR+GPT2XL* explains more neural variance than *PWR+GloVe* / *PWR* alone. For all bar plots, each dot shows values for a given participant, and bars show means across participants. Results in panels (**b**) and (**c**) are only shown for the best activation extraction variant of *GPT2XL*. Source data are provided as a Source Data file.

We first fit a joint regression with activations from both *PWR* and *GloVe* (*PWR+GloVe*) for each of the three neural datasets. *PWR+GloVe* achieved better neural predictivity than either model alone on *Pereira2018* (Supplementary Fig. 1a); on *Fedorenko2016* and *Blank2014*, *PWR+GloVe* did not achieve better neural predictivity than *PWR* alone, and so we used *PWR* for further analyses with these datasets. Across voxels/electrodes/fROIs, the neural predictivity of these baseline models (*PWR+GloVe* / *PWR*) was strongly correlated with the neural predictivity of *GPT2XL* (Fig. 5b, c). Furthermore, they account for most of the neural variance explained by *GPT2XL*: over 85% in *Pereira2018*, over 80% in *Fedorenko2016*, and nearly 100% in *Blank2014*, respectively. Finally, adding *GPT2XL* to the baseline models significantly improved brain predictivity in only around 3.4% of voxels, around 4.7% of electrodes, and no fROIs (see also Supplementary Fig. 7b). We replicated these key findings when using three other LLMS, *RoBERTa-Large*, *RWKV*, and *Llama* (Supplementary Fig. 2).

A previously explored question on *Pereira2018* is whether the mapping between LLMs and brains reflects lexical-semantics or syntactic information^12^. We examined how much of the neural variance explained by *GloVe*, a model of lexical-semantic content, could be accounted for by *PWR*. We found that Ω_GloVe_(*PWR*) was around 55% and that *PWR+GloVe* explained significant neural variance over *PWR* only in 7.6% of voxels (Supplementary Fig. 3). An LLM-derived model of syntactic representations, which was constructed by averaging LLM representations from sentences with the same part-of-speech and dependency tags but different content words (*SYNTAX*; see ‘Syntax’), explained even less neural variance over these simple confounds: Ω_SYNTAX_(*PWR*) was over 80%, and the *PWR+SYNTAX* model only explained significant neural variance over *PWR* in 0.00066% of voxels. Hence, lexical-semantic and syntactic representations are strongly confounded with simpler factors in *Pereira2018*, so this dataset is not well-suited for disentangling the linguistic features underlying LLM neural predictivity.

In summary, a combination of positional signals, word rate, and static word embeddings predicts almost as much—or more—neural variance than *GPT2XL*. Furthermore, in all three datasets, these models account for upwards of 80% of the neural variance that *GPT2XL* explains, and these results replicate with three other LLMs. These findings suggest that even when using contiguous splits, there are relatively simple explanations underlying the neural predictivity of LLMs on these datasets.

### Positional information and word rate fully account for the neural predictivity of Untrained GPT2XL

Having accounted for the majority of the neural variance explained by trained LLMs, we next examined whether *PWR* could account for the neural variance explained by untrained *GPT2XL* (*GPT2XLU*). Previously, the surprisingly high neural predictivity of untrained transformers led some to conclude that the transformer architecture itself biases computations to be more brain-like^2^, and inspired the development of a novel architecture that reportedly achieved state-of-the-art neural and behavioral alignment across several datasets^9^. We hypothesized that, when evaluated on contiguous (cf. shuffled) splits, the neural predictivity of *GPT2XLU* could be fully accounted for by *PWR*.

**Fig. 6:**
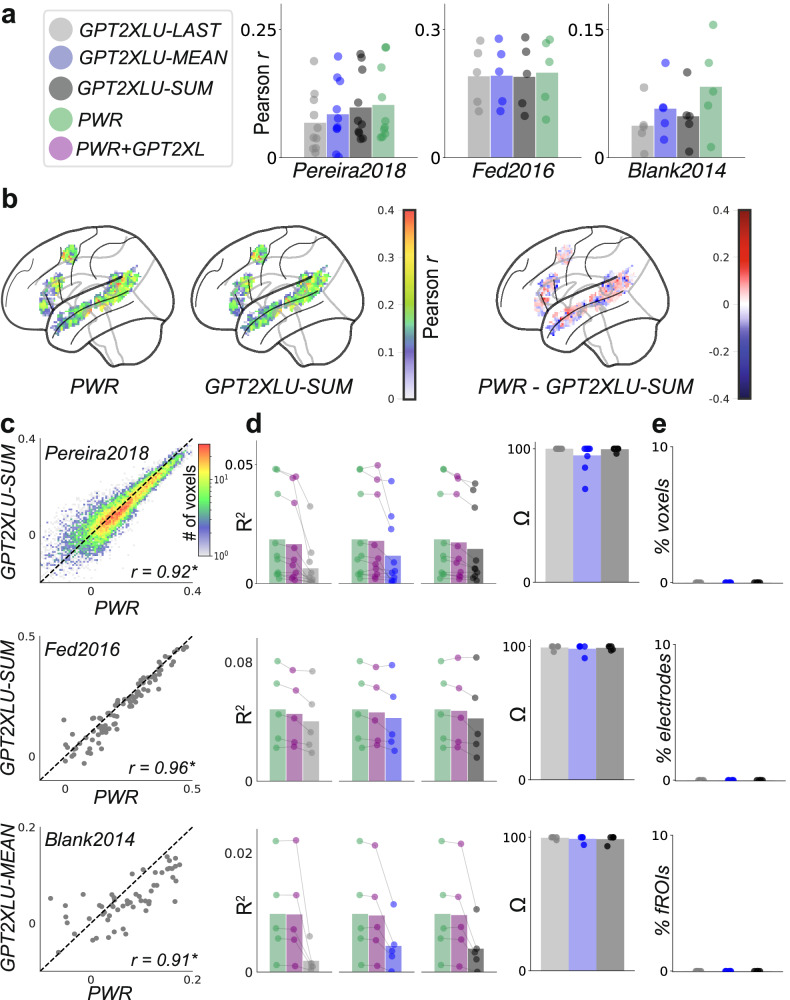
Positional information and word rate fully account for untrained *GPT2XL* neural predictivity. All results are generated using contiguous splits. **a** Neural predictivity of different models, in Pearson *r*, for each neural dataset. **b** Glass brain plots showing Pearson *r* values across the left hemisphere of the language network in *Pereira2018*. Plots are generated by averaging Pearson *r* values within the language network across all participants. Glass brain plots for individual participants are displayed in Supplementary Fig. 6c. **c** 2*D* histogram or scatter-plot comparing Pearson *r* values for *PWR* and *GPT2XL* at the at the voxel (*n* = 13553), electrode (*n* = 97), and fROI (*n* = 60) level. Bottom right corner shows the correlation in neural predictivity between the two models, with ^*^ indicating *p* < 0.05 using a two-sided test without adjustment for multiple comparisons (*p* = 5 × 10^−308^, 2 × 10^−54^, and 4 × 10^−16^ for *Pereira2018*, *Fed2016*, and *Blank2014*, respectively). **d** Left side shows the neural predictivity of *PWR*, *PWR+GPT2XL*, and *GPT2XL* with *R*^2^. Lines connect dots from the same participant. These neural predictivity values are used to compute percentage of *GPT2XL* neural predictivity that is accounted for by *PWR* displayed on the right side. **e** Percentage of voxels/electrodes/fROIs where *PWR+GPT2XLU* explains significantly more neural variance than *PWR* alone. For all bar plots, each dot shows values for a given participant, and bars show mean across participants. Results in panels (**b**) and (**c**) are only shown for the best activation extraction variant of *GPT2XLU*. Source data are provided as a Source Data file.

Supporting our hypothesis, *PWR* performed on par with *GPT2XLU* across all three neural datasets (Fig. 6a). Furthermore, the neural predictivity of *PWR* and *GPT2XLU* was strongly correlated across voxels/electrodes/fROIs (Fig. 6b, c). *PWR* accounted for essentially all ( >98%) of the neural variance of *GPT2XLU* in each dataset, and there were no voxels/electrodes/fROIs where *PWR+GPT2XLU* explained significant neural variance over *PWR* (see also Supplementary Fig. 7c). We conclude that a combination of positional signals and word rate information explain nearly all of the neural variance that *GPT2XLU* explains.

## Discussion

Our rigorous re-evaluation of LLM-to-brain mappings on three neural datasets offers four main conclusions. First, shuffled splits—which were used in one of the most impactful studies on LLM-to-brain mappings^2^, as well as in multiple subsequent studies^7–9,11–14^—are not a robust method to assess neural predictivity in language stimuli with temporal autocorrelation. Second, when switching to contiguous splits and L2-regularized regression (standard methodological choices), the main results reported in Schrimpf et al.^2^ based on trained LLMs were not robust: unidirectional transformers did not exhibit better neural predictivity than other classes of LLMs; differences in predictivity across LLMs were not consistent across neural datasets; and, critically, next-word prediction ability was not robustly correlated with neural predictivity. Third, positional signals and word rate (*PWR*) achieved neural predictivity on par with *GPT2XL* on two out of three neural datasets, and achieved similar or better neural predictivity relative to untrained GPT2XL (*GPT2XLU*) on all datasets. Finally, a combination of *PWR* and static word embeddings (*GloVe*) accounted for over 80% of the neural variance that *GPT2XL* explains, and *PWR* alone accounts for essentially all the neural variance that *GPT2XLU* explains.

Among the eight studies we identified as employing shuffled splits, only one—Kauf et al.^12^—clearly stated this choice; the others typically do not state how cross-validation folds are constructed. Importantly, the effect whereby shuffled splits “inflate" neural predictivity is not uniform, so it can dramatically alter patterns of difference in predictivity between layers and across models, jeopardizing conclusions that follow from such layer or model comparisons. However, the impact of shuffled splits is more severe on the two fMRI datasets relative to the electrophysiological dataset used here (*Fedorenko2016*).

Our findings demonstrate that the susceptibility of shuffled splits to temporal autocorrelation is substantial, as the majority of neural variance explained by *GPT2XL* was confounded with *OASM*. This disadvantage outweighs the potential merit of shuffled over contiguous splits, namely, increased coverage of the semantic space in the training set (Kauf et al.^12^). Additionally, this benefit appears limited in the *Pereira2018* dataset, because it includes multiple passages for each semantic topic, which can be assigned to training vs. test sets; by designing our splits to leverage this feature (see ‘Train, validation, and test folds’), we demonstrate that contiguous splits can effectively balance semantic diversity and reliable estimation of model performance.

We emphasize that, although the majority of language encoding studies using these three neural datasets have employed shuffled splits, this practice is not pervasive in studies using other neural datasets. Out of the 28 language encoding studies we reviewed that used other neural datasets (Supplementary Table 1), only one study used shuffled splits: Mischler et al.^14^.

Our findings also underscore the importance of systematically comparing activation extraction methods from LLMs when fitting encoding models in fMRI datasets, where each neural sample corresponds to multiple tokens (words or sub-words) (see also Kauf et al.^12^). For instance, in *Pereira2018*, the most commonly used approach—last-token extraction—performed the worst, and particularly disadvantaged bidirectional (cf. unidirectional) transformers. Thus, patterns of alignment between LLMs and brains are influenced by the assumptions inherent to the chosen activation extraction method (see also Jain et al.^24^). The choice of activation extraction method is less relevant for electrophysiological datasets due to high temporal resolution.

Critically, even after addressing the methodological concerns above, we found that positional signals and word rate accounted for much of the neural predictivity of LLMs in the three datasets analyzed. Although these confounds were not accounted for in other studies using these same datasets, several studies using different datasets have accounted for a variety of confounds. We provide a comprehensive review of such controls in Supplementary Table 1. However, the consideration of positional confounds specifically has been relatively limited. Antonello et al.^5^ were the first to highlight this issue, identifying positional signals in the initial 100 seconds of narrative stimuli and, consequently, removing those samples from the testing set. We observed similar effects at the beginnings of stories in the *Blank2014* dataset, and the neural variance predicted by *GPT2XL* was largely confounded with these signals. Positional confounds may therefore be present across narrative comprehension datasets. In contrast, in *Pereira2018* and *Fedorenko2016* the positional signals we modeled are likely entangled with the structure of the linguistic materials themselves, e.g., a consistent organization of information across paragraphs in *EXP2* in *Pereira2018*, and a similar syntactic structure across many sentences in *Fedorenko2016*). Moreover, in the latter dataset, neural activity in many electrodes increases across word positions and reflects the construction of meaning^16^. Hence, it is unclear whether the brain responses in *Pereira2018* and *Fedorenko2016* that are predicted by positional signals reflect position-correlated linguistic content, genuine representations of position, or both.

The finding that simple confounds explained much of the neural predictivity of LLMs in *Pereira2018* (and the other datasets) suggests that this dataset is not well-suited for isolating which linguistic features drive LLM neural predictivity (e.g.,^11,12^) because such features are highly confounded with much simpler explanations. For instance, we found that the neural variance predicted by LLM-derived syntactic representations could be almost entirely accounted for by positional signals and word rate information, and these confounds also accounted for the majority of the neural variance that a lexical-semantic model, *GloVe*, explained.

More broadly, our study provides an updated view regarding the extent to which the “brain-likeness” of LLMs is apparent in the datasets of Schrimpf et al.^2^. Our conclusions are scoped to the three neural datasets analyzed in that study (*Pereira2018, Fedorenko2016, Blank2014*). On these datasets, and using a standardized pipeline (contiguous splits, appropriate pooling of model activations, and L2-regularized regression), the central claim of Schrimpf et al.^2^—that models with better next-word prediction are those that best predict brain responses—does not hold. We emphasize that this finding does *not* imply a field-general failure. Outside the Schrimpf et al.^2^ datasets, the literature is somewhat mixed on the relationship between next-word prediction ability and neural predictivity, though leaning positive. On the one hand, Pasquiou et al.^25^ report such a relationship *within* but not *across* model classes, and Caucheteux and King^4^ show that, throughout LLM training, neural predictivity can eventually decouple from next-word prediction. On the other hand, studies with larger samples and longer, more naturalistic narratives often report positive correlations between next-word prediction ability and neural predictivity across pretrained LLMs^19,26,27^. Our analyses do not adjudicate those settings; rather, they clarify that, in the datasets used by Schrimpf et al.^2^, these results do not hold. In addition to next-word prediction abilities of LLMs, other performance metrics have also been reported to correlate with neural predictivity by studies using shuffled splits, such as an LLM’s integration of world knowledge following instruction-tuning^8^, or its performance on semantic benchmark tasks^14^. Crucially, given our finding that the relationship between neural predictivity and one model performance metric (next-word prediction ability) can change substantially when shifting from shuffled to contiguous splits, we strongly recommend evaluating whether these other relationships are robust under contiguous splits.

A second major claim in Schrimpf et al.^2^ is that unidirectional transformers can achieve high neural predictivity when trained on no linguistic input, and thus that these models are endowed with architectural biases that lead to “brain-like" computations (see also AlKhamissi et al.^9^). However, when using contiguous splits, positional signals and word rate fully account for the neural predictivity of the untrained GPT2XL. Moreover, Hosseini et al.^7^ showed that *GPT2*-style models can achieve high neural predictivity on *Pereira2018* when trained on developmentally realistic amounts of data. We found that when using shuffled splits, a model that is trained on no text and is based solely on passage/sentence/story boundaries (*OASM*) achieves comparable neural predictivity to a fully trained *GPT2XL*, suggesting that such predictivity is not indicative of a model’s developmental plausibility.

Beyond accounting for the neural predictivity of untrained models, positional information and word rate, along with static word embeddings, accounted for the majority of neural variance across four classes of trained LLMs. This finding, along with the fact that LLMs of all classes exhibit similar neural predictivity, suggests that trained LLMs are all mapping to similar features on these neural datasets (for a similar claim, albeit based on analyses with shuffled splits, see Hosseini et al.^13^).

There are six important limitations to consider when interpreting the results of our study. First, all our results are conducted using neural datasets in which participants read short passages, read isolated sentences, or listened to short stories which are designed to have complex, low-frequency syntactic structures. By contrast, many recent language encoding studies have participants listen to engaging stories and collect much more data per participant (e.g.,^28^), or contain data from many more participants (e.g.,^29^). Second, although we used ridge and banded ridge regression to mitigate overfitting when using large models (e.g., *GPT2XL*) or model combinations (e.g., *PWR+GloVe+GPTXL*), overfitting is still a concern for large models due to noise in neural data and low sample sizes. This issue may disadvantage the neural predictivity of LLMs relative to simple models, as well as disadvantage larger LLMs relative to smaller LLMs. Third, our Ω metric can become unstable when the neural variance that an LLM explains is near 0 (as in *Blank2014*), because that variance is in the denominator of this metric. Fourth, as was done in Schrimpf et al.^2^, we selected the best LLM layer based on test set performance. To ensure a fair comparison, we also selected hyperparameters for the simple models (*OASM* and positional signals) using the test set, with the number of hyperparameter choices matched to the number of *GPT2XL* layers. Although using the test set for hyperparameter selection is not ideal, our results are nearly identical when using the validation set for hyperparameter selection (Supplementary Table 2).

Fifth, following Schrimpf et al.^2^ and follow-up studies, we restricted analyses to individually defined fROIs in the language network, as identified with a functional localizer task. While these regions show reliable variation across sentences (Fedorenko et al.^30^), and such variation correlates with psycholinguistic variables (e.g., Shain et al.^31^), voxels with rich time-varying signal may fall outside these fROIs. It is possible that simple baselines, specifically position and word rate, are particularly advantaged within our fROIs relative to other regions. Finally, we used the same preprocessed datasets as used by Schrimpf et al.^2^, which followed generally acceptable preprocessing practices, including removal of slow drifts and motion correction (see ‘Preprocessing details’). Nonetheless, because we do not rigorously investigate these preprocessing choices, it is possible that some of our findings might depend on these choices.

Our study joins an emerging literature in cautioning against over-interpreting a model’s high neural predictivity as evidence that the model shares theoretically interesting properties with brains^10,19,32–34^. Large-scale analyses like ours, which incorporate diverse datasets, models, and methodologies, are essential for critically evaluating whether any specific model attribute is robustly associated with neural predictivity. Such analyses have proven similarly insightful in the visual domain, where they have likewise highlighted how findings depend on methodological choices^35^ and revealed a striking similarity in the neural predictivity of deep learning models regardless of their training objective or architecture^36^. We hope that through more rigorous evaluations, the field will come to an accurate view of the correspondence between LLMs and brains.

## Methods

### Experimental data

For all three datasets, we used the same versions as used by ref. ^2^. All analyses were done on language-selective voxels/electrodes/fROIs (see Supplementary for a description of functional localization).

#### *Pereira2018* (fMRI)

*Pereira2018* is composed of two experiments. Experiment 2 (EXP2) consists of 96 passages each containing 4 sentences (384 sentences total), with *n* = 9 participants. Experiment 3 (EXP3) consists of 72 passages each consisting of 3 or 4 sentences (243 sentences total), with *n* = 6 participants. Passages in each experiment were evenly divided into 24 semantic categories which were not related across experiments (4 passages per category in EXP2, and 3 passages per category in EXP3). Each sentence was presented for 4 s, followed by a 4 s fixation period, with an additional 4 s between passages. Thus, individual sentences were separated by fixed intervals of 8 s for sentences within the same passage and 12 s at passage boundaries. Participants were not prompted to provide any input (e.g., button press) upon sentence completion. We focus our results on voxels from within the “language network" as identified by Schrimpf et al.^2^ using a separate “localizer" task. Data from EXP2 consisted of a 384 × 12155 matrix (sentences  × voxels) and data from EXP3 consisted of a 243 × 8031 matrix. For all analyses except for voxel-wise statistical testing (see ‘Statistical testing’), we analyze each voxel in each experiment separately, and then combine the results across voxels and experiments as done in refs. ^2,7,8,12^. There were 13553 unique language network voxels across both experiments.

#### *Fedorenko2016* (ECoG)

Participants (*n* = 5) read 52 sentences, each consisting of 8 words, one word at a time (416 words total). A total of 97 language-responsive electrodes were identified across 5 participants: 47, 8, 9, 15, and 18, for participants 1 through 5, respectively. High gamma activity was extracted from the neural recordings, and responses were temporally averaged across the full presentation time-window of each word. The entire dataset consisted of a 416 × 97 matrix (words  × electrodes).

#### *Blank2014* (fMRI)

*Blank2014* consisted of 5 participants listening to 8 stories from the publicly available Natural Stories Corpus^37^. An fMRI volume was acquired every 2 s, resulting in a total of 1317 TRs across the 8 stories. fMRI BOLD signals were averaged across voxels within each functional region of interest (fROI) in the language network. There were 12 fROIs per participant (6 per hemisphere) for a total of 60 fROIs across all 5 participants, resulting in a 1317 × 60 matrix.

### Language models

For the majority of our analyses, we focus on *GPT2XL*^6^. We do so because *GPT2XL* was shown to be the best-performing model on *Pereira2018*, and performed on par or better than other models in *Fedorenko2016* and *Blank2014*^2^. Additionally, it has been the main language model used in other neural encoding studies using these datasets^7,12^. *GPT2* is a unidirectional transformer model, meaning that it can only attend to current and past tokens (but not future tokens). It is trained on next-token prediction. The *XL* variant has  ~ 1.5*B* parameters and 48 layers, with an embedding dimensionality of 1600. We additionally replicate our findings in Fig. 5 with three other models: *RoBERTa-Large*, *Llama-3.2-3B-Instruct*, and *RWKV-4-3B-Pile*.

Due to differences in methodological choices with Schrimpf et al.^2^, we also tested 45 models to re-evaluate their encoding performance, and the correlation between encoding performance and next-word prediction ability across those models. Out of the 45 models we used, 36 were also used in Schrimpf et al.^2^. We omitted 7 models from Schrimpf et al.^2^: 3 models were wrongly labeled as bidirectional models when they were in fact unidirectional (*Transfo-XL-WT103*, *XLM-CLM-EnFr-1024*, *CTRL*), and we replaced them with more recent models from the *Llama-3.2* family, including instruction-tuned variants; 2 recurrent models (*LSTM-LM1B* and *Skip-Thoughts*) were replaced with models from the *RWKV-4* family, which are more comparable in terms of their scale and performance on language tasks to the other transformer-based LLMs; 1 bidirectional model, *T5-11b*, was not included due to memory constraints; and 1 word embedding model, *ETM*, was not included because it was trained on 4 to 5 orders of magnitude less text than *GloVe* or *word2vec*. We used the HuggingFace Transformers package to implement these language models Wolf et al.^38^.

### LLM activation extraction and pooling

LLMs provide a representation for each token, which is either a sub-word or complete word. However, the brain responses in *Pereira2018* and *Blank2014* are acquired at a much slower temporal resolution, and so the token representations of the LLM need to be pooled to match the temporal resolution of the brain responses. The need for pooling also applies to *Fedorenko2016* in cases where tokens correspond to sub-words, because brain responses in *Fedorenko2016* are provided for each word. For all three neural datasets, we extract features from LLMs in three distinct ways: last token pooling, mean pooling, and sum pooling. We describe these methods in more detail for each dataset below. We use last token pooling because it was used in Schrimpf et al.^2^ and Kauf et al.^12^; mean (average) pooling because it was included in Kauf et al.^12^; and sum pooling because it is more analogous to the word-level impulse response model commonly used in more naturalistic neural encoding studies^18,24^, and the HRF convolved with the brain responses to each word is similar to summing the model activations for each word.

#### Pereira2018

To extract the representation of a single sentence, we fed an LLM all words from the beginning of that sentence’s passage until and including the sentence itself. Because each neural sample provided in the dataset of Schrimpf et al.^2^ corresponded to a single sentence, we converted LLM token-level representations to sentence-level embeddings by either taking the representation at the last token (which is always a period) of the sentence (last token pooling), taking the mean across tokens within the current sentence (mean pooling), or summing across tokens within the current sentence (sum pooling).

#### Fedorenko2016

For each word, its corresponding tokens were fed into the LLM together with all preceding tokens in the sentence as context. Because the brain response is averaged per word, we converted LLM token-level embeddings to word embeddings by either taking the last token for multi-token words (last token pooling), averaging across tokens in multi-token words (mean pooling), or summing across tokens in multi-token words (sum pooling). We left single-token words unmodified.

#### Blank2014

Following Schrimpf et al.^2^, we used a version of the text that was binned into chunks corresponding to each TR. The last word in each chunk of text was approximately 4 s before the corresponding TR, reflecting the delay of the hemodynamic response. For each story, we fed all the chunks of text corresponding to that TR and all prior TRs into the LLM, up to a maximum context size of 512 tokens. For last token pooling, we selected the representation of the last token in the current chunk of text. For mean or sum pooling, we took the mean, or summed, across tokens within the current chunk of text.

### Orthogonal autocorrelated sequences model (*OASM*)

In general, brain responses nearby in time are more similar than brain responses further away in time. To model this temporal autocorrelation in brain responses, we construct a feature space for each dataset by (1) forming an *n*-dimensional identity matrix, where *n* is the total number of “stimuli" in the dataset for which distinct neural data were recorded (i.e., the number of rows in the original data matrix), and then (2) applying a Gaussian filter within blocks along the diagonal that correspond to temporally contiguous time points (i.e., within each passage in *Pereira2018*, each sentence in *Fedorenko2016*, and each story in *Blank2014*). This generates an autocorrelated sequence for each passage/sentence/story that is orthogonal to that of each other passage/sentence/story. Because of this orthogonality, OASM is by construction incapable of predicting brain responses when using contiguous splits.

We use the *scipygaussian_filter1d* Virtanen et al.^39^ function to implement the Gaussian filter, and search through 48 sigma values (evenly spaced from 0.1 to 4.8) to find the optimal sigma value. We searched through 48 sigma values because *GPT2XL* has 48 layers, and so this allows for a fair comparison between the neural predictivity of both models.

It is important to clarify that although *OASM* is designed to model temporal autocorrelation, the neural variance it predicts is likely at least partly linguistically driven. This is because stimuli within a passage/sentence/story are also generally more linguistically similar than stimuli from different passages/sentences/stories. However, the key point is that in these datasets it is not possible to disentangle neural variance related to temporal autocorrelation from such linguistically driven neural variance and, furthermore, the representations of *OASM* yield essentially no theoretically interesting insight into human language processing.

### Positional signals and word rate

Here we describe the construction of the positional signal and word rate (*PWR*) for each neural dataset.

#### Pereira2018

We originally constructed the positional model as a one-hot 4*D* vector, where each element corresponds to a given position of a sentence in the passage. We then applied a Gaussian filter to this one-hot vector in order to render activations for nearby positions more similar to one another. For this filter, we selected the *σ* value that maximized neural predictivity, searching through 48 (same number of layers as *GPT2XL*) *σ* values evenly spaced from 0 to 4.7. The word rate model was a 1*D* scalar which quantifies the number of words within each sentence.

#### Fedorenko2016

The primary finding in the paper which first reported this neural dataset, Fedorenko et al.^16^, was that neural activity exhibited ramping behavior across the words within each sentence. We thus first created a 1*D* positional signal whose activation increases across word positions within a sentence. We concatenated onto this 1*D* positional signal an 8*D* (because there were 8 words per sentence) one-hot positional vector to also model non-ramping neural activity. Because we expect positional representations in the brain to be more similar between adjacent words than between more distant words, we applied a Gaussian filter to the 8*D* positional signal. We searched across 48 *σ* values, evenly spaced from 0 to 4.7, and selected the *σ* value that maximized neural predictivity.

#### Blank2014

The positional model was a ramping signal which exhibited ramping behavior up to *N* TRs, and then displayed constant activation. For instance, when *N* = 4 the ramping signal was a vector of the following form: [0, 1, 2, 3, 3, …, 3]. For a given *N*, we also stacked on all ramping signals for smaller values of *N*, up to a minimum of *N* = 3. We selected the value of *N* which results in the highest test-set neural predictivity, and searched through values of *N* from 3 (corresponding to a ramping signal) to 51 (corresponding to a 102. The word rate model was a 1*D* scalar which quantified the number of words in the chunk of text corresponding to a given TR.

### Next-word prediction metric

For our next-word prediction analyses, we use the exact same perplexity scores reported by Schrimpf et al.^2^. In that work, models were evaluated on the WikiText-2 corpus (2M / 218k / 246k tokens in the train / validation / test splits)^40^. Each model tokenized the text with its native tokenizer (vocabulary capped at 250k) and processed it in contiguous 32-token blocks. For every block the final-layer hidden state (penultimate, if the model contained a classifier head) was passed to a single linear read-out trained to predict the next token among the 50k most frequent vocabulary items using cross-entropy loss, AdamW optimization (learning rate = 5 × 10^−5^, mini batches = 4 blocks) and early stopping on validation loss; only the linear read-out’s weights were updated. Predictive performance was quantified as perplexity on the held-out test set. We do not recompute these scores: all perplexity values used here are taken directly from Schrimpf et al.^2^ so that our analyses are anchored to the identical scores originally linked to neural predictivity.

### Syntax

Syntactic embeddings (*SYNTAX*) are derived by averaging together the embeddings of sentences that all share the same syntactic structure but differ in their content words. This procedure averages away differences in word/phrase/sentence-level meaning between the sentences, and keeps what is shared between them: syntactic structure (and abstract semantics associated with that structure). First, we create a word bank for each part-of-speech and dependency tag in the original sentences by passing 300, 000 sentences from the Generics KB corpus^41^ through the SpaCy transformer-based model^42^. Then, we run each sentence in *Pereira2018* through the SpaCy transformer-based model. We then generate new sentences, in which each content word from an original sentence is replaced with a content word of matching part-of-speech and dependency tag randomly sampled from the word bank. Each newly generated sentence is then once again passed through the SpaCy transformer-based model to get the token indices of the subtrees associated with each token; we ensure that, for each token index, the token indices of the subtree in the generated sentence match those in the original sentence. If not, we discard the generated sentence and try again. We continue until 170 new sentences with valid subtrees are generated for each original sentence. Finally, we take the cross-entropy loss of *GPT2XL* on each newly generated sentence and keep the 100 sentences with the smallest loss. To get a syntactic representation for each original sentence, we run each of these 100 new sentences through *GPT2XL* and average their representations. This method is highly similar to that of^20^, the main difference being that we keep function words from the original sentence. We keep function words for two reasons: (i) representations of function words have previously been used to generate representations of syntactic form for neural encoding^12^, and (ii) coherent sentences were generated much more often when function words were left intact. Altogether, our syntactic representations combine the constraints on part-of-speech and dependency structure, as in Caucheteux et al.^20^, and the preservation only of function words, as in Kauf et al.^12^. We used sum pooling to extract activations for *SYNTAX* because it resulted in higher neural predictivity than mean pooling or using the last token.

### Regression methods

We used three linear regression methods throughout our paper. For all regression methods, we predict brain responses for each voxel/electrode/fROI independently. To compare our findings with Schrimpf et al.^2^, we employed ordinary least squares (OLS), which we implemented ourselves in order to run it on GPU. In our setting where the number of features often exceeds the number of samples, the OLS estimator is computed via the Moore-Penrose pseudoinverse and yields the unique minimum-*ℓ*_2_-norm solution among all least-squares minimizers; equivalently, it is the limit of ridge regression as the L2 penalty *λ* → 0^+^. When *X* has full row rank, this solution can exactly interpolate the training data, but it is typically high-variance and ill-conditioned in practice—hence we restricted OLS to replication of Schrimpf et al.^2^. We instead used L2-regularized (ridge) regression for the majority of our analyses, which is the standard choice in the majority of neural encoding studies. When fitting regressions with both small and large feature spaces, we employed banded ridge regression to give small feature spaces their own L2 penalty^43^. This is because standard ridge regression assigns a single L2 penalty for all feature spaces, which can bias the regression against making use of small feature spaces (see Supplementary for an expanded description of banded ridge regression). We fit all L2-regularized regression models with the *himalaya* package from Tour et al.^43^. Within each outer fold, we z-score all predictors across samples in the training set before fitting regression models, and we use the same training set statistics to normalize predictors in the validation and test sets.

### Evaluation metrics

We define an encoding model (or simply model), *M*, as a set of feature spaces. To quantify the neural encoding performance of a model on the test set, we use both Pearson *r* and the out of sample *R*^2^ metric (${R}_{oos}^{2}$)^44^. ${R}_{oos}^{2}$ quantifies how much better a set of features performs at predicting held-out neural data compared to a model which simply predicts the mean of the training neural data (i.e., a regression with only an intercept term). Given predictions, $\widehat{y}$, from a model *M* and predictions from a model with only an intercept term, denoted as ${\hat{y}}_{train}$, then:

1 $${R}_{oos}^{2}=1-\frac{{\sum }_{i}{({y}_{i}-{\hat{y}}_{i})}^{2}}{{\sum }_{i}{({y}_{i}-{\hat{y}}_{train})}^{2}}.$$

A positive (negative) value indicates that *M* was more (less) helpful than predicting the mean of training data. We elected to use ${R}_{oos}^{2}$ over the standard *R*^2^ because of this clear interpretation and because it is a less biased estimate of test set performance^44^. For variance partitioning analyses, we exclusively use ${R}_{oos}^{2}$ and not Pearson *r* because ${R}_{oos}^{2}$ can be interpreted as the fraction of variance explained. For both metrics, we take the mean neural predictivity across voxels/electrodes/fROIs for each participant, clip these within-participant mean values to be at least 0 to prevent noisy participants from biasing the mean downwards, and then compute the mean value across participants.

### Train, validation, and test folds

For our main analyses, we constructed contiguous splits by ensuring brain responses from the same passage/sentence/story are not included in both train and test data. Due to low sample sizes, we employed a nested cross-validation procedure for each dataset. In the outer cross-validation loop, the brain responses were divided into training and testing sets. In the inner cross-validation loop, the training data was divided into training and validation sets to tune the L2 penalty values when using L2-regularized regression. To define the range of L2 penalty values, we generated a list of integers from  − 5 to 19, passed this list through numpy’s exp2 function Harris et al.^45^, and then concatenated on 0. We describe the construction of our nested cross-validation procedure for each neural dataset below.

#### Pereira2018

During each outer fold, a single passage from each of the 24 semantic categories from one experiment was selected, and half of these passages were designated as the test set. By selecting at most a single passage from each semantic category, we ensured there were several passages in the training set which belonged to the same semantic category as the passages in the testing set. This equated to 8 test folds for experiment 1 (4 passages per semantic category, half selected for each loop) and 6 test folds for experiment 2 (3 passages per semantic category). During each inner fold, we again selected one passage from each semantic category, and half of these passages were designated as validation (leading to 7 inner folds for experiment 1, and 5 inner folds for experiment 2).

#### Fedorenko2016

For each outer fold, we selected 4 out of the 52 sentences as the test fold, resulting in 13 outer folds. For each inner fold, we once again selected 4 sentences as the validation set, resulting in 12 inner folds per outer fold.

#### Blank2014

For each outer fold, we selected a single story as the test fold, resulting in 8 outer folds. For each inner fold, each of the remaining stories served in turn as the validation set, resulting in 7 inner folds.

Because several previous studies using these datasets^2,7–9,11–13^ employ shuffled splits, we also perform some analyses with shuffled splits. When using shuffled splits, data is arbitrarily placed into training and testing sets, such that data from the same passage/sentence/story can be in both the training and testing sets. To provide an example, this means that with the *Pereira2018* dataset it is possible for the first and third sentences within a passage to be in the training set, and the second sentence within the same passage to be in the testing set. In order to construct shuffled splits, we randomly shuffled the labels for each dataset that indicated which sentence belonged to which passage in *Pereira2018*, which word belonged to which sentence in *Fedorenko2016*, and which chunk of text corresponded to which story in *Blank2014*. We used numpy to perform this shuffling, and set the random seed to 42. Randomly shuffling the data labels made it so the testing and validation sets were no longer contiguous blocks of data, while still ensuring that the testing and validation sets were of the same size as the contiguous splits. We performed nested cross-validation in the exact same manner as described above when using shuffled splits.

When computing *R*^2^ across either inner folds or outer folds, we pooled predictions across folds and computed a single *R*^2^ as recommended by Hawinkel et al.^44^. When computing Pearson *r*, we computed the correlation for each fold and then averaged across folds, as done in Schrimpf et al.^2^.

### Selection of best layer

For each metric, we chose the layer that performs best across voxels/electrodes/fROIs on the test set with that metric. We used the test set to select the best layer to maintain consistency with Schrimpf et al.^2^. For *Pereira2018*, we selected the best-performing layer for *EXP2* and *EXP3* separately. Due to the stochastic nature of untrained LLMs, we selected the best layer for 5 random seeds and report the average performance across seeds. When reporting the best layer, we refer to layer 0 as the input static layer, and layer 1 as the first intermediate layer. We selected hyperparameters for *OASM* and *PWR* for each metric separately, as well.

### Deviations from Schrimpf et al

We make several methodological deviations from Schrimpf et al.^2^ as noted throughout the paper. Here, we list them explicitly.

1. We use a larger number of folds for each dataset (8 for EXP1 in *Pereira2018*, 6 for EXP2 in *Pereira2018*, 13 for *Fedorenko2016*, and 8 for *Blank2014*). On the other hand, Schrimpf et al.^2^ uses 5-fold cross-validation for all datasets.
2. We use L2-regularized (ridge) regression instead of ordinary least squares regression (except where noted in Fig. 1).
3. We use three feature extraction methods: last token, mean-pooling, and sum-pooling. We report the results for each, and emphasize the best performing method for each model. By contrast Schrimpf et al.^2^ only used last token pooling.
4. We use the RWKV-4 series of models as representative RNN language models. By contrast, Schrimpf et al.^2^ used two RNN-based models: *Skip-Thoughts* and *LSTM-LM1B*.
5. In the *Pereira2018* analyses, Schrimpf et al.^2^ accidentally included sentences from the same passage as well as category as context when inputting a given sentence into a language model. We correct for this, and only include the preceding sentences from a given passage as context.

### Percentage of LLM neural predictivity accounted for

Given a set of models *M*, we quantify the percentage of neural variance explained by an LLM that is accounted by *M* for a given participant through Eq. (3).

2 $${R}_{M+LLM}^{2}\ast=\max({R}_{M+LLM}^{2},{R}_{LLM}^{2})$$

3 $${\Omega }_{LLM}(M)=\left[1-\min\left(\frac{{R}_{M+LLM}^{2}\ast -{R}_{M}^{2}}{{R}_{LLM}^{2}},0\right)\right]\times 100{\rm{\%}}$$

Here, *R*^2^ here denotes the mean *R*^2^ across voxels/electrodes/fROIs in a given participant. For a given participant, we first ensured that the neural predictivity of the *M+LLM* model was not lower than the predictivity of the *LLM* alone. Lower predictivity of *M+LLM* than *LLM* may happen due to overfitting, and in such cases we would unfairly state that *M* accounts for more of the neural predictivity of the *LLM* than it actually does. We denote this corrected neural predictivity as ${R}_{M+LLM}^{2}*$ (Equation (2)). The numerator, ${R}_{M+LLM}^{2}*-{R}_{M}^{2}$, quantifies the additional neural variance an *LLM* explains over *M* alone (this is the variance explained by the LLM that is not explained by M). It is possible for this quantity to be less than 0 because adding the *LLM* to *M* may hurt neural predictivity (i.e., ${R}_{M+LLM}^{2}* < {R}_{M}^{2}$). We then normalize this additional variance by the variance the *LLM* explains alone, ${R}_{LLM}^{2}$, and clip negative values since they are not interpretable. We subtract this quantity from 1 and multiply it by 100 to obtain the percentage of neural variance that the LLM explains that is also explained by *M*. We compute Ω for each participant separately, and a higher Ω value indicates that *M* accounts for more of the neural predictivity of the LLM.

### Statistical testing

We perform two statistical tests within this study. When comparing the neural predictivity of an *LLM* to that of another model, we perform a one-side Wilcoxon signed-rank test across participants to determine whether the neural predictivity of the *LLM* is higher. The sample size for this test was *N* = 10 for *Pereira2018*, and *N* = 5 for *Fedorenko2016* and *Blank2014*. We exclusively perform this test on Pearson *r* values throughout the study.

We also perform a second statistical test to determine whether a regression that stacks together activations from a set of models, *M*, and from an *LLM* (*M+LLM*), explains significantly more neural variance than *M* alone at the voxel/electrode/fROI level. When performing this test, we first selected the best sub-model within *M+LLM*, denoted as M+LLM*, and the best sub-model within *M*, denoted as M*, for each voxel/electrode/fROI separately. For instance, if comparing *PWR+GloVe+GPT2XL* to *PWR+GloVe*, we selected the sub-model which achieved the highest neural predictivity for each voxel/electrode/fROI for *PWR+GloVe+GPT2XL* and *PWR+GloVe*, separately. We then computed the squared error values on the test data for each of these models separately, which are obtained by subtracting the predictions of the regression model from the target brain responses and squaring this difference. We passed the squared error values from both models into a one-sided paired t-test which evaluates whether the squared error values from M+LLM* are significantly less than those of M*. The p-values across voxels/electrodes/fROIs were then FDR corrected Benjamini and Hochberg^46^ within each participant, and an *α* value of 0.05 is used to determine significance. The sample size for this t-test was *N* = 384 (where *N* refers to the number of samples) for voxels in *Pereira2018* with only data from *EXP2*, *N* = 243 for voxels in *Pereira2018* with only data in *EXP3*, and *N* = 627 for voxels with data in both experiments. For *Fedorenko2016*, the sample size was *N* = 416, and for *Blank2014* the sample size was *N* = 1317.

While squared error values are not always normally distributed, our sample sizes were large: (the minimum sample size was 243), so we still opted to use a t-test over a non-parametric alternative Lumley et al.^47^. We note that squared error values from a model are correlated across consecutive samples, which means that the t-test is biased towards positive results which show that an LLM contributes significant variance over a set of simpler models i.e., the t-test has an inflated Type 1 error). While this bias is not ideal, it is conservative in our case, making it harder to account for the neural variance that an LLM explains with simpler models.

To ensure findings using the paired t-test were robust, we replicated the analyses relating to *GPT2XL* and *GPT2XLU* using a blocked paired t-test. To perform the blocked paired t-test, we averaged squared error values within each passage for *Pereira2018*, each sentence for *Fedorenko2016*, and within non-overlapping 10 TR (20 sec) segments *Blank2014* (Supplementary Fig. 7). Averaging mitigates non-independence since squared error values are most correlated within a given passage, sentence, or consecutive TRs. Sample sizes after averaging were still sufficiently large for the normality assumption: *N* = 96 for voxels in *Pereira2018* with only data from *EXP2*, *N* = 72 for voxels in *Pereira2018* with only data in *EXP3*, *N* = 168 for voxels with data in both experiments, *N* = 52 for *Fedorenko2016*, and *N* = 138 for *Blank2014*. Given that normality assumptions were met, we elected to use the a blocked t-test rather than a blocked permutation test.

### Preprocessing details

We use all preprocessed datasets as provided by Schrimpf et al.^2^. We summarize their preprocessing pipelines below.

#### *Pereira2018* (fMRI)

Data were preprocessed in SPM5 (default settings unless noted): motion correction (realignment to the mean of the first functional run, 2nd-degree B-spline interpolation), normalization (estimated on the mean image with trilinear interpolation), resampling to 2 mm isotropic voxels, spatial smoothing with a 4 mm FWHM Gaussian kernel, and high-pass filtering with a 200-s cutoff. A standard mass-univariate GLM was fit in SPM5 to estimate the response to each sentence in each run using a boxcar regressor convolved with the canonical HRF; first-order temporal derivatives were included (but not used downstream) along with nuisance regressors for runs and offline motion parameters. Functional localization targeted the bilateral fronto-temporal language network using the sentences > nonwords localizer (Fedorenko et al., 2010) with group-constrained, participant-specific masks covering six regions per hemisphere (IFGorb, IFG, MFG, AntTemp, PostTemp, AngG); within each mask the top 10% most localizer-responsive voxels (highest t-values) were selected. Stimulus-response matrices were constructed by averaging the sentence-level responses across the three repetitions, yielding one value per sentence per language-responsive voxel.

#### *Fedorenko2016* (ECoG)

Signals (*n *= 5 participants) were digitized at 1,200 Hz. Preprocessing followed Fedorenko et al.^16^ with noted deviations: (i) high-pass filter at 0.5 Hz; (ii) removal of reference, ground, and high-noise electrodes; (iii) common-average reference applied within amplifiers for electrodes below a preset line-noise threshold; (iv) notch filters removed 60 Hz line noise and harmonics. High-gamma activity was extracted via a Gaussian filter bank centered at 73, 79.5, 87.8, 96.9, 107, 118.1, 130.4, and 144 Hz (SDs 4.68-6.62 Hz); the analytic signal was obtained with the Hilbert transform, the eight band envelopes were averaged to form the high-gamma trace, z-scored per electrode across the session, and downsampled to 300 Hz (Schrimpf et al.^2^ did not additionally low-pass at 100 Hz, unlike in the original study Fedorenko et al.^16^). Language-responsive electrodes were defined via the same sentences > nonwords contrast as the fMRI localizer (p < . 01, per participant). For the core 52 sentences (8 words each), responses were averaged over each word’s full presentation window, producing a 416  × 97 (words  × electrodes) matrix across the five participants.

#### Blank2014 (fMRI)

Five participants listened to 8 narrated stories from the Natural Stories Corpus (TR = 2 s; 1,317 TRs total). Per Blank et al.^17^, signals were filtered between 0.008 Hz and 0.09 Hz, thereby removing slow drifts and high frequency noise. Analyses were restricted to language-responsive voxels defined with the same sentences > nonwords localizer and group-constrained, participant-specific masks. For alignment, story audio was segmented into consecutive 2-s intervals matched to TRs (words straddling boundaries were assigned to the interval in which they ended). Given the HRF delay, each BOLD time point was assumed to reflect text that occurred 4 s (2 TRs) earlier. We also include a supplementary analysis using model representations from the previous 4 TRs concatenated together, as in Huth et al.^18^, in Supplementary Fig. 8. For each participant, BOLD signals were averaged within each language fROI to improve SNR, yielding 12 fROIs per participant (6 per hemisphere; 60 total). Concatenating all story fragments produced a 1,317  × 60 (TRs  × fROIs) matrix.

### Computational resources

Figures 1, 2,5, and 6 were generated using an Ubuntu server with three Nvidia GeForce RTX 3090 GPUs and an AMD Ryzen Threadripper 3960X 24-Core CPU with 125 GiB of memory. Only one GPU was used for any given analysis. Figures 3, 4 were generated using a single Nvidia GeForce RTX 4090 GPU and an AMD 7950X 16-Core CPU.

### Reporting summary

Further information on research design is available in the Nature Portfolio Reporting Summary linked to this article.

## Supplementary information

Supplementary Information

Reporting Summary

Transparent Peer Review file

## Data Availability

The neural data, as well as associated texts, for the three datasets have been deposited at the Figshare database at the following link: Figshare. Source data for the manuscript is provided at the following link: Source Data.

## Code availability

Code for this manuscript is available at the following link: Code.
