# Supplementary material for: Spurious alignment between large language models and brains can emerge from non-robust methods and overlooked confounds
Source: Nat Commun. 2026 Apr 27;17:5769. doi: 10.1038/s41467-026-72253-7 (PMC13324000; doi:10.1038/s41467-026-72253-7)
Supplement: Supplementary file 1 — Supplementary Information [file 41467_2026_72253_MOESM1_ESM.pdf]

1 Supplementary Discussion

Hosseini et al. [2024] noted that although *GPT2XL* performed particularly well on *Pereira2018*, most contextual models also performed fairly well relative to a "noise ceiling" estimated via inter-subject predictivity. This led them to hypothesize that "universal representations" — those consistent across models — underlie the mapping between large language models (LLMs) and brain activity. To test this hypothesis, the authors constructed a new neural dataset specifically comprising "low agreement" sentences, whose representations varied significantly across models. They found that the ratio between LLM neural predictivity and the noise ceiling was much lower on this dataset than on *Pereira2018*, which they interpreted in support of their hypothesis.

However, this discrepancy between the two datasets can be attributed to a simpler explanation. In *Pereira2018*, the noise ceiling is inflated in the same manner as LLM neural predictivity due to the "OASM-like" structure of brain responses, where brain responses are more similar within a passage than across distinct passages. The "low agreement" dataset, on the other hand, consisted of isolated, unrelated sentences presented one at a time in a consistent order across participants in several runs. Because previous sentences were not provided as context and temporally adjacent sentences were not similar, model activations no longer exhibited "OASM-like" properties like they did in *Pereira2018*, reducing the artificial inflation of neural predictivity scores. However, since brain responses remained temporally autocorrelated due to the fixed presentation order, the noise ceiling remained inflated when using shuffled test splits. Therefore, the observed differences in neural predictivity relative to the noise ceiling between *Pereira2018* and the "low agreement" dataset are likely a consequence of shuffled test splits and dataset design choices.

While perplexity is a standard metric for evaluating unidirectional language models, it is not necessarily well-suited for bidirectional models. By design, bidirectional architectures such as *BERT* are not trained to perform next-word prediction, but rather to fill in masked tokens using both left and right context. As a result, applying perplexity—which requires autoregressive prediction—places these models in a setting misaligned with their training objective. Consequently, perplexity may underestimate the representational quality of bidirectional models and should be interpreted with caution when comparing them directly to unidirectional models.

Beyond just using contiguous splits, there are several ways analyses can be made more stringent. First, one can evaluate on entirely held-out stimuli (e.g., whole stories never seen in any train or validation fold), as in Huth et al. [2016]. Second, one can enforce strictly non-overlapping vocabularies between train and test (e.g., Goldstein et al. [2024]), which is especially stringent for word-level/ECoG settings and helps diagnose lexical memorization versus generalization. Third, resetting model context windows at cross-validation boundaries (e.g., de Varda et al. [2025]) prevents inadvertent context leakage when folds split within long stimuli. Finally, cross-subject generalization (e.g., Zada et al. [2024]) tests whether mappings learned on some individuals transfer to unseen individuals, though it introduces variability from inter-subject alignment. We view temporally contiguous cross-validation as a baseline, and these stricter regimes as complementary stress tests whose adoption should be matched to the stimulus structure and analysis goals. If one insists on using shuffled splits, one can insert temporal buffer zones (e.g., $\pm 20\,\mathrm{s}$) around train–test boundaries to reduce autocorrelation leakage; however, this approach discards substantial amounts of already limited data and is generally inferior to simply using temporally contiguous splits.

2 Functional Localization

For *Pereira2018* and *Blank2014*, the language network was defined by the following procedure Fedorenko et al. [2011]. First, voxels were identified in each participant which showed stronger responses to sentences compared to lists of non-words (sentences > non-word lists contrast). These voxels were then constrained by data-driven language activation maps formed by applying the same contrast to many other participants. Finally, the top 10% of the voxels were selected which showed the greatest sentences > non-word lists difference. For *Blank2014*, voxel responses were averaged with each region of interest (ROI) for each participant to improve the signal-to-noise ratio. On *Fedorenko2016*, language-responsive electrodes were selected where the z-scored envelope of the gamma

activity was significantly higher during the sentences than a condition where participants read nonword lists. For a more complete description of the functional localization procedures see Schrimpf et al. [2021].

3 Banded Ridge Regression

We used a random search method to optimize the banded regression hyperparameters Dupré la Tour et al. [2022]. Banded regression has two hyperparameters, γ, which is a vector of shape number of models determines how much each model is scaled, and α, which is the L2 penalty applied across all models. Values for γ are drawn from a Dirichlet distribution and hence sum to 1. Down-scaling a certain feature space relative to others is functionally equivalent to assigning a separate L2 penalty for each feature space. This is because when a feature space is down-scaled, the L2 magnitude of the weights must increase for it to have a meaningful contribution to the predictions, which equates to increasing the L2 penalty for that feature space. The optimal γ and α combination were found for each voxel/electrode/fROI by performing a random search over γ values, storing the α value that performed best for that γ value on validation data, and then selecting the best performing γ and α combination. Before starting the random search, we tried all combinations of γ values that removed a model (i.e. down-scaled at least one feature space to 0) to ensure the regression had an opportunity to remove features which hurt performance. Importantly, because γ values were determined based on validation performance, it is possible for a model to be kept despite ultimately hurting neural predictivity on the test set. We ran banded ridge regression for a maximum of 1000 random search iterations with early stopping if the mean neural predictivity did not improve by more than 10^{-4} after 50 iterations. When fitting regressions with positional signals and word rate, we treated them as one model (i.e. a single γ was applied).

4 Comprehensive review of methods used in previous studies

Below, we include a large sample of studies in the LLM-to-brain mapping space. For each study, we list (a) the style of train-test split used, (b) which confounds were controlled for and how they were controlled for, (c) the activation extraction method used, and (d) the dataset type.

Supplementary Table 1: Methodological variations across studies

Paper	Split style	Low-level Confounds accounted for	Feature extraction method	Dataset type (fMRI, ECoG, MEG)
**AlKhamissi et al. (2024)** – *"Brain-Like language processing via a shallow untrained multihead attention network"*	Shuffled	Position is accounted for in a supplementary analysis (in an attempt to refute an earlier version of our paper), but appears far worse than GPT2-XL due to the authors' use of shuffled splits, which inflates GPT2-XL but not position (due to position vectors being identical between passages and hence lacking the "orthogonal" property of OASM)	Last token and mean pooling	fMRI and ECoG
**Antonello et al. (2021)** – *"Low-dimensional structure in the Space of Language Representations is Reflected in Brain Responses"*	Contiguous, a single held-out story was used for testing.	None	Sum-pooling with Lanczos downsampling	fMRI

Paper	Split style	Low-level Confounds accounted for	Activation extraction method	Dataset type (fMRI, ECoG, MEG)
**Antonello et al. (2023)** – *"Scaling laws for language encoding models in fMRI"*	Contiguous, a single held-out story was used for testing.	None included in the regression, but the first 100 seconds of the test set were removed to avoid ramping positional confounds observed in the signal.	Sum-pooling with Lanczos downsampling	fMRI
**Antonello & Huth (2024)** – *"Predictive coding or just feature discovery?"*	Contiguous, a single held-out story was used for testing.	None	Sum-pooling with Lanczos downsampling	fMRI
**Aw et al. (2024)** – *"Instruction-tuning aligns LLMs to the human brain"*	Shuffled	None	Last token	fMRI
**Caucheteux et al. (2021)** – *"Disentangling syntax and semantics in the brain with deep networks"*	Contiguous	Word rate, phonemes, stress and tone	Sum-pooling with Lanczos downsampling	fMRI
**Caucheteux et al. (2021)** – *"Model-based analysis reveals the hierarchy of language in 305 subjects"*	Contiguous	Phonemes (as represented in intermediate layers of GPT2)	Sum-pooling within TRs and concatenation of the resulting vectors from the last 5 TRs.	fMRI
**Caucheteux et al. (2022)** – *"Deep language algorithms predict semantic comprehension from brain activity"*	Contiguous, time series split into 5 contiguous chunks	Word rate, phone rate, stresses and tones of words of the stimuli	Sum-pooling within TRs and concatenation of the resulting vectors from the last 5 TRs.	fMRI
**Caucheteux & King (2022)** – *"Brains and algorithms partially converge in natural language processing"*	Contiguous, chunks of 5 consecutive sentences were split between train and test sets	Visual embeddings from a CNN trained to recognize words from images	Sum-pooling within TRs and concatenation of the resulting vectors from the last 5 TRs.	fMRI and MEG
**Caucheteux et al. (2023)** – *"Evidence of a predictive coding hierarchy in the human brain listening to speech"*	Contiguous, time series split into 5 contiguous chunks	None	Sum-pooling within TRs and concatenation of the resulting vectors from the last 5 TRs.	fMRI
**Deniz et al. (2023)** – *"Semantic Representations during Language Comprehension Are Affected by Context"*	Contiguous, a single held-out story was used for testing.	Word rate, number of letters, letters, word length variation per TR	Finite impulse response model with four delays (not specified whether done with mean-pooling, sum-pooling, or sum-pooling with Lanczos downsampling (i.e. delta-sum)	fMRI
**Goldstein et al. (2024)** – *"Alignment of brain embeddings and artificial contextual embeddings in natural language points to common geometric patterns"*	Contiguous and only using unique words	75 binary features for every word within the text, including part-of-speech, stop word, word shape with 16 features, types of prefixes with 19 dimensions, and types of suffixes with 28 dimensions. No positional confounds accounted for, but the style of train-test split (using contiguous chunks *within* the same story, with LLM context reset at the beginning of each fold) makes it unlikely that the LLM could've exploited any positional effects.	Not specified, but likely not so important since the dataset is ECoG	ECoG

Paper	Split style	Low-level Confounds accounted for	Activation extraction method	Dataset type (fMRI, ECoG, MEG)
**Goldstein et al. (2025)** – *"A unified acoustic-to-speech-to-language embedding space captures the neural basis of natural language processing in everyday conversations"*	Contiguous	"Acoustics" and "speech" (extracted from 0th and final layer in Whisper encoder)	Not specified whether tokens within the same word are averaged or if only the last is used, but likely not so important since the dataset is ECoG.	ECoG
**Goldstein et al. (2022)** – *"Shared computational principles for language processing in humans and deep language models"*	The text indicates that the data was split "randomly", but the authors have clarified to us that they did in fact use contiguous splits but reported incorrectly	Word-level embeddings (GloVe) and arbitrary embeddings	Uses the last layer's representation of the preceding token to represent each word	ECoG
**Hong et al. (2024)** – *"Scale matters: large language models with billions (rather than millions) of parameters better match neural representations of natural language*	Contiguous, 10 fold cross-validation	None	Not specified whether tokens within the same word are averaged or if only the last is used, but likely not so important since the dataset is ECoG.	ECoG
**Hosseini et al. (2024a)** – *"ANN language models predict human brain responses… after a developmentally realistic amount of training"*	Shuffled	None	Last token	fMRI
**Hosseini et al. (2024b)** – *"Universality of representation in biological and artificial neural networks"*	Shuffled	None	Last token	fMRI
**Huth et al. (2016)** – *"Natural speech reveals the semantic maps that tile human cortex" (Nature 2016)*	Contiguous, a single held-out story was used for testing	Phonemes, phoneme rate, and word rate	Sum-pooling with Lanczos downsampling	fMRI
**Jain et al. (2018)** – *"Incorporating context into language encoding models for fMRI"*	Contiguous, a single held-out story was used for testing	None	Sum-pooling with Lanczos downsampling	fMRI
**Jain et al. (2020)** – *"Interpretable multi-timescale models for predicting fMRI responses to continuous natural speech"*	Contiguous, a single held-out story was used for testing	None	Sum-pooling with Lanczos downsampling. Also introduces RBF interpolation (which extracts model dimensions with high temporal integration timescales in a word-rate-independent manner, more analogous to mean-pooling).	fMRI
**Kauf et al. (2023)** – *"Lexical-semantic content, not syntactic structure, drives ANN–brain similarity"*	Shuffled	Analyses investigate predictivity of LLM embeddings when scrambling word order or removing content words, but no analyses are done to investigate the role of low-level confounds such as word rate or position	Last token and mean-pooling	fMRI

**Paper**	**Split style**	**Low-level Confounds accounted for**	**Activation extraction method**	**Dataset type (fMRI, ECoG, MEG)**
**Kumar et al. (2024)** – *"Shared functional specialization in transformer LMs and the human brain"*	Contiguous	Phonemes, phoneme rate, word rate, and a silence indicator included in all regressions. Linguistic features including part-of-speech and syntactic dependencies were used as a baseline model.	Mean-pooling of LLM representations within each TR; concatenation of the last 5 TRs	fMRI
**LeBel et al. (2021)** – *"Voxelwise encoding models show that cerebellar language representations are highly conceptual"*	Contiguous, a single held-out story was used for testing	Articulatory, spectral, and word-level semantic features as well as part of speech.	Sum-pooling with Lanczos downsampling	fMRI
**Mischler et al. (2024)** – *"Contextual feature extraction hierarchies converge in LLMs and the brain"*	Shuffled	None	Last token	iEEG
**Oota et al. (2022)** – *"Neural language taskonomy: which NLP tasks most predict fMRI brain activity?"*	Shuffled	None	Mean-pooling per-sentence (for analyses on the Pereria2018 dataset); mean-pooling within each TR (for the Narratives-Pieman dataset)	fMRI
**Pasquiou et al. (2022)** – *"Neural Language Models are not Born Equal to Fit Brain Data, but Training Helps"*	Contiguous	None	Convolution of token-level activations with the canonical HRF (analogous to sum-pooling since post-convolution activation scales in magnitude with word rate)	fMRI
**Pasquiou et al. (2023)** – *"Information-Restricted Neural Language Models Reveal Different Brain Regions' Sensitivity to Semantics, Syntax and Content"*	Contiguous	Acoustic energy, word-rate, and log of the unigram lexical frequency of each word.	Convolution of token-level activations with the canonical HRF (analogous to sum-pooling since post-convolution activation scales in magnitude with word rate)	fMRI
**Reddy and Wehbe (2021)** – *"Can fMRI reveal the representation of syntactic structure in the brain"*	Contiguous	Punctuation, complexity metrics, dependency tags, constituency tree-based graph embeddings	Sum-pooling within TRs, concatenation of the last 4 TRs.	fMRI
**Schrimpf et al. (2021)** – *"The neural architecture of language: converges on predictive processing"*	Shuffled	None	Last token	fMRI and ECoG
**Toneva et al. (2019)** – *"Interpreting and improving natural-language processing (in machines) with natural language-processing (in the brain)"*	Contiguous (not specified in the paper, but apparent in the code)	Word-level embeddings	Mean-pooling within TRs, concatenation of the last 4 TRs	fMRI and MEG
**Toneva et al. (2022)** – *"Combining computational controls with natural text reveals aspects of meaning composition"*	Contiguous (not specified in the paper, but apparent in the code)	Word-level embeddings	Mean-pooling within TRs, with concatenation of the last 4 TRs for fMRI experiments. Mean-pooling of multi-token words for MEG experiments.	fMRI and MEG

Paper	Split style	Low-level Confounds accounted for	Activation extraction method	Dataset type (fMRI, ECoG, MEG)
**Wehbe et al. (2014)** – *"Aligning context-based statistical models of language with brain activity during reading"*	Contiguous	Word length	The RNN LM used here operates over full words, and the dataset is word-level MEG responses, so activation extraction is straightforward here and does not depend on choices like mean- or sum- pooling.	MEG
**Zada et al. (2024)** – *"A shared model-based linguistic space for transmitting thoughts from brain to brain in natural conversations"*	Contiguous	None	Mean-pooling; multi-token words are represented with the average of the LLM's representations for the composite tokens	ECoG
**Zada et al. (2025)** – *"The 'Podcast' ECoG dataset for modeling neural activity during natural language comprehension"*	Contiguous (not specified in the paper, but apparent in the code)	Word-level embeddings, syntactic features, phonetic features, and acoustic features	Not specified whether tokens within the same word are averaged or if only the last is used, but likely not so important since the dataset is ECoG.	ECoG
**Zada et al. (2025)** – *"Linguistic coupling between neural systems for speech production and comprehension during real-time dyadic conversations"*	Contiguous, evaluates "the performance of production and comprehension time points separately in a held-out test run of different conversations"	"Acoustic", "phonemic", and "linguistic" confounds. The only linguistic confound mentioned is word rate. They also include a boxcar "design structure" predictor in their preprocessing confound regression (which also includes head motion, physiological noise, and detrending). This "design structure" predictor is an on-off predictor that is on or off during speaker or listener turns, respectively.	Uses a method akin to mean pooling (takes the average token representation within each TR, also concatenates from 1-5 TRs back)	fMRI

5 Neural predictivity (Pearson r) when using validation set for layer selection

Model Name	Split Type	Selection w/ Validation	Selection w/ Test	Dataset
GPT2-XL	Shuffled	0.1540	0.1540	*Pereira2018*
OASM	Shuffled	0.2147	0.2157	*Pereira2018*
GPT2-XL	Contiguous	0.0215	0.0232	*Pereira2018*
Position	Contiguous	0.0092	0.0097	*Pereira2018*
GPT2-XL	Shuffled	0.0836	0.0855	*Fedorenko2016*
OASM	Shuffled	0.0969	0.0979	*Fedorenko2016*
GPT2-XL	Contiguous	0.0489	0.0509	*Fedorenko2016*
Position	Contiguous	0.0465	0.0471	*Fedorenko2016*
GPT2-XL	Shuffled	0.0017	0.0029	*Blank2014*
OASM	Shuffled	0.2747	0.2750	*Blank2014*
GPT2-XL	Contiguous	0.0001	0.0004	*Blank2014*
Position	Contiguous	0.0069	0.0085	*Blank2014*

Supplementary Table 2: Selection with validation and selection with test columns show R^2 values on the test set for the respective model, split type, and dataset. In the selection with validation column, the best layer/hyperparameter was selected using the validation set, and in the selection with test column the best layer/hyperparameter was selected using the test set (as done throughout the paper). Neural predictivity values are similar when using either the validation or test set for selection.

6 Examples of syntactically equivalent sentences used to generate the *SYNTAX* model

**Original Sentences**	**Syntactically Equivalent Sentences**
Beekeeping encourages the conservation of local habitats.	Gold refers the virus of public nests.<br>Gastrulation influences the protein of social cancers.<br>Steel helps the irritation of physical pesticides.
It is in every beekeeper's interest to conserve local plants that produce pollen.	It is in every person's art to categorize common surfaces that extend ability.<br>It is in every civilization's growth to cause pediatric opinions that stimulate nature.<br>It is in every victim's ground to abandon hot crystals that tap caffeine.
As a passive form of agriculture, it does not require that native vegetation be cleared to make way for crops.	As an ordered encephalomyelitis of impact, it does not collect that small loss be honored to keep language for crashes.<br>As a neat unemployment of culture, it does not cause that specific iron be performed to do plant for reptiles.<br>As a seminal appetite of acidity, it does not catch that social lacquer be passed to make mortality for fibroids.
Beekeepers also discourage the use of pesticides on crops, because they could kill the honeybees.	People rather avoid the damage of habitats on ecosystems, because they could grow the compounds.<br>Cockroaches also use the access of pipes on pores, because they could spend the deposits.<br>Species now save the water of grasses on groups, because they could play the stingers.

Supplementary Table 3: Original sentences from a passage in *Pereira2018*, as well as 3 sample syntactically equivalent sentences for each.

Split	AE	P × F		P × B		F × B	
		Incl. static	Contextual-only	Incl. static	Contextual-only	Incl. static	Contextual-only
Contiguous	Last	—	7.13×10^{-1}	—	7.64×10^{-1}	—	2.20×10^{-1}
Contiguous	Mean	$\mathbf{2.90 \times 10^{-14}}$	1.84×10^{-1}	$\mathbf{9.24 \times 10^{-7}}$	$\mathbf{3.19 \times 10^{-3}}$	$\mathbf{6.14 \times 10^{-5}}$	2.82×10^{-1}
Contiguous	Sum	$\mathbf{1.09 \times 10^{-11}}$	2.05×10^{-1}	$\mathbf{2.23 \times 10^{-4}}$	2.40×10^{-1}	$\mathbf{7.00 \times 10^{-5}}$	1.21×10^{-1}
Contiguous	Best	$\mathbf{8.09 \times 10^{-12}}$	3.08×10^{-1}	$\mathbf{1.22 \times 10^{-4}}$	3.11×10^{-1}	$\mathbf{1.81 \times 10^{-5}}$	1.24×10^{-1}
Shuffled	Last	—	$\mathbf{1.66 \times 10^{-5}}$	—	$\mathbf{1.42 \times 10^{-2}}$	—	$\mathbf{4.47 \times 10^{-4}}$
Shuffled	Mean	$\mathbf{4.33 \times 10^{-14}}$	$\mathbf{1.12 \times 10^{-13}}$	$\mathbf{7.23 \times 10^{-18}}$	$\mathbf{1.54 \times 10^{-15}}$	$\mathbf{2.03 \times 10^{-14}}$	$\mathbf{6.72 \times 10^{-13}}$
Shuffled	Sum	$\mathbf{2.46 \times 10^{-14}}$	$\mathbf{1.58 \times 10^{-15}}$	$\mathbf{1.54 \times 10^{-17}}$	$\mathbf{1.61 \times 10^{-15}}$	$\mathbf{6.47 \times 10^{-15}}$	$\mathbf{5.78 \times 10^{-14}}$
Shuffled	Best	$\mathbf{5.40 \times 10^{-9}}$	$\mathbf{1.48 \times 10^{-6}}$	$\mathbf{3.99 \times 10^{-8}}$	$\mathbf{3.84 \times 10^{-6}}$	$\mathbf{1.69 \times 10^{-14}}$	$\mathbf{5.02 \times 10^{-13}}$

Supplementary Table 4: P-values for pairwise correlations between datasets shown in Figure 3 split by activation extraction (AE) and whether static embedding models are included. Significant values ($p < 0.05$) are bolded.

Split	AE	Pereira		Fedorenko		Blank	
		Incl. static	Contextual-only	Incl. static	Contextual-only	Incl. static	Contextual-only
Contiguous	Last	—	8.46×10^{-2}	—	1.93×10^{-1}	—	6.73×10^{-1}
Contiguous	Mean	$\mathbf{1.12 \times 10^{-4}}$	8.26×10^{-1}	$\mathbf{9.37 \times 10^{-7}}$	3.63×10^{-1}	$\mathbf{6.63 \times 10^{-3}}$	5.51×10^{-1}
Contiguous	Sum	$\mathbf{7.98 \times 10^{-4}}$	9.13×10^{-1}	$\mathbf{1.44 \times 10^{-7}}$	1.11×10^{-1}	$\mathbf{1.72 \times 10^{-2}}$	6.69×10^{-1}
Contiguous	Best	$\mathbf{7.59 \times 10^{-4}}$	9.06×10^{-1}	$\mathbf{1.32 \times 10^{-7}}$	1.23×10^{-1}	$\mathbf{5.60 \times 10^{-3}}$	5.70×10^{-1}
Shuffled	Last	—	4.22×10^{-1}	—	$\mathbf{1.52 \times 10^{-3}}$	—	7.71×10^{-1}
Shuffled	Mean	$\mathbf{2.77 \times 10^{-4}}$	$\mathbf{1.51 \times 10^{-2}}$	$\mathbf{1.31 \times 10^{-7}}$	$\mathbf{2.02 \times 10^{-3}}$	$\mathbf{2.74 \times 10^{-4}}$	7.41×10^{-2}
Shuffled	Sum	$\mathbf{4.66 \times 10^{-4}}$	$\mathbf{1.04 \times 10^{-2}}$	$\mathbf{4.26 \times 10^{-7}}$	$\mathbf{7.72 \times 10^{-3}}$	$\mathbf{4.67 \times 10^{-4}}$	1.05×10^{-1}
Shuffled	Best	$\mathbf{1.54 \times 10^{-2}}$	3.68×10^{-1}	$\mathbf{2.00 \times 10^{-7}}$	$\mathbf{3.28 \times 10^{-3}}$	$\mathbf{2.74 \times 10^{-4}}$	7.41×10^{-2}

Supplementary Table 5: P-values for correlations with next-word prediction shown in Figure 4, split by activation extraction (AE) and whether static embedding models are included. Significant values ($p < 0.05$) are bolded.

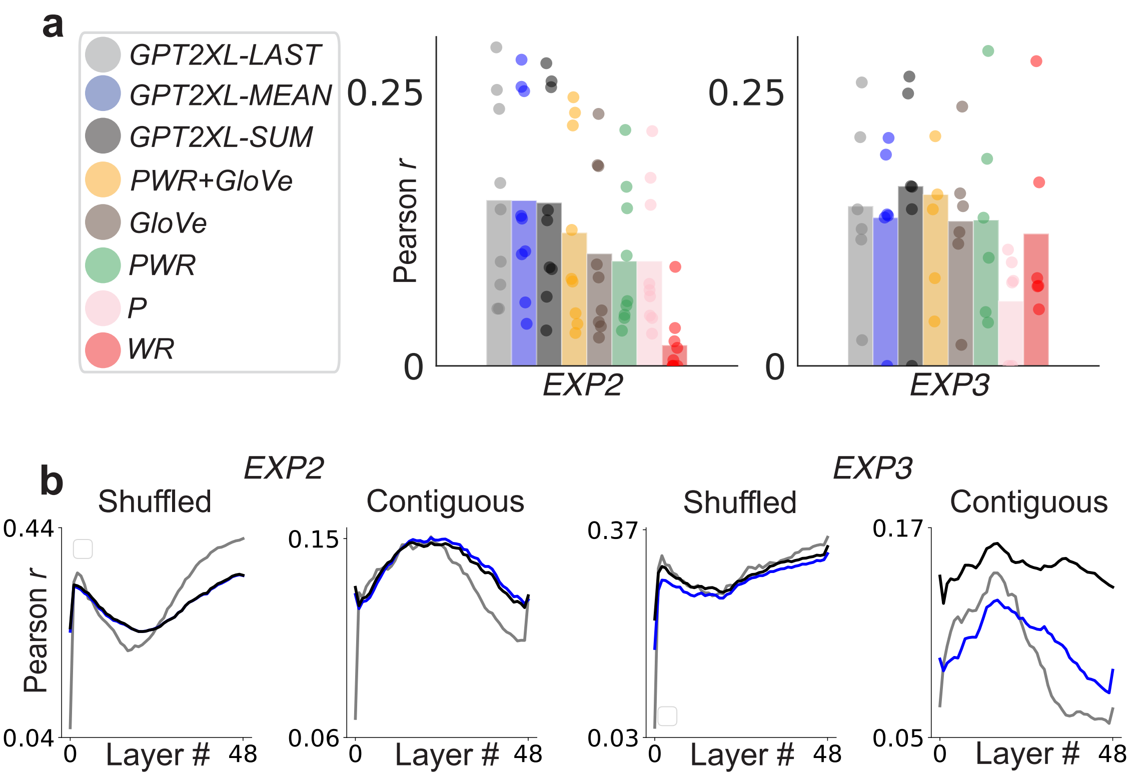

Supplementary Figure 1: **a)** Neural predictivity of models for each sub-experiment within *Pereira2018*. The *PWR* model is further divided into its sub-models: *position* (*P*) and *word rate* (WR). **b)** *GPT2XL* across-layer neural predictivity for each experiment in *Pereira2018*

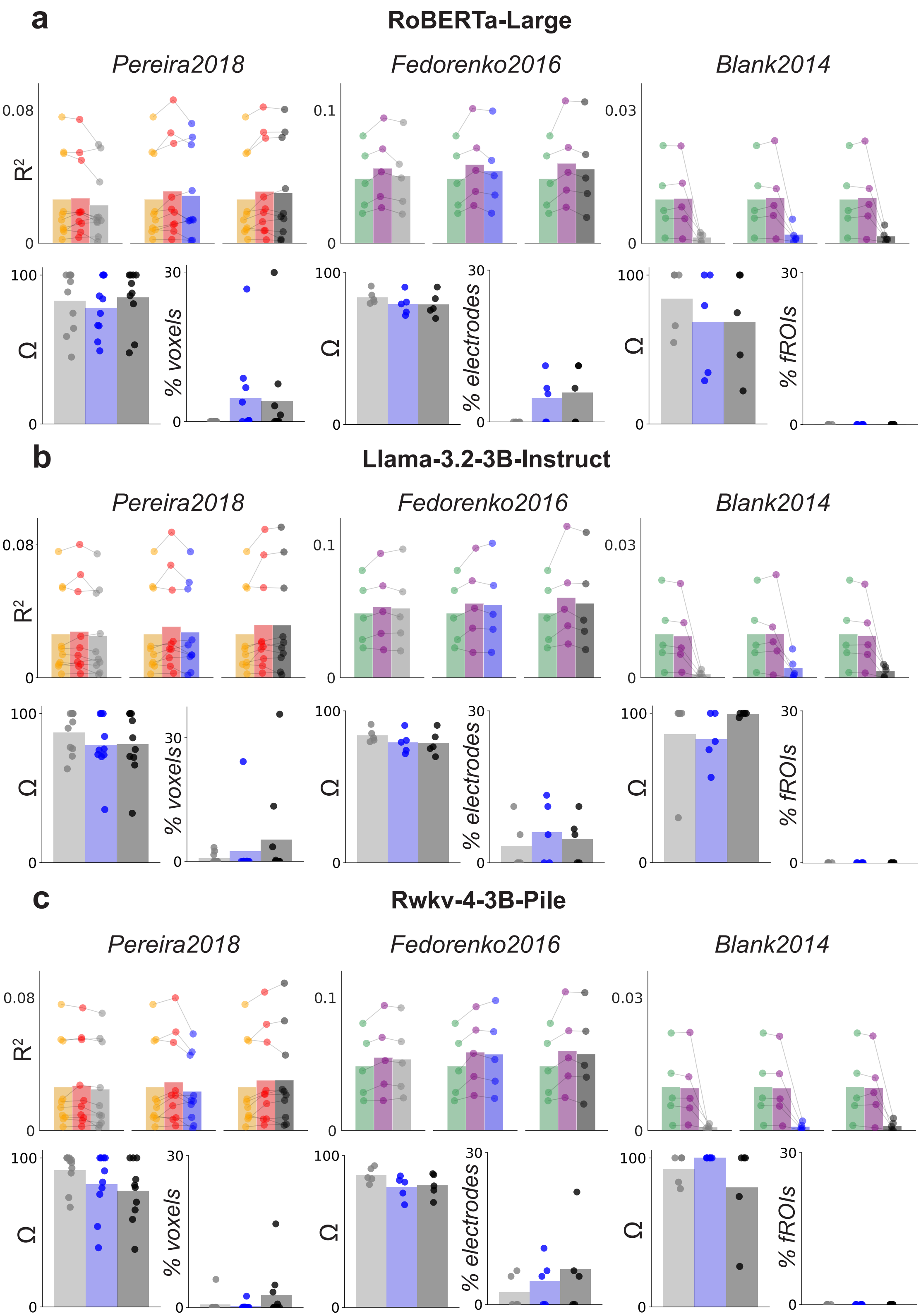

Supplementary Figure 2: Color scheme is the same as in Figure 5, except that *GPT2XL* colors now correspond to appropriate LLM. All sub-panels are identical in style to Figure 5*c* and *d*. **a)** Results for *RoBERTa-Large*, **b)** Results for *Llama-3.2-3B-Instruct*, **c)** Results for *Rwkv-4-3B-Pile*. Note that Ω values on *Blank2014* were lower with these LLMs relative to *GPT2XL* (i.e., *PWR* accounted for a lower percentage of the neural variance explained by *GPT2XL*). These values are likely lower because accounting for the neural variance that an LLM explains is unstable when LLM neural predictivity is itself very low, as is the case on *Blank2014* (see also Section 4.13). Importantly, in this dataset there were no fROIs for which any of the three LLMs explain significantly more neural variance than *PWR*.

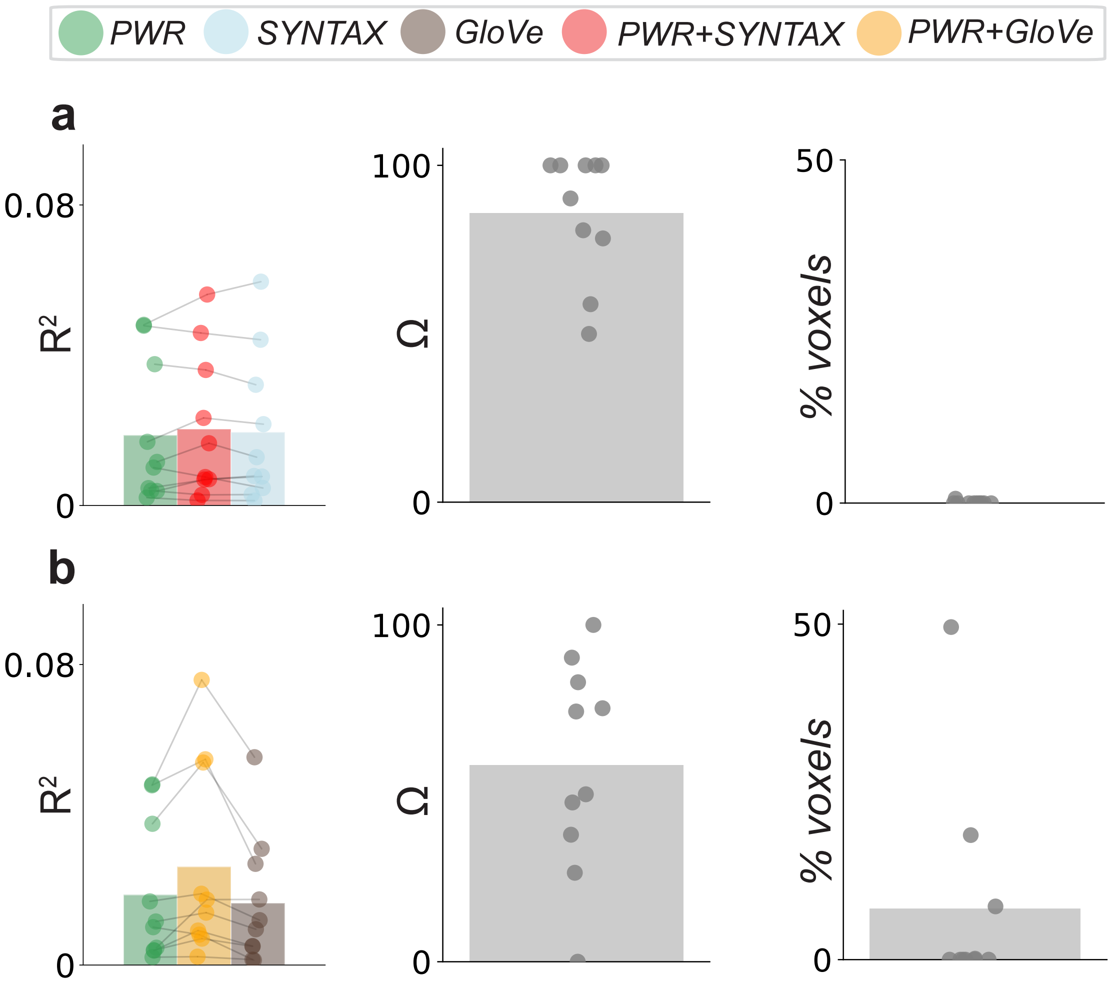

Supplementary Figure 3: **a)** Right-side shows neural predictivity for *PWR*, *PWR+SYNTAX*, and *SYNTAX*. Middle shows the percentage of *SYNTAX* neural predictivity that *PWR* accounts for, or $\Omega_{\text{SYNTAX}}(PWR)$. Left side shows the percentage of voxels where *PWR+SYNTAX* explains more neural variance than *PWR* alone. **b)** Same as **(a)**, except the *SYNTAX* model is replaced with *GloVe*. Each dot in bar plot shows values for a given participant.

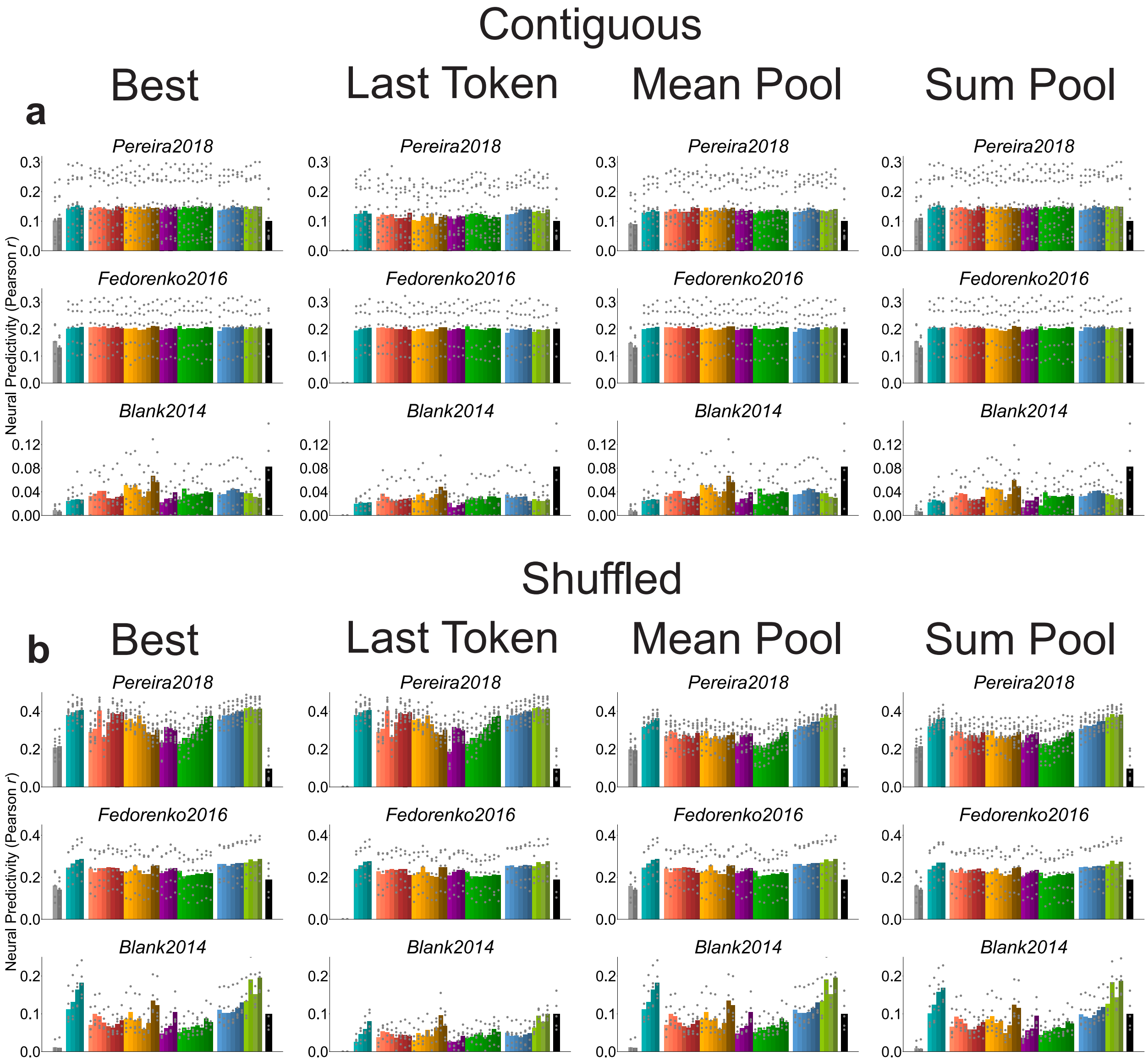

Supplementary Figure 4: **a)** Model comparisons of neural predictivity when using contiguous splits, showing the neural predictivity for each individual participant (gray dots). **b)** Same as **(a)** but for shuffled splits.

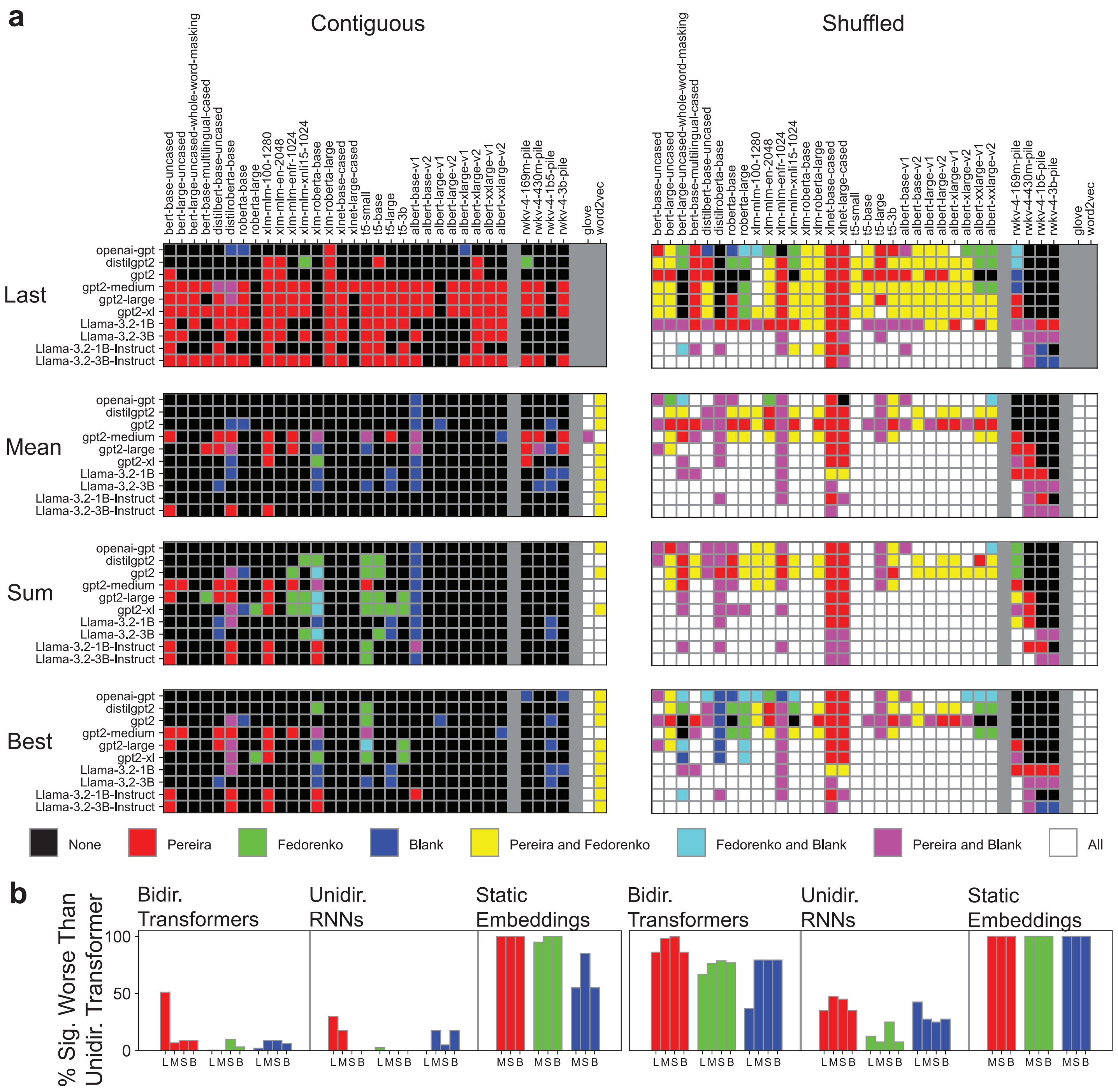

Supplementary Figure 5: **a)** Significance results (one-way Wilcoxon signed-rank test, $p < 0.05$) depicting when each unidirectional transformer model (vertical axis) significantly outperforms each model of each other model class (horizontal axis). Gray bars separate model classes (bidirectional transformers, unidirectional RNNs, and static embeddings). The color of each box depicts what combination of datasets the comparison is significant for. Note that results are omitted for comparisons with static embeddings in the last-token case, as this activation extraction method is not appropriate for non-contextual models. **b)** The percentage of significance tests for which a unidirectional transformer significantly outperforms a model for each other class of models. As in **(a)**, red, green and blue correspond to *Pereira2018*, *Fedorenko2016*, and *Blank2014*, respectively. Adjacent bars correspond to the different feature extraction methods, with L, M, S and B denoting last-token, mean-pool, sum-pool, and best, respectively.

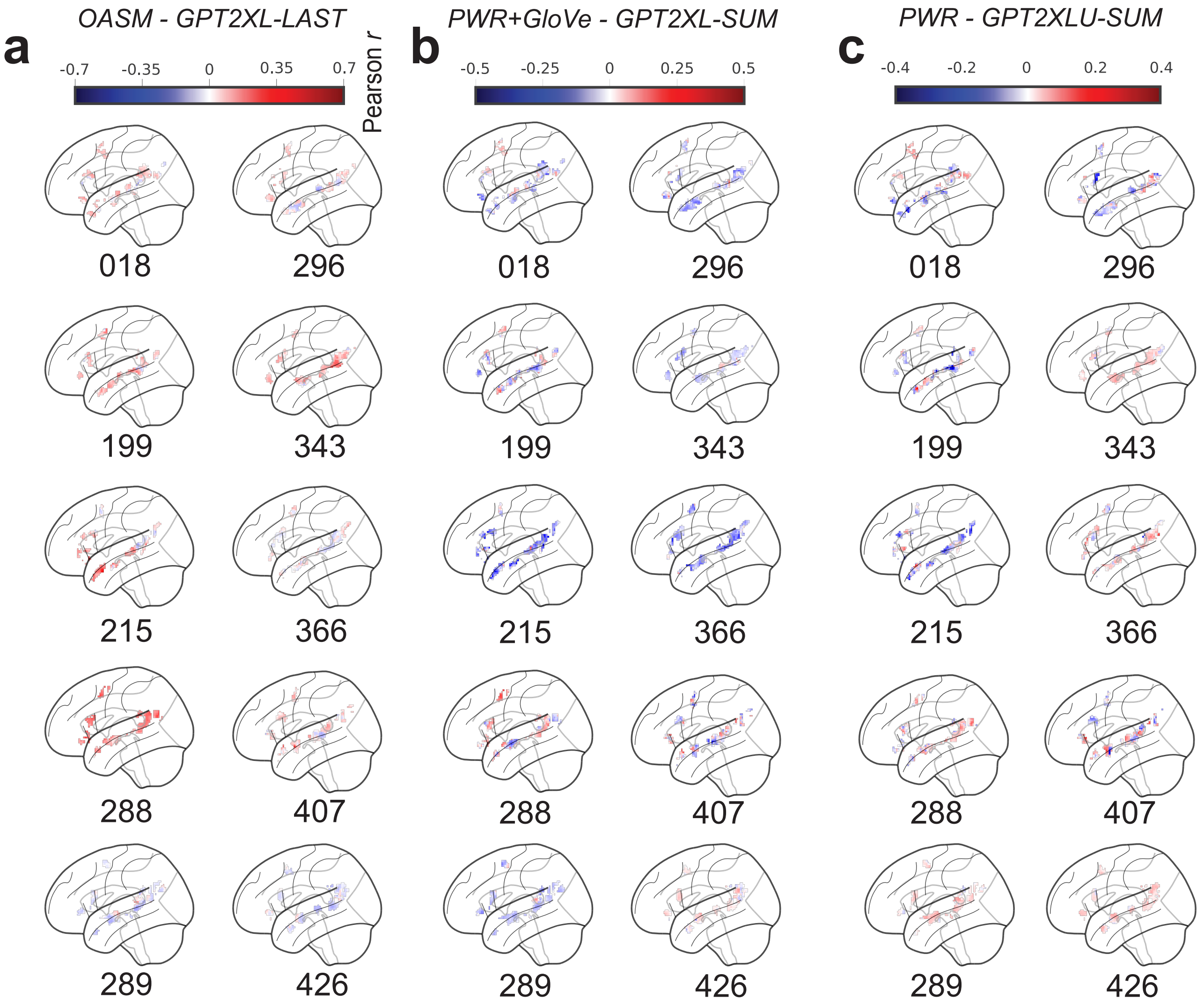

Supplementary Figure 6: Individual participant glass brain plots. Numbers below each glass brain plot indicate participant ID. Neural predictivity values are displayed in Pearson r, and only language network voxels are shown. **a)** Difference in *OASM* and *GPT2XL-LAST* neural predictivity when using shuffled splits. **b)** Difference in *PWR+GloVe* and *GPT2XL-SUM* neural predictivity when using contiguous splits. **c)** Difference in *PWR* and *GPT2XLU-SUM* neural predictivity when using contiguous splits.

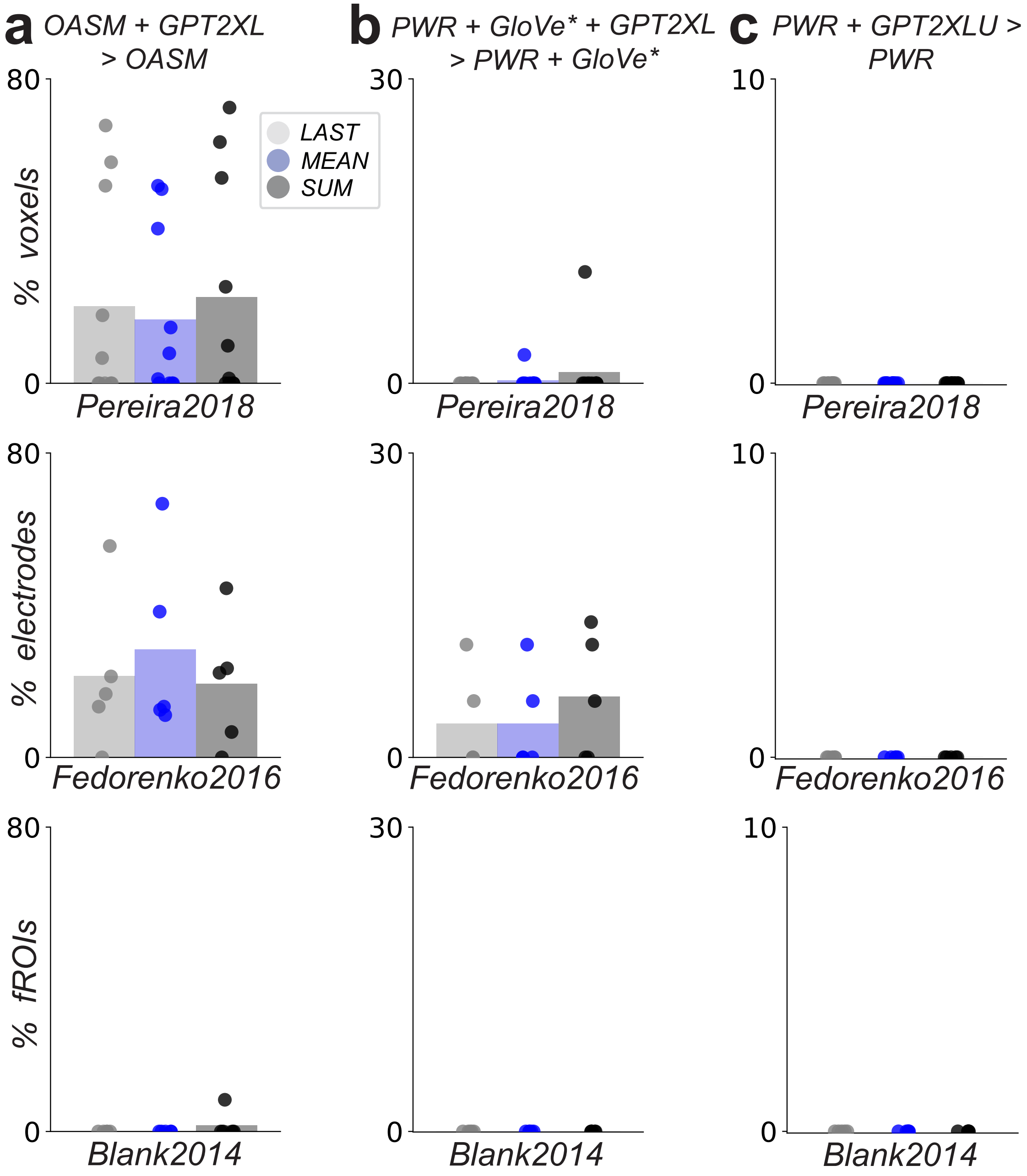

Supplementary Figure 7: Reproduction of main results obtained from using the paired t-test, except t-test was applied after averaging squared error values within passages for *Pereira2018*, within sentences for *Fedorenko2016*, and for every 10 TRs within each story for *Blank2014*. Color legend indicates activation extraction method used for *GPT2XL*/*GPT2XLU*. **a)** Same as Figure 2**d** with modified statistical procedure. **b)** Same as Figure 5**e** with modified statistical procedure. * symbol above *GloVe* indicates that *GloVe* was only used for *Pereira2018*. **c)** Same as Figure 6**e** with modified statistical procedure.

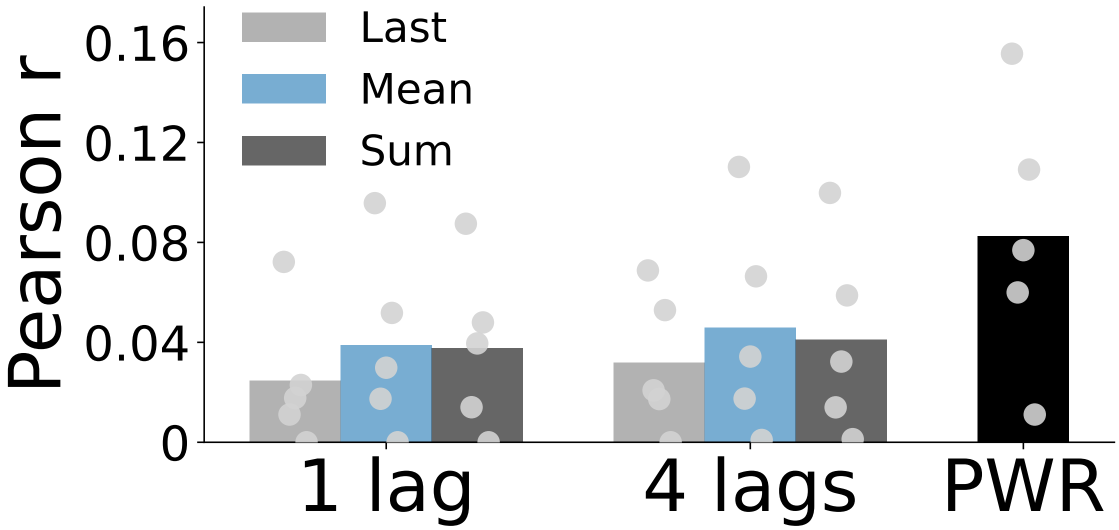

Supplementary Figure 8: Results with *GPT2XL* on the *Blank2014* dataset when using 4 lags to account for the hemodynamic response. The leftmost group of bars labeled "1 lag" shows the encoding performance when using only the tokens from the 4-6 seconds before the present TR for encoding model estimation, as in Schrimpf et al. [2021] and our main results. The "4 lags" group of bars shows the encoding performance when instead concatenating tokens from 4 lags (2-4 s, 4-6 s, 6-8 s, and 8-10 s), as is commonly done in other language encoding studies with naturalistic datasets (e.g. Huth et al. [2016]). Lastly, we show the PWR model. Although using 4 lags allows *GPT2XL* to achieve slightly higher encoding performance than when using just 1 lag, it still falls far short of *PWR*. This indicates that the relatively low performance of LLMs relative to *PWR* on *Blank2014* persists even when using a more robust and standard method to account for hemodynamic delay. Individual dots correspond to the mean Pearson r for each participant. Each feature extraction method specifies how the tokens within each 2 second TR window are aggregated.

References

Eghbal Hosseini, Colton Casto, Noga Zaslavsky, Colin Conwell, Mark Richardson, and Evelina Fedorenko. Universality of representation in biological and artificial neural networks. *bioRxiv*, page 2024.12.26.629294, December 2024.

Alexander G Huth, Wendy A de Heer, Thomas L Griffiths, Frédéric E Theunissen, and Jack L Gallant. Natural speech reveals the semantic maps that tile human cerebral cortex. *Nature*, 532(7600):453–458, April 2016.

Ariel Goldstein, Eric Ham, Mariano Schain, Samuel Nastase, Zaid Zada, Avigail Dabush, Bobbi Aubrey, Harshvardhan Gazula, Amir Feder, Werner K Doyle, Sasha Devore, Patricia Dugan, Daniel Friedman, Roi Reichart, Michael Brenner, Avinatan Hassidim, Orrin Devinsky, Adeen Flinker, Omer Levy, and Uri Hasson. The temporal structure of language processing in the human brain corresponds to the layered hierarchy of deep language models, 2024. URL https://openreview.net/forum?id=95ObXevgHx.

Andrea Gregor de Varda, Saima Malik-Moraleda, Greta Tuckute, and Evelina Fedorenko. Multilingual computational models reveal shared brain responses to 21 languages. *bioRxiv*, 2025. doi: 10.1101/2025.02.01.636044. URL https://www.biorxiv.org/content/10.1101/2025.02.01.636044v1.

Zaid Zada, Ariel Goldstein, Sebastian Michelmann, Erez Simony, Amy Price, Liat Hasenfratz, Emily Barham, Asieh Zadbood, Werner Doyle, Daniel Friedman, Patricia Dugan, Lucia Melloni, Sasha Devore, Adeen Flinker, Orrin Devinsky, Samuel A Nastase, and Uri Hasson. A shared model-based linguistic space for transmitting our thoughts from brain to brain in natural conversations. *Neuron*, 112(18):3211–3222.e5, September 2024.

Evelina Fedorenko, Michael K Behr, and Nancy Kanwisher. Functional specificity for high-level linguistic processing in the human brain. *Proc. Natl. Acad. Sci. U. S. A.*, 108(39):16428–16433, September 2011.

Martin Schrimpf, Idan Asher Blank, Greta Tuckute, Carina Kauf, Eghbal A Hosseini, Nancy Kanwisher, Joshua B Tenenbaum, and Evelina Fedorenko. The neural architecture of language: Integrative modeling converges on predictive processing. *Proc. Natl. Acad. Sci. U. S. A.*, 118(45), November 2021.

Tom Dupré la Tour, Michael Eickenberg, Anwar O Nunez-Elizalde, and Jack L Gallant. Feature-space selection with banded ridge regression. *Neuroimage*, 264:119728, December 2022.
