# Supplementary material for: Spurious alignment between large language models and brains can emerge from non-robust methods and overlooked confounds
Source: Nat Commun. 2026 Apr 27;17:5769. doi: 10.1038/s41467-026-72253-7 (PMC13324000; doi:10.1038/s41467-026-72253-7)
Supplement: Supplementary file 2 — Reporting Summary [file 41467_2026_72253_MOESM2_ESM.pdf]

nature portfolio

Corresponding author(s): Ebrahim Feghhi

Last updated by author(s): May 2, 2025

Reporting Summary

Nature Portfolio wishes to improve the reproducibility of the work that we publish. This form provides structure for consistency and transparency in reporting. For further information on Nature Portfolio policies, see our Editorial Policies and the Editorial Policy Checklist.

Statistics

For all statistical analyses, confirm that the following items are present in the figure legend, table legend, main text, or Methods section.

n/a	Confirmed	
☐	☒	The exact sample size (*n*) for each experimental group/condition, given as a discrete number and unit of measurement
☐	☒	A statement on whether measurements were taken from distinct samples or whether the same sample was measured repeatedly
☐	☒	The statistical test(s) used AND whether they are one- or two-sided<br>*Only common tests should be described solely by name; describe more complex techniques in the Methods section.*
☐	☒	A description of all covariates tested
☐	☒	A description of any assumptions or corrections, such as tests of normality and adjustment for multiple comparisons
☐	☒	A full description of the statistical parameters including central tendency (e.g. means) or other basic estimates (e.g. regression coefficient) AND variation (e.g. standard deviation) or associated estimates of uncertainty (e.g. confidence intervals)
☐	☒	For null hypothesis testing, the test statistic (e.g. *F*, *t*, *r*) with confidence intervals, effect sizes, degrees of freedom and *P* value noted<br>*Give P values as exact values whenever suitable.*
☒	☐	For Bayesian analysis, information on the choice of priors and Markov chain Monte Carlo settings
☒	☐	For hierarchical and complex designs, identification of the appropriate level for tests and full reporting of outcomes
☐	☒	Estimates of effect sizes (e.g. Cohen's *d*, Pearson's *r*), indicating how they were calculated

Our web collection on statistics for biologists contains articles on many of the points above.

Software and code

Policy information about availability of computer code

Data collection	We did not collect any neuroimaging data, and exclusively used openly available datasets. These datasets are available online in our github repository.
Data analysis	All our code was written in Python and is provided on github.

For manuscripts utilizing custom algorithms or software that are central to the research but not yet described in published literature, software must be made available to editors and reviewers. We strongly encourage code deposition in a community repository (e.g. GitHub). See the Nature Portfolio guidelines for submitting code & software for further information.

Data

Policy information about availability of data

All manuscripts must include a data availability statement. This statement should provide the following information, where applicable:

- Accession codes, unique identifiers, or web links for publicly available datasets
- A description of any restrictions on data availability
- For clinical datasets or third party data, please ensure that the statement adheres to our policy

All the datasets we used were open source, and the versions we used in the study will be provided on figshare.

Research involving human participants, their data, or biological material

Policy information about studies with human participants or human data. See also policy information about sex, gender (identity/presentation), and sexual orientation and race, ethnicity and racism.

Reporting on sex and gender	We did not perform any analyses related to sex and gender differences. The reason for this is because our study is largely in response to a previous study (Schrimpf et al, 2021) and follow up studies to it, and these studies were not focused on sex and gender differences. All the datasets we used were previously collected and are open source, and all datasets contain both female and male participants.
Reporting on race, ethnicity, or other socially relevant groupings	The study design did not consider race, ethnicity, or other socially relevant groupings.
Population characteristics	The mean age in Pereira2018 is 27.7, in Blank2014 participants were aged 18-30, and in Fedorenko2016 participants were aged from 14-29.
Recruitment	We did not recruit participants as we used existing open source datasets.
Ethics oversight	All datasets were collected under IRB approval at their respective institutions.

Note that full information on the approval of the study protocol must also be provided in the manuscript.

Field-specific reporting

Please select the one below that is the best fit for your research. If you are not sure, read the appropriate sections before making your selection.

☒ Life sciences ☐ Behavioural & social sciences ☐ Ecological, evolutionary & environmental sciences

For a reference copy of the document with all sections, see nature.com/documents/nr-reporting-summary-flat.pdf

Life sciences study design

All studies must disclose on these points even when the disclosure is negative.

Sample size	Pereira2018 contained 10 participants, Blank2014 contained 5 participants, and Fedorenko2016 contained 5 participants.
Data exclusions	We used the exact same datasets that Schrimpf et al. 2021 used, and did not exclude any data.
Replication	All our findings are reproducible, and we have taken measures to release our code and datasets and tested that our results can be reproduced.
Randomization	There were no experimental groups in our study, all participants in each dataset performed the same task.
Blinding	Blinding was not relevant to our study as we used existing open source datasets.

Reporting for specific materials, systems and methods

We require information from authors about some types of materials, experimental systems and methods used in many studies. Here, indicate whether each material, system or method listed is relevant to your study. If you are not sure if a list item applies to your research, read the appropriate section before selecting a response.

Materials & experimental systems

n/a	Involved in the study	
☒	☐	Antibodies
☒	☐	Eukaryotic cell lines
☒	☐	Palaeontology and archaeology
☒	☐	Animals and other organisms
☒	☐	Clinical data
☒	☐	Dual use research of concern
☒	☐	Plants

Methods

n/a	Involved in the study	
☒	☐	ChIP-seq
☒	☐	Flow cytometry
☐	☒	MRI-based neuroimaging

Plants

Seed stocks	N/A
Novel plant genotypes	N/A
Authentication	N/A

Magnetic resonance imaging

Experimental design

Design type	The task design consisted of participants passively comprehending linguistic materials, presented either in an audio or visual format
Design specifications	In Pereia2018, Experiment 2 consisted of 96 passages and Experiment 3 consisted of 72 passages. A single fMRI scan was taken 4 s after visual presentation of each sentence. In Blank2018 fMRI responses were recorded continuously for each of the 8 stories.
Behavioral performance measures	We did not use any behavioral information collected as part of these datasets.

Acquisition

Imaging type(s)	Functional
Field strength	Both Blank2014 and Pereira2018 was recorded with a 3-Tesla scanner
Sequence & imaging parameters	From the original Blank2014 paper: "functional BOLD data were acquired using an echo planar imaging sequence with a flip angle of 90° and applying generalized autocalibrating partially parallel acquisition with an acceleration factor of two."<br>From the original Pereira2018 Paper: "Functional, blood oxygenation level-dependent data were acquired using an EPI sequence (with a 90° flip angle and using GRAPPA with an acceleration factor of 2), with the following acquisition parameters: 31 4 mm thick near-axial slices, acquired in an interleaved order with a 10% distance factor; 2.1 mm×2.1 mm in-plane resolution; field of view of 200 mm in the phase encoding anterior to posterior (A>P) direction; matrix size of 96×96 voxels; TR of 2000 ms; and TE of 30 ms."
Area of acquisition	Whole brain

Diffusion MRI ☐ Used ☒ Not used

Preprocessing

Preprocessing software	Data was provided in a preprocessed format. We did not provide any additional preprocessing.
Normalization	We did not perform any normalization beyond what was done by the original authors.
Normalization template	All analyses were performed for each subject individually, with the exception of glass brain plots. For the glass brain plots, we
Noise and artifact removal	We did not perform any additional noise or artifact removal.
Volume censoring	Did not perform any additional volume censoring.

Statistical modeling & inference

Model type and settings	We performed voxel-wise vregressions to predict activity of each voxel given model activations. We did not use any random effects.
Effect(s) tested	We compared how well different models predicted voxel activity used R2 and pearson r metrics.

Specify type of analysis: ☐ Whole brain ☐ ROI-based ☒ Both

Anatomical location(s)	We analysed language-responsive voxels and electrodes, and these voxels and electrodes were selected using a functional localizer task.

Statistic type for inference<br>(See Eklund et al. 2016)	When determining whether one model was more significant than another, we perform analyses voxel-wise with results FDR corrected within each participant.
Correction	We used FDR correction within each participant.

Models & analysis

n/a	Involved in the study
☒	☐ Functional and/or effective connectivity
☒	☐ Graph analysis
☐	☒ Multivariate modeling or predictive analysis

Multivariate modeling and predictive analysis	We fit linear encoding regression models to predict voxel responses given linguistic representations of computational models.
