# Supplementary material for: Spurious alignment between large language models and brains can emerge from non-robust methods and overlooked confounds
Source: Nat Commun. 2026 Apr 27;17:5769. doi: 10.1038/s41467-026-72253-7 (PMC13324000; doi:10.1038/s41467-026-72253-7)
Supplement: Supplementary file 3 — Transparent Peer Review file [file 41467_2026_72253_MOESM3_ESM.pdf]

nature portfolio

Peer Review File

Spurious alignment between large language models and brains can emerge from non-robust methods and overlooked confounds

Corresponding Author: Mr Ebrahim Feghhi

This file contains all reviewer reports in order by version, followed by all author rebuttals in order by version.

Version 0:

Reviewer comments:

Reviewer #1

(Remarks to the Author)
This paper presents a careful re-evaluation of claims regarding the alignment between large language models (LLMs) and neural responses. The authors demonstrate that certain analytic choices, particularly the use of shuffled train-test splits and specific activation extraction methods can lead to inflated or misleading estimates of neural predictivity. They introduce useful baselines (e.g., the Orthogonal Autocorrelated Sequences Model) and show that simpler confounds such as position and word rate can explain much of the observed variance.

While it is a valuable contribution, there are significant concerns about the tone and framing of the paper, particularly the title and overarching claims. The title suggests that the entire filed of model-to-brain alignment is illusory, but the authors' own results do not support this level of generality. In fact, their results show that when methodological issues are addressed, i.e., with contiguous splits, appropriate regularization, and validated baselines, LLMs still predict neural data to a meaningful degree. In that case, it is not appropriate to call it an "illusion," as overstating risks undermining a field that is still evolving. If the goal is to correct methodological flaws in a few highly cited papers, largely from a small number of labs, that message should be delivered more precisely, as much of LLM-brain alignment work uses the very practices the authors endorse.

- The authors argue that next-word prediction is not robustly correlated with neural predictivity, but this conclusion seems too strong. As they themselves note, the effect of model family is large, and within certain families (e.g., unidirectional transformers), there is a clear scaling trend in Figure 3 consistent with earlier work (e.g., Pasquiou et al., 2022). While the across-family correlation is fragile, this within-family effect is non-trivial and deserves a more careful discussion. The authors mention this in passing but do not highlight that they essentially replicate that earlier trend.

- Furthermore, the paper does not specify how perplexity was computed. Was it on a standard benchmark, or on the same stimulus texts used in the neural datasets? This omission is important, especially given the short stimuli used in some datasets (e.g., Pereira2018, Fedorenko2016). Clarifying this would strengthen the argument.

- Additionally, one should be cautious against plotting perplexity across unidirectional and bidirectional models in the same space. These architectures are trained with different objectives (causal vs. masked language modeling), and bidirectional models are inherently out-of-domain for next-word prediction tasks. Their higher perplexity is not necessarily indicative of weaker language modeling but of task mismatch. This conflation likely dominates the pattern in Figure 4, and the same issue was present in Schrimpf et al. (2021). The authors could be more explicit in acknowledging and adjusting for this discrepancy.

- The authors justifiably adopt L2-regularized regression, but it's worth noting that the performance of larger models like GPT2-XL may still suffer due to overparameterization relative to the size of the datasets. This is particularly important for fMRI datasets with limited samples. Regularization can mitigate but not eliminate this challenge, and the authors might consider discussing this limitation more explicitly, especially when comparing large and small models.

Minor Points
• Missing references on lines 343 and 539

• The writing is clear overall, though some claims could be tempered to better reflect the of the results.

(Remarks on code availability)

Reviewer #2

(Remarks to the Author)
This paper addresses an important reproducibility issue pertaining to aligning brain activity and LLM representations (though really this affects any type of representation). The authors show that some recent results, specifically those of Schrimpf et al. 2021, are not robust. One aspect is due to the use of shuffled splits when evaluating models, which inflate significance due to the autocorrelation in time, and the other is due to the effect of low level features which could explain most of the predictivity observed by LLMs. After adjusting for some of these effects, the authors find that even the claim that models with better perplexity are better predictors of brain activity does not replicate.

Honestly, it is surprising that shuffled train-test splits were used at all in the studies mentioned. This problem has already been identified in work prior to 2021, some of which cited by the authors. fMRI is notoriously temporally autocorrelated. One solution if one insists on using shuffled train-test splits is to throw buffers of data (for example 20 seconds) on each end of the splits. The problem is that this throws away a lot of data especially in limited datasets. This detail (throwing a buffer away) could be a small addition to the discussion section for completeness.

The results with OASM are very compelling, and so are the effects of different ways to extract features and the way that low level features are tested. However, I think there is an important improvement to be made:
- A check, in lines 627-629, the N for "this test" corresponds to the number of time points that were tested? You can't do a t-test on correlated time points (actually, isn't the entire point of the paper that time points are correlated?). One way to do this test is to block the time course of errors into large blocks that are slower than fMRI (e.g. 20 sec), and then use a block permutation test to show that the errors of M+LLM is different from M.
- An additional thing to add would be to illustrate the different components of the variance partitioning shared, unique etc) on the brain.

Are some of the differences in the Fedorenko 2016 results due to the fact that it's electrophysiology and thus the data has a faster mixing rate / is autocorrelated with a shorter lag? This should be discussed in the relevant result sections.

Can the authors comment on focusing on the layer that achieves the best neural predictivity? The way it is stated it sounds like it might be double dipping (line 69 and especially lines 617 5o 629).

Finally, while the paper is an important contribution, it might be misconstrued by too hasty a reader to mean that all reports an alignment between LLMs and brains are illusory. The title could be perhaps made more clear, or alternatively a line can be added to the abstract clarifying that these limitations do not affect all studies, and that some studies do control for low level properties like positional signals and word rate.

Anyways I think that this paper addresses an important caveat of many recent studies. Results of small modifications to a model without controls are over-interpreted. In any field of science, one experiment should not lead to a convincing answer. It is the robustness of results across studies that should lead to a trusted hypothesis.

Tiny typos: line 59: acquired after the presentation. Line 343 ?

(Remarks on code availability)

Reviewer #3

(Remarks to the Author)
Summary

An influential finding in the field of cognitive neuroscience is that one can process text word-by-word with AI models (or large language models --LLMs) and use the resulting LLM-based representations of text as predictors of human brain activity recorded while participants listened or read the same text. It turns out that one can predict the brain data quite well (up to some data-inherent noise ceiling) on the basis of LLM representations. A common big-picture interpretation is that the way LLMs process text is "brain-like", which can be viewed as intriguing given that contemporary LLMs are primarily collections of clever engineering tricks for practical applications rather than biologically-inspired or cognitively-inspired models.

One of the more influential studies that showed this phenomenon across a range of LLMs and brain datasets is the study by Schrimpf et al, (2022), which inspired decent number of follow-up studies. The authors in the submitted manuscript challenge the main findings in the original work on methodological grounds -- a specific choice of how the test datasets to evaluate neural predictivity were constructed was suboptimal given known properties of brain data.

Specifically, the brain signals are temporally autocorrelated, meaning that the data samples recorded nearby in time tend to

be similar. The original work allowed the data in the training set and the testing sets to come from the temporally adjacent parts of the dataset by randomly partitioning the datasets into train/test splits ("shuffled splits"). The concern arises that the reported, and influential, high neural predictivity scores were achieved because the mapping between LLMs and brains exploited this spurious feature: similarity of train and test data points due to temporal autocorrelation, rather than genuine LLM brain-likeness.

The authors of the submitted manuscript re-analyzed the datasets by Schrimpf et al (2022) with (mostly) the same LLMs but this time not allowing training and testing data points to be collected from nearby in timepoints. Test dataset came from separate passages different that the ones in the training set ("contiguous splits" approach). It turns out that neural predictivity evaluated this way is indeed much lower than originally reporte reported neural predictivity correlation by constructing a model that only predicts brain data based on temporal adjacency --- it turns out the model fits as well or better than LLMs. This methodological flaw then propagates through other findings in the study.

All in all, this study an impressive body of work and a welcome addition to the literature and a contribution to the community. The rationale is sound, the results are convincing (and robust over the choice of metrics and different models), and I find the interpretation is justified and constructive given the reported data. I have no problems seeing this published. I do have some clarification requests which I would love to see addressed before I can recommend publication. I think authors should not have trouble addressing this in revision.

Major

1. State deviations in methods and results from Schrimpf et al. (2022) explicitly. On l. 90 you state "using largely the same methodological choices implemented in Schrimpf et al." It would be useful to briefly state whether any important deviations from Schrimpf et al. were made (main text or in online methods). Similarly, please consider indicating, at least when reporting the first batch of results in Figure 1 whether your LLM predictivity scores on shuffled splits are in the same ballpark as reported by Schrimpf et al (i.e. whether you replicated their success). Your main point stands regardless, but this would be welcome for completeness.

2. "The activations of OASM are orthogonal for distinct passages". I think spending a line our two adding more implementation details would be welcome (and refer to the online methods for the rest). Or consider adding a visualization that shows (here or in the online methods) example "activations" in OASM across say 3-4 passages? The concise description of OASM design is already pretty good, but I think it that would help readers getting additional intuition for OASM and the problem it addresses.

3. l. 93 "activations from GPT2XL using the last token of the stimuli" --> I might be blanking on the details from Schrimpf et al (2020), but how does this "last-token" method work for Fedorenko2016 (ECoG) dataset? In 4.1. you state that the ECoG dataset consisted of a 416 x 97 (words by electrodes) matrix. How do you model that dataset with a single (last/sum/mean) embedding per sentence? Or were the neural data averaged per sentence? Also, it could be useful to briefly remind readers with a short sentence in the main text (as you do in supplementary materials) why it is necessary to keep one embedding or average (due to fMRI temporal resolution etc.). Otherwise it isn't immediately clear from the text.

4. l. 127 "we use a one-sided paired t-test across square error values". What are the square error values referred to here? Sigmas? R^2?

5. Fig 2b: If each dot in these scatterplots is a unit of analysis (voxel/electrode/fROI) for all participants -- how does one read the color-coded Pereira2018 figure? The color indicates #voxels but each dot is a voxel? Please clarify.

6. l. 245. "LLM-based model of contextual syntactic representations" --> I really appreciate succinct reporting in the main text over all. I would just consider adding here a more descriptive sentence for "contextual syntactic representations". For example, "contextual syntactic representations obtained by averaging transformer embeddings extracted from sentences with the same part-of-speech and dependency tags, but different content words" or similar. Syntax means many things to different scholars and it is useful to briefly highlight your choice in the main text. If at all possible, I would also recommend showing (in supplementary materials) as small table with a sample of these generated sentences (and possibly their loss scores). Such that readers can get an impression of what is being averaged over.

7. Modality effect. It seems to me (Figs 1.b and 2.d) that OASM accounts for more neural predictivity in the listening dataset (Blank2014) compared to the two reading datasets. I am curious if this in your opinion warrants a small comment in discussion: Is it the case that the temporal autocorrelation is a a more pronounced problem in auditory stimulation than reading? Or is it just that LLMs happen to explain less variance in the auditory dataset to begin with?

8. l. 249. "we find that a combination of positional signals, word rate, and static word embeddings predicts almost as much — or more — neural variance than GPT2XL". --> I wonder if you can speculate -- again, briefly -- on what your results say on transformer architecture. Some (most?) transformer-type models, like GPT2, have both "static embedding" and "positional embeddings" (layer 0 in your reporting) that get mixed into the hidden layer representations ("intermediate layers" in your terminology). But layer 0 don't seem to be competitive in predicting neural data based on eye-balling Fig 2.e. But based on the conclusions in this report (which I'm sympathetic to) and the logic behind OASM, wouldn't we expect the initial transformer layers to be the most competitive?

9. Compute resources. Would it be possible to add a section in Supplementary materials (or similar) on approximate computational resources that you used to run an experiment. I think even just a ballpark indication for the least and the most intense jobs would be helpful for anyone attempting to reuse the code (e.g. To run experiment on dataset 1 with 1 model, we requested between XY amounts of RAM on a ZY cluster and GPU?)

Minor

- l.86 "we constructed a trivial model" --> In the context of regression, the term model is reserved for regression coefficients -- OASM is a predictor/design matrix, right? (I understand the term model is loaded and and used across the board, but would be helpful for clarity).

- l. 67 "each outer fold": Terminology (inner-outer folds) not introduced at this point in Introduction. Perhaps add a generic sentence layout out the logic of cross-validation (e.g. "In cross-validation, the dataset is split into N folds, ... on each fold...") to make it clearer.

- l.177 "in the Blank2014 dataset" --> Add figure reference here (Fig 3.a)

- Stijn Hawinkel et al. reference lacking year.

- l. 270. "used in the most impactful study on LLMs" --> unless you provide a quantification, I would say "in one of the most impactful studies"

- Fig 2c: Is there a need to plot yellow and purple bars 3 times within a panel? I assume you've considered this, but wouldn't it be fine to just plot five bars (one for each model combination) since yellow and purple are the same currently?

- l. 343. "On the other hand," --> Missing citation reference in bibliography (see "?" symbol)

- l. 480 "scipy gaussian_filter1d function" --> Do make sure to cite the main software packages you used that provided critical functionality (e.g. Scipy, Numpy, transformers, pytorch). Most provide citation information on their websites (e.g. https://numpy.org/citing-numpy/).

- l. 539 "L2 penalty" --> Missing citation reference in bibliography (see "?" symbol)

(Remarks on code availability)
I thank the authors for submitting the code and code checklist! I did not thoroughly review the code base nor have I attempted to reproduce any experiments. I can confirm that a cursory documentation is available on the repository on how to use the code. The dependencies are provided as well and the code for reproducing the figures as well. Given my quick impression, it's a well-structured research code in most parts and I was able to quickly get an impression what a specific piece of code was trying to achieve.

I suspect (based on quick impression) that actually reproducing and reusing code might not be super straightforward. For example, the user is instructed to use `call_banded_reg.py`, but it comes with 10+ input arguments. These arguments have defaults and help entries, which is great, but it would be nicer to just provide an small instructive example of how this function can be called (For example, "To run a GPT2 model on Fedorenko dataset, you can call it like this"). The organization is lacking in some parts. For example, I found lingering (?) `breakpoint()` statements in the code, the root of the repository contains `figure4_updated.py` which seems it belongs elsewhere, notebook files are stored along with python modules etc. I believe someone with extensive experience of python would be able find their way, however.

Being aware of the realities of code development and sharing in research, it seems in an acceptable shape for a research project code and will provide value to the researchers interested in this work.

Do make sure to include the license file in the repository as well.

Reviewer #4

(Remarks to the Author)
Hadidi, Feghhi and colleagues show that certain results from a particular thread of papers fall victim to a methodological misstep: using cross-validation with shuffled train and test splits. For many types of data (e.g., fMRI data, natural language, or basically any time series data), using shuffled train and test splits means that samples in the test sets will be correlated with samples in the training sets due to autocorrelation. They show that two core results from a landmark paper by Schrimpf et al., 2021, using shuffled splits, can be captured by a simple “orthogonal autocorrelated sequences” model (OASM). These two results, (1) that unidirectional / autoregressive models (like GPT) outperform bidirectional / masked models (like BERT) and (2) that brain predictions correlate with next-word prediction, both effectively disappear in the Schrimpf et al., 2021, datasets when using properly contiguous cross-validation splits instead of shuffled splits. They also show that, under certain conditions, a simple “position and word rate” (PWR) model provides surprisingly high correlations on par with the language models.

This is a very timely paper, and likely to be somewhat controversial—although I think this paper is unequivocally correct in

pointing out certain methodological missteps that undermine core results in prior work. I have two high level concerns: (1) that some readers will overgeneralize these findings and “throw the baby out with the bathwater”, and (2) that some of these “gotchas” are also due in part to relatively odd structure of the original datasets (as well as certain analytic choices that remain unexplored here). This creates a certain tension between accurately reproducing (suboptimal) analytic choices applied to particular datasets used by Schrimpf et al., 2021, and making more general recommendations for the field. I unpack these comments below, followed by a number of more specific methodological questions and clarifications. Overall, I think this is a useful paper, and correctives of this kind are part of a healthy science—but, for a paper like this, the authors need to be extremely judicious and precise in their application of the corrective to avoid causing more harm than good.

Major comments:

1a. This is my highest-priority comment: I think it’s extremely important that the authors be very precise about which papers—and which associated results—are known to be affected by certain methodological missteps, and explicitly which studies (and results) are not. There are already plenty of people out there who would love ammunition to argue that (1) encoding models are generally useless for neuroscience (not true at all) and (2) that neural network models for natural language (e.g., LLMs) are not useful for modeling the human neural machinery for natural language (also not true). There is a real risk that the important correctives this paper highlights will be overinterpreted as an indictment of the entire research program—which includes many labs and papers that have in fact been very careful and have managed to avoid these methodological missteps. (This kind of pattern—where a legitimate corrective is introduced, but then overinterpreted—occurs repeatedly in the fMRI literature; for example “voodoo correlations”, “cluster failure”, etc; Vul et al., 2009; Eklund et al., 2016.) I think the authors are already making a good-faith effort to do this, but the more judicious and explicit the better in this respect.

1b. Concretely, I would first suggest that the authors more explicitly and comprehensively enumerate which papers are subject to particular criticisms. For example, as a spot-check for shuffled cross-validation, I confirmed that Caucheteux et al., 2021, 2022, 2023, Caucheteux & King, 2022, all explicitly state in writing that they’re using temporally contiguous splits for cross-validation. Similarly, work from Huth and colleagues typically uses a completely held-out test stimulus; e.g., Antonello et al., 2021, 2023; Antonello & Huth, 2024. Toneva et al., 2022, cross-validate to separate scanner runs. All work from Hasson and colleagues (that I have been personally involved with) has used contiguous cross-validation folds; Goldstein et al., 2022, 2025; Hong, Wang et al., 2024; Kumar, Sumers et al., 2024; Zada et al., 2024; and some studies, like Goldstein et al., 2024, put more stringent tests of generalization at the core of the work. This is of course a biased selection based on my own personal familiarity, but a spot-check of this kind suggests to me that a majority of published work does *not* directly fall victim to the methodological issue of shuffled cross-validation splits. I know it’s quite difficult to verify and list out all non-offenders in this way, but I think it’s incumbent on the authors to ensure that specific criticisms, e.g., about missteps in cross-validation, do not bleed over into studies that put in the effort to do things right.

1c. I would also suggest that authors be very clear that failing to replicate certain results from Schrimpf et al., 2021, does not necessarily mean those results won’t hold up elsewhere. For example, the correlation between encoding performance and perplexity in Figure 4 has been found by Caucheteux & King, 2022, in both fMRI and MEG, using much larger samples (fMRI N = 100, MEG N = 95). Antonello & Huth, 2024, also replicate this result in a small-N dataset where each subject listened to *5 hours* of naturalistic story stimuli (despite using this result to illustrate a different point). On the other hand, the samples sizes here are quite small (N = 10, N = 5, N = 5) and the stimuli are generally non-naturalistic and more simplistic / impoverished (at least in Pereira2018 and Fedorenko2016; which may undercut the power of LLMs). This suggests that certain results under consideration here are not invalid generally, just that they cannot be replicated using the *specific* datasets from Schrimpf et al (and here) and the *specific* methodology used by Schrimpf et al. There are many possible reasons for this that do entirely hinge on the methodological correctives discussed here; for example, these results may not replicate because the structure induced by the experimental design (e.g., stimulus on/off blocks, matched/isolated sentences, etc) may overpower the structure of the actual language stimuli; or the language stimuli may not be sufficiently diverse or engaging or meaningful to subjects.

1d. By “specific methodology”, the first thing that comes to mind is that Schrimpf et al., 2021, use a very restrictive set of voxels based on individual-specific functional localizer tasks. This analytic choice is imported directly into the current work and otherwise unexplored. These tasks simply contrast actual sentences versus nonword sentences, and will tend to focus on regions that have uniformly high activation for the actual sentences (greater than the nonword sentences). On the other hand, encoding performance in an analysis of this kind will be driven by reliable *variance* across words/sentences with different meanings (the model-based predictions are mapped onto up-and-down fluctuations in the signal across TRs / words / sentences; if the signal is uniformly high, it would result in poor encoding performance). Other research groups have compiled a large body of results indicating that different components of natural language comprehension extend beyond these areas (e.g., Huth et al., 2016; Nastase et al., 2021), and many voxels outside these regions exhibit strong encoding performance with LLMs (e.g., Caucheteux et al., 2022; Kumar, Sumers et al., 2024). I don’t know if this is true, but it’s certainly possible that this particular way of defining language regions yields a set of voxels that are unusually well modeled by OASM- or PWR-style models. My point here is that, as written, the current set of non-replication results are limited to this method for selecting voxels of interest. If the authors want to make broader claims, they would have to reproduce these null results across a wider set of voxels.

2. My second high-level comment (related to comment 1c): One of the sources of my confusion when reading this is that I hadn’t realized quite how (let’s say) “weird” the datasets used by Schrimpf and colleagues (2021) were. As someone who primarily uses longer-form, naturalistic stories/narratives as stimuli, I found myself wondering how many of these methodological and interpretational gotchas are partly symptoms of using these oddball datasets. For example, the authors

say these results “depend somewhat unpredictably on the choice of dataset, models, and methods.” This is patently true of many results, but all datasets are not created equal—examples where some of these results hold up are in much larger, more diverse datasets (see comment 1c). If the authors’ goal is to show that this result does not hold up in the oddball Schrimpf et al., 2021, datasets alone, then that’s fine, and they should be very explicit about that. If the authors want to claim that this result does not hold up *in general*, they would need to reproduce the null result on much larger, more diverse, more naturalistic datasets used by other groups; for example, LeBel et al., 2023, Nastase et al., 2021, Zada et al., 2025, or Li et al., 2021. At the very least, the authors should discuss the limitations of the datasets used by Schrimpf et al., 2021, versus larger, more naturalistic datasets.

3. Related to the previous comment, I think the authors should unpack the structure of the datasets in a bit more detail, as this will impact the interpretation of their findings. For example, I stumbled when I got to line 58: “participants read short passages presented one sentence at a time, and a single fMRI volume (TR) was acquired after presentation of each sentence.” That’s strange. I understand that during a reading task, timing of fixations will be variable / idiosyncratic. Does this mean participants read the sentence, pressed the button to indicate they were finished, and following data were used for analysis? The scanner is presumably acquiring TRs continuously across an entire run, so I assume “a single fMRI volume (TR) was acquired” actually means that all *other* TRs acquired during sentence reading and the intervening gap were *discarded* for analysis. What is going into the context window when extracting embeddings for a dataset like this? In a paper like this, I don’t think it’s sufficient to simply redirect the reader to the original papers for further detail.

4. Following on the previous comment, I’m curious exactly how these datasets were preprocessed and whether the particularities of the preprocessing may impact the results observed here. For example, if linear drifts were not factored out of the fMRI time series, this would certainly contribute to the success of the OASM model. If the on/off structure of stimulus blocks was not accounted for in some way, this could both (a) hurt LLM encoding performance (due to large stimulus on/off signal fluctuations that are not related to the linguistic content), or (b) enhance the performance of the PWR model (e.g., if the signal ramps up at the beginning of a stimulus block, then tapers off over the course of the sentence). For example, in Zada et al., 2025, we explicitly include low-level confounds as well as task-level on/off block regressors to rule out these factors.

5. It should be explicitly noted that, at least since Huth et al., 2016, many fMRI studies in this vein have included low-level confound regressors like word rate; e.g., Huth et al., 2016, used word rate, phoneme rate, and phonemes alongside their semantic model of interest; similarly for our work in Kumar, Sumers et al., 2024, where we include phonemes, phoneme rate, word rate, and a silence indicator in a separate nuisance band using banded ridge regression (linguistic features of interest will be correlated with these low-level confounds in most cases of natural language).

6. At lines 555 and 608, you say “For R-squared, we clip results to be at least 0 at the voxel / electrode / fROI level to prevent noisy values from biasing the mean downwards.” Is there any precedent for doing this fairly aggressive data imputation? In my experience, out-of-sample R-squared values in this kind of analysis can be *very* low, with many going strongly negative (i.e., providing poorer predictions than the mean on the test set). This can be a useful tool for diagnosing whether the modeling pipeline (cross-validation, normalization, hyperparameter selection, etc) are working properly. I’m curious about what proportion of voxels end up getting clipped in these analyses… For example, if you only have 243 voxels to begin with (Pereira 2018, Experiment 3) and half of them disappear due to negative R-squared values, that would give me major pause when interpreting the current results.

7. I was a bit confused about the statistical tests used for model comparison. For example, at line 126, you say: “Third, because Ω is defined at the participant level, we use a one-sided paired t-test across square error values to determine the percentage of voxels / electrodes / fROIs where OASM+GPT2XL explained significantly more neural variance than OASM alone. We applied FDR correction for each participant separately for this analysis.” What are the samples going into this t-test if you’re doing the test within each participant? (i.e., what are the replicates determining the degrees of freedom in the test?) If I’m reading the Methods correctly, starting at line 617, you’re treating voxels (e.g., N = 384) within the language ROIs as samples? This is fairly nonstandard, and I’m not really sure what inferential interpretation it has. I’m also not sure how that relates to the term “percentage” in your description of the test. Alternatively, you might be supplying sentences or test folds into the statistical test? A different approach here to performing a within-subject statistical test would be to randomize the mapping between the time series and the embeddings somehow; e.g., phase randomization, random circular rotation of the time series, or block-wise permutation (as in Lebel et al., 2023). When you say “FDR correction for each participant separately”, FDR correction across *what* multiplicity of tests exactly? Across different model comparisons? I don’t necessarily think any of this is wrong; but please clarify these points in both the Results and the Methods.

8. At line 92, you indicate that Schrimpf and colleagues (2021) used ordinary least squares (OLS) to map GPT-2 XL embeddings onto the brain activity. Is this correct? I don’t understand how this worked. Aren’t the GPT-2 XL embeddings 1,600 features wide, with many fewer samples (TRs) in the fMRI data? (i.e., 384 and 243 samples in Pereira2018, 416 samples in Fedorenko2016, 1317 samples in Blank2014…) Wouldn’t it be impossible to solve such a regression equation with OLS? Was Schrimpf doing PCA on the embeddings prior to regression? Or some pseudo-inverse trick? My understanding is the authors somehow replicated the same analysis using OLS (lines 92 and 533). How? In any case, I agree that the authors should focus on ridge for the core results, as this has become the standard in the field.

9. At line 542, you say “We z-score all predictors across samples before training regressions.” I’m not demanding the authors re-run their models, but this is not the best practice. Z-scoring the entire dataset prior to cross-validation will introduce leakage between training and test sets. For a paper like this that hinges on methodological rigor, ideally you should z-score within the training set of each cross-validation fold; e.g., use scikit-learn’s StandardScaler in a Pipeline to .fit_transform() each training set, then .transform() the corresponding test set using the mean and standard deviation of the training set.

10. How are you accounting for hemodynamic delays in the fMRI data? Note that many examples of current work use a finite impulse-response model where they horizontally stack lagged duplicates of the model features (embeddings), via Huth et al., 2016. This is a more sensitive approach, as it allows the model to estimate a linear combination of lags for each voxel / electrode in each subject.

11. Do the authors have an intuition whether this problem of autocorrelation when using shuffled splits is worse for fMRI data? My intuition is that fMRI data is by nature much more temporally autocorrelated than ECoG data. For example, I agree that it's not ideal for Mischler et al., 2024, to use shuffled splits, but with the high temporal resolution and signal properties of ECoG, it might not be as egregious as for fMRI.

12. The discussion of using shuffled splits (which I agree with the authors should basically never be used in the context of autocorrelated time series like fMRI data or natural language) could be developed into a broader point about generalization in model performance. Using cross-validation with temporally contiguous splits is certainly a good baseline recommendation, but it may be worth indicating to readers that other, even more stringent generalization tests can be theoretically interesting. For example, evaluating model generalization to entirely separate, fully held-out stimuli (e.g., Huth et al., 2016); evaluating model generalization across strictly non-overlapping sets of words (e.g., Goldstein et al., 2024); ensuring that context windows are reset at cross-validation folds (e.g., de Varda et al., 2025); and evaluating model generalization across subjects (e.g., Zada et al., 2024).

Minor comments:

Line 238: Can you summarize the paragraph starting at line 238 with a take-home message? Is the point here that this result is conceptually congruent with the paper by Kauf?

Line 343: "On the other hand, ?, Hong et al. [2024]"—missing citation (probably Antonello & Huth, 2023?)

Line 375: "contextual syntactic representation"—this doesn't seem like very standard terminology to me (e.g., many people who care a lot about syntax work on "context-free grammars"; might be worth unpacking a bit more).

In section 4.2 of the Methods, can you add a little more detail about GPT-2 XL? The main thing that occurred to me here is that I couldn't find the embedding dimensionality for XL… If I recall correctly, it's 1,600?

Line 439: "L2 penalty [Dupré la Tour et al., 2022] ??." —missing citation?

Line 451: "ane expanded" > "an expanded"

Line 630: "We note that squared error values from a model are correlated, which means that the t-test is biased towards positive results which show that an LLM contributes significant variance over a set of simpler models." I didn't follow this sentence. You mean squared error values will be correlated across cross-validation folds? Or squared error values will be correlated with model complexity?

Line 745: Hawinkel reference is missing the date.

I include many references below because I think it's critical for the authors to be comprehensive about who's done what in a piece like this.

References:

Antonello, R., Turek, J. S., Vo, V., & Huth, A. (2021). Low-dimensional structure in the space of language representations is reflected in brain responses. In M. Ranzato, A. Beygelzimer, Y. Dauphin, P. S. Liang, & J. W. Vaughan (Eds.), Advances in Neural Information Processing Systems: Vol. 34 (pp. 8332–8344). Curran Associates, Inc. https://proceedings.neurips.cc/paper/2021/hash/464074179972cbbd75a39abc6954cd12-Abstract.html

Antonello, R., Vaidya, A., & Huth, A. (2023). Scaling laws for language encoding models in fMRI. In A. Oh, T. Naumann, A. Globerson, K. Saenko, M. Hardt, & S. Levine (Eds.), Advances in Neural Information Processing Systems: Vol. 36 (pp. 21895–21907). Curran Associates, Inc. https://proceedings.neurips.cc/paper_files/paper/2023/hash/4533e4a352440a32558c1c227602c323-Abstract-Conference.html

Antonello, R., & Huth, A. (2024). Predictive coding or just feature discovery? An alternative account of why language models fit brain data. Neurobiology of Language, 5(1), 64–79. https://doi.org/10.1162/nol_a_00087

Caucheteux, C., Gramfort, A., & King, J.-R. (2021a). Disentangling syntax and semantics in the brain with deep networks. In M. Meila & T. Zhang (Eds.), Proceedings of the 38th International Conference on Machine Learning: Vol. 139 (pp. 1336–1348). PMLR. https://proceedings.mlr.press/v139/caucheteux21a.html

Caucheteux, C., Gramfort, A., & King, J.-R. (2021b). Model-based analysis of brain activity reveals the hierarchy of language in 305 subjects. In M.-F. Moens, X. Huang, L. Specia, & S. W.-T. Yih (Eds.), Findings of the Association for Computational Linguistics: EMNLP 2021 (pp. 3635–3644). Association for Computational Linguistics. https://doi.org/10.18653/v1/2021.findings-emnlp.308

Caucheteux, C., Gramfort, A., & King, J.-R. (2022). Deep language algorithms predict semantic comprehension from brain activity. Scientific Reports, 12(1), 16327. https://doi.org/10.1038/s41598-022-20460-9

Caucheteux, C., & King, J.-R. (2022). Brains and algorithms partially converge in natural language processing. Communications Biology, 5, 134. https://doi.org/10.1038/s42003-022-03036-1

Caucheteux, C., Gramfort, A., & King, J.-R. (2023). Evidence of a predictive coding hierarchy in the human brain listening to speech. Nature Human Behaviour, 7(3), 430–441. https://doi.org/10.1038/s41562-022-01516-2

Eklund, A., Nichols, T. E., & Knutsson, H. (2016). Cluster failure: why fMRI inferences for spatial extent have inflated false-positive rates. Proceedings of the National Academy of Sciences, 113(28), 7900–7905. https://doi.org/10.1073/pnas.1602413113

Goldstein, A., Grinstein-Dabush, A., Schain, M., Wang, H., Hong, Z., Aubrey, B., Nastase, S. A., Zada, Z., Ham, E., Hong, Z., Feder, A., Gazula, H., Buchnik, E., Doyle, W., Devore, S., Dugan, P., Reichart, R., Friedman, D., Brenner, M., Hassidim, A., Devinsky, O., Flinker, A., & Hasson, U. (2024). Alignment of brain embeddings and artificial contextual embeddings in natural language points to common geometric patterns. Nature Communications, 15, 2768. https://doi.org/10.1038/s41467-024-46631-y

Goldstein, A., Wang, H., Niekerken, L., Zada, Z., Aubrey, B., Sheffer, T., Nastase, S. A., Gazula, H., Schain, M., Singh, A., Rao, A., Choe, G., Kim, C., Doyle, W., Friedman, D., Devore, S., Dugan, P., Hassidim, A., Brenner, M., Matias, Y., Devinsky, O., Flinker, A., & Hasson, U. (2025). A unified acoustic-to-speech-to-language embedding space captures the neural basis of natural language processing in everyday conversations. Nature Human Behaviour, 9, 1041–1055. https://doi.org/10.1038/s41562-025-02105-9

Goldstein, A., Zada, Z., Buchnik, E., Schain, M., Price, A., Aubrey, B., Nastase, S. A., Feder, A., Emanual D., Cohen, A., Jensen, A., Gazula, H., Choe, G., Rao, A., Kim, C., Casto, C., Lora, F., Flinker, A., Devore, S., Doyle, W., Dugan, P., Friedman, D., Hassidim, A., Brenner, M., Matias, Y., Norman, K. A., Devinsky, O., & Hasson, U. (2022). Shared computational principles for language processing in humans and deep language models. Nature Neuroscience, 25, 369–380. https://doi.org/10.1038/s41593-022-01026-4

Hong, Z., Wang, K., Zada, Z., Gazula, H., Turner, D., Aubrey, B., Niekerken, L., Doyle, W., Devore, S., Dugan, P., Friedman, D., Devinsky, O., Flinker, A., Hasson, U., Nastase, S. A., & Goldstein, A. (2024). Scale matters: large language models with billions (rather than millions) of parameters better match neural representations of natural language. eLife, 13, RP101204. https://doi.org/10.7554/eLife.101204.1

Huth, A. G., De Heer, W. A., Griffiths, T. L., Theunissen, F. E., & Gallant, J. L. (2016). Natural speech reveals the semantic maps that tile human cerebral cortex. Nature, 532(7600), 453–458. https://doi.org/10.1038/nature17637

Kumar, S., Sumers, T. R., Yamakoshi, T., Goldstein, A., Hasson, U., Norman, K. A., Griffiths, T. L., Hawkins, R. D., & Nastase, S. A. (2024). Shared functional specialization in transformer-based language models and the human brain. Nature Communications, 15, 5523. https://doi.org/10.1038/s41467-024-49173-5

LeBel, A., Wagner, L., Jain, S., Adhikari-Desai, A., Gupta, B., Morgenthal, A., Tang, J., Xu, L., & Huth, A. G. (2023). A natural language fMRI dataset for voxelwise encoding models. Scientific Data, 10, 555. https://doi.org/10.1038/s41597-023-02437-z

Li, J., Bhattasali, S., Zhang, S., Franzluebbers, B., Luh, W. M., Spreng, R. N., Brennan, J. R., Yang, Y., Pallier, C., & Hale, J. (2021). Le Petit Prince: a multilingual fMRI corpus using ecological stimuli. bioRxiv. https://doi.org/10.1101/2021.10.02.462875

Mischler, G., Li, Y. A., Bickel, S., Mehta, A. D., & Mesgarani, N. (2024). Contextual feature extraction hierarchies converge in large language models and the brain. Nature Machine Intelligence, 6, 1467–1477. https://doi.org/10.1038/s42256-024-00925-4

Nastase, S. A., Liu, Y.-F., Hillman, H., Zadbood, A., Hasenfratz, L., Keshavarzian, N., Chen, J., Honey, C. J., Yeshurun, Y., Regev, M., Nguyen, M., Chang, C. H. C., Baldassano, C., Lositsky, O., Simony, E., Chow, M. A., Leong, Y. C., Brooks, P. P., Micciche, E., Choe, G., Goldstein, A., Vanderwal, T., Halchenko, Y. O., Norman, K. A., & Hasson, U. (2021). The “Narratives” fMRI dataset for evaluating models of naturalistic language comprehension. Scientific Data, 8, 250. https://doi.org/10.1038/s41597-021-01033-3

Schrimpf, M., Blank, I. A., Tuckute, G., Kauf, C., Hosseini, E. A., Kanwisher, N., ... & Fedorenko, E. (2021). The neural architecture of language: Integrative modeling converges on predictive processing. Proceedings of the National Academy of Sciences, 118(45), e2105646118. https://doi.org/10.1073/pnas.2105646118

Toneva, M., Mitchell, T. M., & Wehbe, L. (2022). Combining computational controls with natural text reveals aspects of meaning composition. Nature Computational Science, 2(11), 745–757. https://doi.org/10.1038/s43588-022-00354-6

Vul, E., Harris, C., Winkielman, P., & Pashler, H. (2009). Puzzlingly high correlations in fMRI studies of emotion, personality, and social cognition. Perspectives on Psychological Science, 4(3), 274–290. https://doi.org/10.1111/j.1745-6924.2009.01125.x

Zada, Z., Goldstein, A. Y., Michelmann, S., Simony, E., Price, A., Hasenfratz, L., Barham, E., Zadbood, A., Doyle, W., Friedman, D., Dugan, P., Melloni, L., Devore, S., Flinker, A., Devinsky, O., Nastase, S. A., & Hasson, U. (2024). A shared model-based linguistic space for transmitting our thoughts from brain to brain in natural conversations. Neuron, 112(18), 3211–3222. https://doi.org/10.1016/j.neuron.2024.06.025

Zada, Z., Nastase, S. A., Aubrey, B., Jalon, I., Michelmann, S., Wang, H., Hasenfratz, L., Doyle, W., Friedman, D., Dugan, P., Melloni, L., Devore, S., Flinker, A., Devinsky, O., Goldstein, A., & Hasson, U. (2025). The “Podcast” ECoG dataset for modeling neural activity during natural language comprehension. bioRxiv. https://doi.org/10.1101/2025.02.14.638352

Zada, Z., Nastase, S. A., Speer, S., Mwilambwe-Tshilobo, L., Tsoi, L., Burns, S., Falk, E., Hasson, U., & Tamir, D. (2025). Linguistic coupling between neural systems for speech production and comprehension during real-time dyadic conversations. bioRxiv. https://doi.org/10.1101/2025.02.14.638276

Signed: Samuel A. Nastase

(Remarks on code availability)

Version 1:

Reviewer comments:

Reviewer #1

(Remarks to the Author)
The revised paper addresses all the concerns and the new framing is appropriate. I have no further comments.

(Remarks on code availability)

Reviewer #2

(Remarks to the Author)
I thank the authors for their thorough changes to the manuscript and their hard work. The new manuscript addresses my concerns. I also read the comments of the other reviewers and they seem addressed.

Two small points:
- the authors mention in their response that there needs to be a bias correction for variance partitioning. This is more of an FYI but if one uses a method like himalaya or stacked regressions for combining feature spaces then it is less likely for the performance to be reduced when adding feature spaces. this is more of an FYI
- since the authors don't do a full variance partitioning, I think the paragraph starting line 450 is still a little bit too strong. I think a solution is to add a sentence in that paragraph saying that this can be further studied in future work (determining what language model actually bring in), and keeping it an open problem instead of a final conclusion. For example some studies like the ones that the authors did cite do find reliable variance explained (from semantics or syntax) after accounting for low level effects.

A comment about the table, which I really like: I believe that Huth et al 2016 also control for phoneme rate and Wehbe et al 2014 also control for word length in their classification.

(Remarks on code availability)

Reviewer #4

(Remarks to the Author)
I just finished a close read through the response letter and revised manuscript. I'm happy with the updated version. The tone is more constructive and scope of the critique is much more calibrated to the particular methodological choices and datasets under consideration. I like how the new text is oriented a bit more toward providing recommendations with pointers to positive examples in the literature. The new table is also a really useful addition. This makes it clear that a lot of different

groups are making slightly different choices in different publications (which is not great, but typical in a relatively new and rapidly changing subfield). I think it's very useful to survey these differences so adopters can make more informed decisions. This makes the manuscript more useful as a methodological reference for the community. I'm listing a couple very minor follow-ups below, but all of my comments have been adequately addressed and I'm happy to sign off on this one.

Figure 2: Please make very clear in the figure caption (or even in a figure title or labels) that the results in Figure 2 derive from *shuffled splits* (i.e., corresponding to the problematic method used by Schrimpf et al., 2021). There's a chance that someone who's not reading very carefully looks at this figure and assumes the whole figure corresponds to contiguous splits and interprets the figure as presenting a much broader critique of the research program than a more targeted critique of shuffled splits. Sidenote: I would move the new "Purple bars…" sentence one sentence back, since it pertains more to the left side of panel c rather than the omega bars on the right.

Similarly for Figures 5 and 6, I would add some big obvious title or label, as well as an explicit note in the figure caption, that these results correspond to contiguous train/test splits.

I want to flag Reviewer 3's comment #8 about this tension where the authors argue that positional signals and static embeddings capture much of the neural variance, but the 0th-layer GPT embeddings (which presumably contain both positional and static embedding information) do not perform particularly well. This is an insightful comment and could warrant a bit more discussion. For example, I wonder if it's really the word rate that's driving much of the PWR performance?

Thanks for pointing out that "randomly" line in Goldstein et al., 2022! I emailed the first author and he was under the impression that we were using contiguous folds as well… We'll have a look at the code just to confirm we didn't report it incorrectly in our own paper :)

I think I finally understand the way you run a statistical test on the sample-by-sample deviations in squared error between the model-predicted and actual values for the test sets. Thanks for clarifying.

The last sentence of the abstract feels a bit weird to me. I would suggest something like "Although many studies in the field do not fall victim to these missteps, our results suggest that *certain* theoretically interesting connections between LLMs and brains *can be* driven by fragile methodologies and overlooked confounds" (I don't think the qualifier "on these three neural datasets" is strictly necessary here—trying to make sure authors don't overcorrect in their concessions to reviewers :)

Page 19: "users" > "participants"?

Signed: Samuel A. Nastase

(Remarks on code availability)

Open Access This Peer Review File is licensed under a Creative Commons Attribution 4.0 International License, which permits use, sharing, adaptation, distribution and reproduction in any medium or format, as long as you give appropriate credit to the original author(s) and the source, provide a link to the Creative Commons license, and indicate if changes were

made.
In cases where reviewers are anonymous, credit should be given to 'Anonymous Referee' and the source.
The images or other third party material in this Peer Review File are included in the article's Creative Commons license, unless indicated otherwise in a credit line to the material. If material is not included in the article's Creative Commons license and your intended use is not permitted by statutory regulation or exceeds the permitted use, you will need to obtain permission directly from the copyright holder.
To view a copy of this license, visit https://creativecommons.org/licenses/by/4.0/

Changes to Framing

A core concern among reviewers was that our study makes broad, overarching claims regarding the field of LLM to brain mapping. Specifically, the concern was that our paper may be misconstrued as a general critique on LLM to brain mapping studies, rather than its intended message which is to highlight methodological concerns and lack of accounting for confounds on a very specific set of neural datasets. To address this concern, we have made significant changes to the framing of our study, which we detail below:

First, we include a comprehensive review of 35 LLM-to-brain mapping studies in Supplementary Table 1. For each study, we list whether they employ shuffled splits, the low-level confounds accounted for, which feature extraction methods were used, and the dataset type (fMRI, ECoG, or MEG). This table highlights the fact that many studies in the field use proper methods and account for confounds, and thus provides a better holistic view of the field.

New Supplementary Table 1 * Only first 8 out of 35 entries are shown here due to the length of the Table)

4 Comprehensive review of methods used in previous studies

Below, we list a large sample of studies in the LLM-to-brain mapping space. For each study, we list (a) the style of train-test split used, (b) which confounds were controlled for and how they were controlled for, (c) the activation extraction method used, and (d) the dataset type.

Paper	Split style	Low-level Confounds accounted for	Feature extraction method	Dataset type (fMRI, ECoG, MEG)
**AlKhamissi et al. (2024)** – *"Brain-Like language processing via a shallow untrained multihead attention network"*	Shuffled	Position is accounted for in a supplementary analysis (in an attempt to refute an earlier version of our paper), but appears far worse than GPT2-XL due to the authors' use of shuffled splits, which inflates GPT2-XL but not position (due to position vectors being identical between passages and hence lacking the "orthogonal" property of OASM)	Last token and mean pooling	fMRI and ECoG
**Antonello et al. (2021)** – *"Low-dimensional structure in the Space of Language Representations is Reflected in Brain Responses"*	Contiguous, a single held-out story was used for testing.	None	Sum-pooling with Lanczos downsampling	fMRI
**Antonello et al. (2023)** – *"Scaling laws for language encoding models in fMRI"*	Contiguous, a single held-out story was used for testing.	None included in the regression, but the first 100 seconds of the test set were removed to avoid ramping positional confounds observed in the signal.	Sum-pooling with Lanczos downsampling	fMRI
**Antonello & Huth (2024)** – *"Predictive coding or just feature discovery?"*	Contiguous, a single held-out story was used for testing.	None	Sum-pooling with Lanczos downsampling	fMRI
**Aw et al. (2024)** – *"Instruction-tuning aligns LLMs to the human brain"*	Shuffled	None	Last token	fMRI
**Caucheteux et al. (2021)** – *"Disentangling syntax and semantics in the brain with deep networks"*	Contiguous	Word rate, phonemes, stress and tone	Sum-pooling with Lanczos downsampling	fMRI
**Caucheteux et al. (2021)** – *"Model-based analysis reveals the hierarchy of language in 305 subjects"*	Contiguous	Phonemes (as represented in intermediate layers of GPT2)	Sum-pooling within TRs and concatenation of the resulting vectors from the last 5 TRs.	fMRI
**Caucheteux et al. (2022)** – *"Deep language algorithms predict semantic comprehension from brain activity"*	Contiguous, time series split into 5 contiguous chunks	Word rate, phone rate, stresses and tones of words of the stimuli	Sum-pooling within TRs and concatenation of the resulting vectors from the last 5 TRs.	fMRI

Second, we added a paragraph in the Introduction emphasizing that many LLM-to-brain mapping studies do not make the issues discussed in our manuscript.

Changes to Introduction

It is worth noting that many LLM-to-brain mapping studies do not exhibit these methodological issues and take care to account for potential confounds, as we summarize in Supplementary Table 1. Our study should therefore not be interpreted as a blanket critique of the field. Nonetheless, given the influence of Schrimpf et al. [2021] and its extensive follow-up work, we believe it is important to highlight these methodological missteps and offer a rigorous re-analysis of these results.

Third, we added several additional modifications to the Discussion to highlight the fact that many other studies do not have the issues we point out. Specifically, we added a paragraph emphasizing that the majority of studies in the field using other neural datasets employ contiguous splits (A). We placed greater emphasis on the fact many studies using other neural datasets do account for low-level confounds. Moreover, we note that although using multiple activation extraction methods is typically not done in these studies, this issue is less severe for electrophysiological data, where the effects of multi-token aggregation are minimal due to word-level sampling (i.e., higher resolution) of neural responses (B). We emphasized that the scope of our findings regarding the correlation between neural predictivity and next-word prediction are specific to the three neural datasets we analyzed (C). Lastly, we expanded on our first limitation to highlight the unique nature of the three neural datasets we analyzed relative to larger, more naturalistic datasets used in the field, and how this is important to consider when interpreting our results (D).

Changes to Discussion (A)

Although the majority of language encoding studies using these three neural datasets have employed shuffled splits, the use of shuffled splits is not pervasive in studies using other neural datasets. After conducting a literature review of 35 language encoding studies (Supplementary Table 1), we found that only 2 studies using other neural datasets shuffled: Mischler et al. [2024] and Goldstein et al. [2022]. Both these studies analyze electrophysiological signals, where the impact of shuffling is less pronounced (though still non-negligible). Moreover, Goldstein et al. [2022] focuses its analyses on *GloVe*, and we observed virtually no difference in the neural predictivity of static word embeddings models on *Fedorenko2016* when switching from shuffled to contiguous splits, likely due to the low temporal mixing rate of electrophysiological signals (relative to fMRI) and word embedding models (relative to LLMs). In sum, we emphasize that although the negative effects of shuffled splits might be less egregious in some circumstances, they are generally problematic and should be avoided.

Changes to Discussion (B)

In addition, an important finding in our study is After addressing methodological concerns, we find that simple confounds, namely , positional signals and word rate, play an important role in account for the vast majority of LLM-to-brain mappings , even when using contiguous splits on these neural datasets. Most studies do not include simple confounds in the regression that maps LLM representations to neural activity, although few studies did account for such confounds. in these datasets. Although these confounds were not accounted for in other studies using these three neural datasets, several studies using different datasets have accounted for confounds. Huth et al. [2016] included phonemes, phoneme rate and word rate as confound regressors, and Kumar et al. [2024] included these regressors along with a silence indicator using banded ridge regression. Caucheteux et al. [2021] controlled for word rate and phonological features before examining how much of the encoding performance of LLMs can be explained by syntax vs. semantics. Reddy and Wehbe [2021] iteratively accounted for simpler features, such as punctuation, before analyzing the neural predictivity of more complex syntactic models and LLM models. LeBel et al. [2021] and de Heer et al. [2017] also iteratively accounted for the neural predictivity of simpler acoustic and phonetic features before introducing more complex, semantic models. Our findings highlight the importance of the continued use of these low-level confounding predictors, especially when scientists seek to draw inferences about higher-level representations in the brain based ondifferences in neural predictivity between models. Zada et al. [2025a] include lower-level acoustic, phonetic, syntactic, and non-contextual word embedding features in their ECoG dataset. Zada et al. [2025b] further include a confound regressor for the on/off task structure of dyadic conversations. We provide a comprehensive review of the many ways in which language encoding studies have accounted for confounds in Supplementary Table 1.

Our findings also underscore the importance of systematically investigating multiple activation extraction methods from LLMs when fitting encoding models ~~to fMRI datasets. In *Pereira2018*,~~ in fMRI datasets, where each neural sample corresponds to multiple tokens (words or sub-words). For instance, in *Pereira2018* we observe that the most commonly used approach, last-token extraction, generally performs the worst and particularly disadvantages bidirectional transformers ~~. Differences between extraction methods do not only affect analyses that compare different LLMs (and other computational models). For example,~~ Jain et al. [2020] (Figure 3b). The choice of activation extraction method is less relevant for electrophysiological datasets due to the high temporal resolution. In the case of *Fedorenko2016*, the activation extraction method only differs in effect for words that are split into multiple tokens, and has a negligible impact on neural predictivity (Figure 3b). Based on our literature review, we found that only 2 fMRI studies discussed the impact of activation extraction methods in their study: Kauf et al. [2024] and Jain et al. [2020]. Kauf et al. [2024] showed that their results were similar when using either mean or last token pooling. Jain et al. [2020] demonstrated that the standard activation extraction method for narrative comprehension datasets (analogous to sum-pooling) can lead to artifacts~~where LLM~~. Specifically, artificial neural network units with the longest timescales of context integration ~~are~~ were mapped to brain regions known to have short integration timescales, such as primary auditory cortex. More broadly, our study along with that of Jain et al. [2020] highlights a fundamental concern when interpreting LLM-to-brain mappings: the associations between model representations and brain regions are influenced by the assumptions inherent in the chosen activation extraction method.

Changes to Discussion (C)

Returning to the larger picture, our study provides an updated view regarding the ~~"~~extent to which the "brain-likeness~~"~~ ~~of LLMs . Arguably the biggest claim about convergence between deep learning models and brains in Schrimpf et al. [2021] was that the models that best predict brain responses are precisely~~" of LLMs is apparent in the datasets of Schrimpf et al. [2021]

. Our conclusions are scoped to the three neural datasets analyzed in Schrimpf et al. [2021] (*Pereira2018*, *Fedorenko2016*, *Blank2014*) and to a standardized pipeline (contiguous splits, appropriate pooling of model activations, and L2-regularized regression). On these datasets, the central claim of Schrimpf et al. [2021]—that models with better next-word prediction are those that best predict ~~the next word. Here, we find that this central finding is not robust, and depends on choice of neural datasets, models, and activation extraction methods. Results in the literature are similarly mixed . For instance, Pasquiou et al. [2022] reported~~ brain responses—does not hold under valid methodological choices. We stress that this does *not* imply a field-general failure. Outside the Schrimpf et al. [2021] datasets, the literature is somewhat mixed on the next-word prediction correlation, though leaning positive: Pasquiou et al. [2022] report a correlation between perplexity and neural predictivity ~~within, but not across, model classes.Caucheteux and King [2022] trained 36 transformer models from scratch and found~~ *within* but not *across* model classes, and Caucheteux and King [2022] show that although trained ~~models exhibited higher neural predictivity than untrained models~~transformers outperform untrained ones, neural predictivity ~~ceased scaling with~~ can decouple from next-word prediction ~~at some point during model training . On the other hand, ?, Hong et al. [2024] and Bonnasse-Gahot and Pallier [2024] did report significant correlations between neural predictivity and next-word prediction. Altogether,~~ as training proceeds. By contrast, studies with larger samples and longer, more naturalistic narratives often report positive correlations across pretrained models [Antonello and Huth, 2024, Hong et al., 2024, Bonn . Our analyses do not adjudicate those settings; rather, they clarify that, in the datasets used by Schrimpf et al. [2021], these results do not hold. Moreover, this can be due to a variety of reasons that are beyond the specific methodological correctives we address, such as the contrived structure of the ~~appearance of this correlation seems to depend somewhat unpredictably on the choice of dataset, models, and methods.~~ Fedorenko2016 and Pereira2018 datasets, the relatively unengaging nature of the stories in the Blank2014 datasets, or even the specific method that Schrimpf et al. [2021] uses for operationalizing

Changes to Discussion (D)

There are ~~three~~ six important limitations to consider when interpreting the results of our study. First ~~, the three datasets used in our study are somewhat limited in scope, as participants either~~ and foremost, all our results are conducted using neural datasets in which participants read short passages, read isolated sentences, or listened to ~~non-naturalistic stories~~ short stories which are designed to have complex, low-frequency syntactic structures. By contrast, many recent language encoding studies have ~~shifted towards using larger, more naturalistic datasets, such as those released by Nastase et al. [2021] and LeBel et al. [2023]. Our results do not directly address these newer datasets , and we encourage future work to evaluate methodological robustness and potential confounds on larger, naturalistic datasets~~ participants listen to engaging stories and collect much more data per participant, as in LeBel et al. [2021], or contain data from many more participants, as in Nastase et al. [2021]. The unique nature of the datasets combined with the fact these datasets contain less data relative to more recent datasets is important to consider when interpreting our results. Second, although we ~~applied~~ used ridge and

Finally, we updated our study title to include the qualifier "can" and updated our abstract to be more specific that our results pertain to the three specific neural datasets we use and also to include that many studies do not make these methodological missteps.

Changes to Title and Abstract

Illusions of ~~Alignment Between Large Language Models and Brains Emerge From Fragile Methods and Overlooked Confounds~~ alignment between large language models and brains can emerge from fragile methods and overlooked confounds

Emerging research seeks to draw neuroscientific insights from the neural predictivity of large language models (LLMs). However, as results continue to be generated at a rapid pace, there is a growing need for large-scale assessments of their robustness. Here, we analyze a wide range of models ~~, methodological approaches , and~~ and methodological approaches across three widely used neural datasets. We find that some methodological approaches, particularly the use of shuffled train-test splits, have led to many impactful yet unreliable findings, and that the method by which activations are extracted from LLMs can bias results to favor particular model classes. Moreover, we find that confounding variables, particularly positional signals and word rate, perform competitively with trained LLMs and fully account for the neural predictivity of untrained LLMs. ~~In summary~~Although many studies in the field do not fall victim to these missteps, our results suggest that theoretically interesting connections between LLMs and brains on these three neural datasets are driven largely by fragile methodologies and overlooked confounds.

We believe that with these modifications, the message of our manuscript is much more precise to the specific studies we are re-evaluating.

Reviewer #1

This paper presents a careful re-evaluation of claims regarding the alignment between large language models (LLMs) and neural responses. The authors demonstrate that certain analytic choices, particularly the use of shuffled train-test splits and specific activation extraction methods can lead to inflated or misleading estimates of neural predictivity. They introduce useful baselines (e.g., the Orthogonal Autocorrelated Sequences Model) and show that simpler confounds such as position and word rate can explain much of the observed variance.

While it is a valuable contribution, there are significant concerns about the tone and framing of the paper, particularly the title and overarching claims. The title suggests that the entire field of model-to-brain alignment is illusory, but the authors' own results do not support this level of generality. In fact, their results show that when methodological issues are addressed, i.e., with contiguous splits, appropriate regularization, and validated baselines, LLMs still predict neural data to a meaningful degree. In that case, it is not appropriate to call it an “illusion,” as overstating risks undermining a field that is still evolving. If the goal is to correct methodological flaws in a few highly cited papers, largely from a small number of labs, that message should be delivered more precisely, as much of LLM-brain alignment work uses the very practices the authors endorse.

Thank you for raising these points. We have made several modifications to the manuscript to ensure that the message is delivered more precisely, and avoids making broad, overarching claims about the entire field. These modifications are detailed in the Changes to Framing section above.

The authors argue that next-word prediction is not robustly correlated with neural predictivity, but this conclusion seems too strong. As they themselves note, the effect of model family is large, and within certain families (e.g., unidirectional transformers), there is a clear scaling trend in Figure 3 consistent with earlier work (e.g., Pasquiou et al., 2022). While the across-family correlation is fragile, this within-family effect is non-trivial and deserves a more careful discussion. The authors mention this in passing but do not highlight that they essentially replicate that earlier trend.

We thank the reviewer for pointing this out. Based on these comments, we have updated our manuscript to include correlations between neural predictivity and next word prediction ability within model classes when using contiguous splits. Specifically, for the unidirectional transformers included in

our next-word prediction analyses (openai-gpt, distilgpt2, gpt2, gpt2-medium, gpt2-large, and gpt2-xl), we do not find a significant correlation between neural predictivity and negative perplexity except for in one case: the Pereira2018 dataset with last token feature extraction. Moreover, last token feature extraction consistently resulted in the lowest neural predictivity values relative to mean and sum pooling (Figure 3b). Hence, the evidence does not support a robust correlation even within just the unidirectional transformers used by Schrimpf et al. 2021. Among bidirectional transformers (including all of the models labeled as such in Figure 3a), we do not find a significant correlation between neural predictivity and negative perplexity on any dataset with any feature extraction method.

Changes to Results

One concern might be that correlations exist within individual model classes (bidirectional or unidirectional) but are masked when computed across both classes simultaneously. However, even when analyzing within each model type separately, we still find minimal evidence for correlations between next-word prediction and neural predictivity when using contiguous splits. Among bidirectional models, no significant correlations emerge regardless of dataset or feature extraction method.

For unidirectional models, only one significant correlation was observed ($p = 0.022$), specifically when using the *Pereira2018* dataset with last-token feature extraction.

We do however note that these failed replications are restricted to the datasets evaluated in Schrimpf et al. 2021, and other groups have found the correlation with next-word prediction on other datasets. We now discuss this in greater detail in our discussion, which is detailed in the Changes to Framing section, specifically in Changes to Discussion (C).

- Furthermore, the paper does not specify how perplexity was computed. Was it on a standard benchmark, or on the same stimulus texts used in the neural datasets? This omission is important, especially given the short stimuli used in some datasets (e.g., Pereira2018, Fedorenko2016). Clarifying this would strengthen the argument.

Thank you for pointing this out, we have included an explanation of how perplexity was computed in Methods Section 4.6. Importantly, we used the exact same perplexity scores reported by Schrimpf et al. 2021, which are not computed on the stimuli in the neural datasets but rather using a popular natural language processing corpus (WikiText-2)

Changes to Methods

4.6 Next-word prediction metric

For our next-word prediction analyses, we use the exact same perplexity scores reported by Schrimpf et al. [2021]. In that work, models were evaluated on the WikiText-2 corpus (2M / 218k / 246k tokens in the train / validation / test splits) [Merity et al., 2016]. Each model tokenized the text with its native tokenizer (vocabulary capped at 250k) and processed it in contiguous 32-token blocks. For every block the final-layer hidden state (penultimate, if the model contained a classifier head) was passed to a single linear read-out trained to predict the next token among the 50k most frequent vocabulary items using cross-entropy loss, AdamW optimization (learning rate $= 5 \times 10^{-5}$, mini batches = 4 blocks) and early stopping on validation loss; only the linear read-out's weights were updated. Predictive performance was quantified as perplexity on the held-out test set. We do **not** recompute these scores: all perplexity values used here are taken directly from Schrimpf et al. [2021] so that our analyses are anchored to the identical scores originally linked to neural predictivity.

- Additionally, one should be cautious against plotting perplexity across unidirectional and bidirectional models in the same space. These architectures are trained with different objectives (causal vs. masked language modeling), and bidirectional models are inherently out-of-domain for next-word prediction tasks. Their higher perplexity is not necessarily indicative of weaker language modeling but of task mismatch. This conflation likely dominates the pattern in Figure 4, and the same issue was

present in Schrimpf et al. (2021). The authors could be more explicit in acknowledging and adjusting for this discrepancy.

It is important to mention that the goal of Figure 4 was not to examine if general language capabilities correlate with neural predictivity. If this were the case, we completely agree that using perplexity for bidirectional models is ill-suited. However, the goal of Figure 4 was to respond to the claim from Schrimpf et al 2021 that as models do better specifically at next-word prediction, they predict brain responses better. Schrimpf et al 2021 use this as evidence that human brains are optimizing a predictive processing objective. Given this goal, we believe that using perplexity for bidirectional models was justified. Furthermore, the dominant pattern in Figure 3 is that all contextual models show virtually the same performance (for the Pereira2018 and Fedorenko2016 datasets; we see more variation in Blank2014, but all models are outperformed by a trivial positional signal). Therefore, it is unlikely that using an alternative metric for bidirectional models would result in a significant correlation with neural predictivity, at least not on these datasets. However, we agree that there are important limitations associated with using perplexity to evaluate bidirectional models, and we acknowledge these issues in the Supplementary Discussion Section.

<u>Changes to Supplementary Discussion</u>

While perplexity is a standard metric for evaluating unidirectional language models, it is not necessarily well-suited for bidirectional models. By design, bidirectional architectures such as BERT are not trained to perform next-word prediction, but rather to fill in masked tokens using both left and right context. As a result, applying perplexity—which requires autoregressive prediction—places these models in a setting misaligned with their training objective. Consequently, perplexity may underestimate the representational quality of bidirectional models and should be interpreted with caution when comparing them directly to unidirectional models.

- The authors justifiably adopt L2-regularized regression, but it's worth noting that the performance of larger models like GPT2-XL may still suffer due to overparameterization relative to the size of the datasets. This is particularly important for fMRI datasets with limited samples. Regularization can mitigate but not eliminate this challenge, and the authors might consider discussing this limitation more explicitly, especially when comparing large and small models.

We agree, and we have expanded upon our limitations to highlight this point.

<u>Changes to Discussion</u>

Second, although we ~~applied~~ used ridge and banded ridge regression to mitigate ~~cases where stacking models together (e.g.~~ overfitting when using large models (e.g. ~~*PWR+GloVe+*~~*GPT2XL*) ~~decreases neural predictivity relative to a subset of those models~~or model combinations (e.g. *PWR+GloVe+GPTXL*), our procedure ~~might~~ may still be biased ~~against regressions with many~~ in these cases relative to when using simpler models due to noise in neural data and low sample sizes. ~~Finally,~~ This issue may disadvantage the neural predictivity of LLMs relative to simple models, as well disadvantage larger LLMs relative to smaller LLMs. Third, our Ω metric can become unstable when the neural variance that an LLM explains is near 0

Minor Points
• Missing references on lines 343 and 539

We have ensured that all references are present in the updated manuscript.

• The writing is clear overall, though some claims could be tempered to better reflect the of the results.

We have tempered the language in response to your earlier comments. Thank you for your helpful review!

Reviewer #2

This paper addresses an important reproducibility issue pertaining to aligning brain activity and LLM representations (though really this affects any type of representation). The authors show that some recent results, specifically those of Schrimpf et al. 2021, are not robust. One aspect is due to the use of shuffled splits when evaluating models, which inflate significance due to the autocorrelation in time, and the other is due to the effect of low level features which could explain most of the predictivity observed by LLMs. After adjusting for some of these effects, the authors find that even the claim that models with better perplexity are better predictors of brain activity does not replicate.

Honestly, it is surprising that shuffled train-test splits were used at all in the studies mentioned. This problem has already been identified in work prior to 2021, some of which cited by the authors. fMRI is notoriously temporally autocorrelated. One solution if one insists on using shuffled train-test splits is to throw buffers of data (for example 20 seconds) on each end of the splits. The problem is that this throws away a lot of data especially in limited datasets. This detail (throwing a buffer away) could be a small addition to the discussion section for completeness. TODO

We agree that throwing a buffer away around each side of a chunk can be used in a shuffled splits configuration, though this has the major downside of losing (i.e., throwing out) a lot of data. We now include this in a paragraph in our discussion addressing various forms of contiguous train-test splits.

Changes to Discussion

~~In addition to commenting on~~ Beyond just using contiguous splits, there are several ways analyses can be made more stringent. First, one can evaluate on entirely held-out stimuli (e.g., whole stories never seen in any train or validation fold), as in Huth et al. [2016]. Second, one can enforce strictly non-overlapping vocabularies between train and test (e.g., Goldstein et al. [2024]), which is especially stringent for word-level/ECoG settings and helps diagnose lexical memorization versus compositional generalization. Third, resetting model context windows at cross-validation boundaries (e.g., de Varda et al) prevents inadvertent context leakage when folds split within long stimuli; this is most critical when the same narrative continues across folds. Finally, cross-subject generalization (e.g., Zada et al. [2024]) tests whether mappings learned on some individuals transfer to unseen individuals, though it introduces variability from inter-subject alignment. We view temporally contiguous cross-validation as a baseline, and these stricter regimes as complementary stress tests whose adoption should be matched to the stimulus structure and analysis goals. If one insists on using shuffled splits, ~~our findings~~ one can insert temporal buffer zones (e.g., ± 20 s) around train–test boundaries to reduce autocorrelation leakage; however, this approach discards substantial amounts of already limited data and is generally inferior to simply using temporally contiguous splits.

The results with OASM are very compelling, and so are the effects of different ways to extract features and the way that low level features are tested. However, I think there is an important improvement to be made:

A check, in lines 627-629, the N for "this test" corresponds to the number of time points that were tested? You can't do a t-test on correlated time points (actually, isn't the entire point of the paper that time points are correlated?). One way to do this test is to block the time course of errors into large blocks that are slower than fMRI (e.g. 20 sec), and then use a block permutation test to show that the errors of M+LLM is different from M

Yes, your interpretation is correct. The *N* for the tests described in lines 627-629 is the number of time points in the dataset, and indeed, much of (though not entirely) the point of our paper is that such time points are correlated (i.e. not independent samples).

In order to address this concern, we reproduced all our main statistical results with *GPT2XL* and *GPT2XLU* using a blocked paired t-test. In order to perform a blocked paired t-test, we averaged squared error values within each passage (*Pereira2018)*, sentence (*Fedorenko2016)*, and within each group of consecutive 10 TRs (which corresponds to 20 seconds, *Blank2014)*. Averaging within each passage/sentence/non-overlapping TR window mitigates the non-independence issue. These results are displayed in Extended Data Figure 7, and referenced throughout the results section. In summary, our main claims do not change with the blocked paired t-test.

Importantly, the sample size after averaging was still large enough to assume normality for each neural dataset, with the minimum sample size being 52. With enough samples, we elected to use a blocked paired t-test rather than a blocked permutation test.

We would also like to note that the reason we show results with a "non-blocked" paired t-test throughout the study is based on the following logic. Our null hypothesis was that simple models (e.g., OASM, positional signals, word rate, GloVe) account for the neural variance that a LLM explains. The fact that brain responses are correlated in time inflates the probability of Type 1 error, in other words it inflates the probability that the statistical test indicates that LLM explains significant neural variance over the simple models when it actually does not.

Since a central message of our study is that simple models account for a large fraction of the neural variance LLMs explained on these neural datasets, the inflation of Type 1 error actually makes our claims more conservative. We discussed this in the "Statistical testing" section from the Online methods, and have added a note in the main results section to state this clearly.

Changes to Results

ance than *OASM* alone. Squared error values are computed by subtracting the predicted brain responses from the actual responses and squaring the result (4.14). Squared error values are not independent within a given passage, sentence, or consecutive TRs, which violates the assumptions of the t-test but biases results in favor of *GPT2XL* (see 4.14 for further explanation). As an additional check, we also report results with a blocked paired t-test, where the t-test was applied after averaging squared error values within each passage (*Pereira2018*), sentence (*Fedorenko2016*), or non-overlapping consecutive

... continued on next page in the manuscript

TRs (*Blank2014*) to mitigate non-independence (4.14). We applied FDR correction for each participant separately ~~for this analysis [Benjamini and Hochberg, 1995](4.14)~~[Benjamini and Hochberg, 1995].

Changes to Methods

To ensure findings using the paired t-test were robust, we replicated the analyses relating to *GPT2XL* and *GPT2XLU* using a blocked paired t-test. To perform the blocked paired t-test, we averaged squared error values within each passage for *Pereira2018*, each sentence for *Fedorenko2016*, and within non-overlapping 10 TR (20 sec) segments *Blank2014* (Extended Data Figure 7). Averaging mitigates non-independence since squared error values are most correlated within a given passage, sentence, or consecutive TRs. Sample sizes after averaging were still sufficiently large for the normality assumption: $N = 96$ for voxels in *Pereira2018* with only data from *EXP2*, $N = 72$ for voxels in *Pereira2018* with only data in *EXP3*, $N = 168$ for voxels with data in both experiments, $N = 52$ for *Fedorenko2016*, and $N = 138$ for *Blank2014*. Given that normality assumptions were met, we elected to use the a blocked t-test rather than a blocked permutation test.

Changes to Extended Data

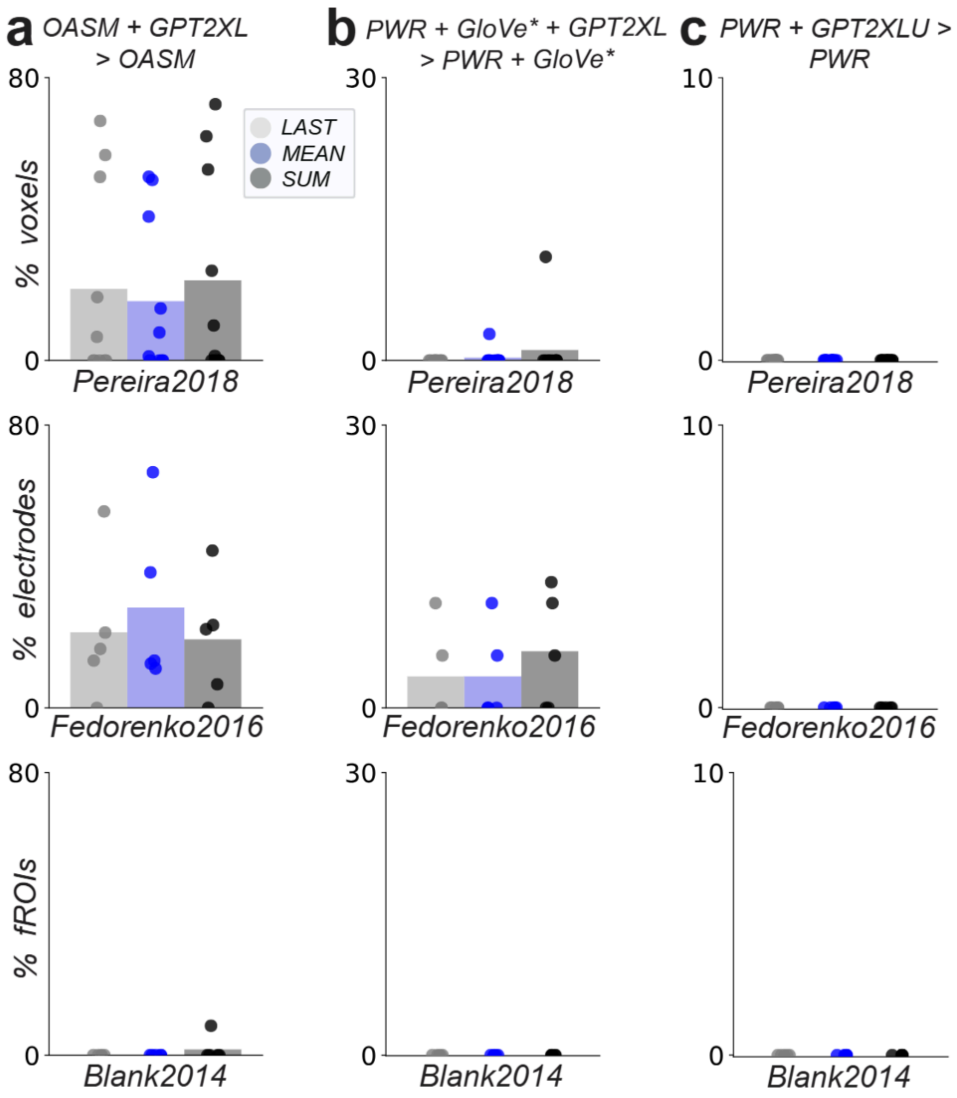

Figure 7: Reproduction of main results obtained from using the paired t-test, except t-test was applied after averaging squared error values within passages for *Pereira2018*, within sentences for *Fedorenko2016*, and for every 10 TRs within each story for *Blank2014*. Color legend indicates activation extraction method used for *GPT2XL/GPT2XLU*. **a)** Same as Figure 2**d** with modified statistical procedure. **b)** Same as Figure 5**e** with modified statistical procedure. * symbol above *GloVe* indicates that *GloVe* was only used for *Pereira2018*. **c)** Same as Figure 6**e** with modified statistical procedure.

- An additional thing to add would be to illustrate the different components of the variance partitioning shared, unique etc) on the brain.

We assume the reviewer is asking for a breakdown of how much each component of the simple model accounts for the neural variance LLMs explain. For instance, how much of the neural variance of *GPT2XL* in Pereira2018 do positional signals, word rate, and GloVe each individually explain?

In order to evaluate each component of the simple model, we would need to apply bias correction to account for the fact that adding a component can sometimes reduce the variance explained when evaluating on a held-out test set. Such bias correction applied to the simple model introduces methodological complexity, and we preferred the simplicity of evaluating LLMs against a minimal,

unified model. As such, after much consideration, we decided not to quantify unique and shared contributions of different components, because we treat each simple model as a unified baseline. We only applied bias correction in our study to benefit the LLMs by ensuring that adding the LLM onto the simple model never performs worse than the LLM alone (Section 4.13).

Although we do not show a breakdown of the variance partitioning metrics across components, we visualize the neural predictivity of positional signals, word rate, and GloVe individually for each sub-experiment in Pereira2018 in Extended Data Figure 1a. This figure provides a good intuition for how important each component of the simple model is in accounting for the neural variance that GPT2XL explains. We accidently referenced Extended Data Figure 1a as Extended Data Figure 2a in the Discussion, and have corrected this mistake in the updated draft as well as referenced Extended Data Figure 1a in Section 2.3 as well.

Are some of the differences in the Fedorenko 2016 results due to the fact that it's electrophysiology and thus the data has a faster mixing rate / is autocorrelated with a shorter lag? This should be discussed in the relevant result sections.

Thank you for bringing this up. There are two main differences that we see between Fedorenko2016 and the fMRI datasets. First, the problem with shuffling is not as extreme as in the fMRI datasets: OASM explains comparatively less of what GPT2XL explains on the Fedorenko2016 dataset (~50%) than on Pereira2018 (~85%) or Blank2014 (~100%), and the across-layer performance trend is not as affected by the use of shuffled splits in Fedorenko2016 (whereas it is highly anticorrelated in the Pereira2018 and Blank2014 datasets). We agree that this likely reflects the fact that electrophysiology is autocorrelated with a shorter lag. Second, the effect of activation extraction choice is negligible in Fedorenko2016, though it is considerably larger on the fMRI datasets. This makes sense, as the choice of activation extraction only matters when multiple tokens occur between individual recording timepoints. While this is always the case for fMRI, often with many tokens between individual acquisitions, it is typically not the case for ECoG, where recordings are fast enough that multiple samples are taken for each word (and can be averaged). This is the case for the Fedorenko2016 (and other ECoG datasets, e.g. the recent Podcast dataset (Zada et al. 2025)): although some minor differences occur between the feature extraction choices due to multi-token words, the variability in performance between different feature extraction methods is much smaller than what is seen in the fMRI datasets. We now address these points explicitly in our discussion.

Changes to Discussion

On *Fedorenko2016, OASM* accounts for a smaller portion of the neural variance LLMs explain and the across layer patterns are more similar between shuffled and contiguous splits relative to the two fMRI datasets. Therefore, the impact of shuffled splits is more severe on the two fMRI datasets relative to electrophysiological dataset used here.

~~For example, Jain et al. [2020]~~ (Figure 3b). The choice of activation extraction method is less relevant for electrophysiological datasets due to the high temporal resolution. In the case of *Fedorenko2016*, the activation extraction method only differs in effect for words that are split into multiple tokens, and has a negligible impact on neural predictivity (Figure 3b). Based

Can the authors comment on focusing on the layer that achieves the best neural predictivity? The way it is stated it sounds like it might be double dipping (line 69 and especially lines 617 5o 629).

We agree that selecting the best LLM layer or tuning hyperparameters on the test set constitutes double dipping and is not ideal. We initially followed this approach because Schrimpf et al. (2021) and many studies mapping LLMs to brain data adopt this practice. To verify that it did not meaningfully affect our results, we re-ran our analyses using the validation set for model selection. Specifically,

within each outer fold of nested cross-validation, we used the validation set to choose the best layer or hyperparameters, then pooled predictions across folds to compute out-of-sample R2. We implemented this procedure for GPT2-XL as well as the two simple models that required hyperparameter selection, OASM and positional signals. The table below, which is included in our study as Supplementary Table 2, compares results obtained using the validation set versus the test set for selection. The performance difference between GPT2-XL and OASM in the shuffled case and GPT2-XL and positional signals in the contiguous case is highly similar regardless of whether we use the validation or test set for selection. Furthermore, although performance is lower when using the validation set for selection as expected, the decrease in performance relative to when using the test set is relatively small. In summary, while we agree using the validation set for selection is ideal, we believe the decision to use the test for selection does not impact our findings. We have also mentioned this fact in the Discussion as an additional limitation.

Supplementary Table 2

Model Name	Split Type	Selection using Val	Selection using Test	Dataset
GPT2-XL	Shuffled	0.1540	0.1540	Pereira2018
OASM	Shuffled	0.2147	0.2157	Pereira2018
GPT2-XL	Contiguous	0.0215	0.0232	Pereira2018
Position	Contiguous	0.0092	0.0097	Pereira2018
GPT2-XL	Shuffled	0.0836	0.0855	Fedorenko2016
OASM	Shuffled	0.0969	0.0979	Fedorenko2016
GPT2-XL	Contiguous	0.0489	0.0509	Fedorenko2016
Position	Contiguous	0.0465	0.0471	Fedorenko2016
GPT2-XL	Shuffled	0.0017	0.0029	Blank2014
OASM	Shuffled	0.2747	0.2750	Blank2014
GPT2-XL	Contiguous	0.0001	0.0004	Blank2014
Position	Contiguous	0.0069	0.0085	Blank2014

Changes in Discussion

~~research to validate and extend our findings~~Finally, as is standard in the field [Schrimpf et al., 2021, Antonello et al., 2023], we selected the best LLM layer based on test set performance. For consistency and to ensure a fair comparison, we also selected hyperparameters for the simple models (*OASM* and positional signals) using the test set, with the number of hyperparameter choices matched to the number of *GPT2XL* layers. Although using the test set for hyperparameter selection

15

is not ideal, our results are nearly identical when using the validation set for hyperparameter selection (Supplementary Table 2).

Finally, while the paper is an important contribution, it might be misconstrued by too hasty a reader to mean that all reports an alignment between LLMs and brains are illusory. The title could be perhaps made more clear, or alternatively a line can be added to the abstract clarifying that these limitations do not affect all studies, and that some studies do control for low level properties like positional signals and word rate.

Thank you for raising the points. We have made several modifications to the manuscript to ensure that the message is delivered more precisely, and avoids making broad, overarching claims about the entire field. These modifications are detailed in the Changes to Framing section.

Anyways I think that this paper addresses an important caveat of many recent studies. Results of small modifications to a model without controls are over-interpreted. In any field of science, one experiment should not lead to a convincing answer. It is the robustness of results across studies that should lead to a trusted hypothesis.

Thank you for the positive comments!

Tiny typos: line 59: acquired after the presentation. Line 343 ?

We have fixed both these issues in the updated manuscript.

Reviewer #3

Summary

An influential finding in the field of cognitive neuroscience is that one can process text word-by-word with AI models (or large language models --LLMs) and use the resulting LLM-based representations of text as predictors of human brain activity recorded while participants listened or read the same text. It turns out that one can predict the brain data quite well (up to some data-inherent noise ceiling) on the basis of LLM representations. A common big-picture interpretation is that the way LLMs process text is "brain-like", which can be viewed as intriguing given that contemporary LLMs are primarily collections of clever engineering tricks for practical applications rather than biologically-inspired or cognitively-inspired models.

One of the more influential studies that showed this phenomenon across a range of LLMs and brain datasets is the study by Schrimpf et al, (2022), which inspired decent number of follow-up studies. The authors in the submitted manuscript challenge the main findings in the original work on methodological grounds -- a specific choice of how the test datasets to evaluate neural predictivity were constructed was suboptimal given known properties of brain data.

Specifically, the brain signals are temporally autocorrelated, meaning that the data samples recorded nearby in time tend to be similar. The original work allowed the data in the training set and the testing sets to come from the temporally adjacent parts of the dataset by randomly partitioning the datasets into train/test splits ("shuffled splits"). The concern arises that the reported, and influential, high neural predictivity scores were achieved because the mapping between LLMs and brains exploited this spurious feature: similarity of train and test data points due to temporal autocorrelation, rather than genuine LLM brain-likeness.

The authors of the submitted manuscript re-analyzed the datasets by Schrimpf et al (2022) with (mostly) the same LLMs but this time not allowing training and testing data points to be collected from nearby in timepoints. Test dataset came from separate passages different that the ones in the training set ("contiguous splits" approach). It turns out that neural predictivity evaluated this way is indeed much lower than originally reporte reported neural predictivity correlation by constructing a model that only predicts brain data based on temporal adjacency --- it turns out the model fits as well or better than LLMs. This methodological flaw then propagates through other findings in the study.

All in all, this study an impressive body of work and a welcome addition to the literature and a contribution to the community. The rationale is sound, the results are convincing (and robust over the choice of metrics and different models), and I find the interpretation is justified and constructive given the reported data. I have no problems seeing this published. I do have some clarification requests which I would love to see addressed before I can recommend publication. I think authors should not have trouble addressing this in revision.

Thank you for the positive feedback on our study!

Major

1. State deviations in methods and results from Schrimpf et al. (2022) explicitly. On l. 90 you state "using largely the same methodological choices implemented in Schrimpf et al." It would be useful to briefly state whether any important deviations from Schrimpf et al. were made (main text or in online methods). Similarly, please consider indicating, at least when reporting the first batch of results in Figure 1 whether your LLM predictivity scores on shuffled splits are in the same ballpark as reported by Schrimpf et al (i.e. whether you replicated their success). Your main point stands regardless, but this would be welcome for completeness.

Thank you for this comment. We now include an additional section in the Methods titled "Deviations from Schrimpf et al. 2021", where we enumerate all methodological deviations.

Changes to Methods

4.12 Deviations from Schrimpf et al. [2021]

We make several methodological deviations from Schrimpf et al. [2021] as noted throughout the paper. Here, we list them explicitly.

1. We use a larger number of folds for each dataset (8 for EXP1 in *Pereira2018*, 6 for EXP2 in *Pereira2018*, 13 for *Fedorenko2016*, and 8 for *Blank2014*). On the other hand, Schrimpf et al. [2021] uses 5-fold cross-validation for all datasets.
2. We use L2-regularized (ridge) regression instead of ordinary least squares regression (except where noted in Figure 1)
3. We use three feature extraction methods: last token, mean-pooling, and sum-pooling. We report the results for each, and emphasize the best performing method for each model. By contrast Schrimpf et al. [2021] only used last token pooling.
4. We use the RWKV-4 series of models as representative RNN language models. By contrast, Schrimpf et al. [2021] used two RNN-based models: *Skip-Thoughts* and *LSTM-LM1B*.
5. In the *Pereira2018* analyses, Schrimpf et al. [2021] accidentally included sentences from the same passage as well as category as context when inputting a given sentence into a language model. We correct for this, and only include the preceding sentences from a given passage as context.

The neural predictivity scores we see in the shuffled setting are indeed very similar to what was seen by Schrimpf et al. 2021. We have added a sentence in the Results Section for Figure 1 to specify this.

Changes to Results

We compared the neural predictivity of *OASM* to that of *GPT2XL* when using largely the same methodological choices implemented in Schrimpf et al. [2021]. These methodological choices were fitting the mapping between model activations and brain responses with ordinary least squares (OLS) regression (4.8) and extracting activations from *GPT2XL* using the last token of the stimuli, which we term *GPT2XL-LAST* (4.3). The neural predictivity of *GPT2XL-LAST* with OLS regression is similar to what was reported in Schrimpf et al. [2021] before noise ceiling correction. We performed a one-sided Wilcoxon signed-rank test across participants to determine if *GPT2XL* neural predictivity was significantly higher than that of *OASM* with $\alpha = 0.05$ (4.15). Under these settings, *OASM* predicted brain responses qualitatively on-par with or better than *GPT2XL-LAST* across all three datasets, and there was no case where *GPT2XL-LAST* predicted brain responses significantly better than *OASM* (Figure 1a, top left panel).

2. "The activations of OASM are orthogonal for distinct passages". I think spending a line our two adding more implementation details would be welcome (and refer to the online methods for the rest). Or consider adding a visualization that shows (here or in the online methods) example "activations" in OASM across say 3-4 passages? The concise description of OASM design is already pretty good, but I think it that would help readers getting additional intuition for OASM and the problem it addresses.

Thank you for this comment. We now provide a short description of OASM's implementation details in the Results (Section 2.1.1) to provide additional intuition regarding the problem it addresses.

Changes to Results

Shuffled splits are known to be problematic in the field of language neural encoding because brain responses are temporally autocorrelated for reasons that are not entirely stimulus-driven [Zada et al., 2024]. Due to temporal autocorrelation in brain responses, a model can achieve high neural predictivity when using shuffled splits purely because it represents nearby time points similarly~~when using shuffled splits~~. To demonstrate this, we constructed a trivial model which operates only on the principle that stimuli nearby in time should be assigned similar representations. We term this model the Orthogonal Autocorrelated Sequences Model (*OASM*) because the activations of *OASM* are orthogonal for distinct passages/sentences/stories, and autocorrelated for stimuli within a passage/sentence/story~~(4.4) .~~ . Concretely, let n be the number of stimuli (rows) in the dataset. *OASM* is obtained by taking the $n \times n$ identity matrix and applying a Gaussian filter to on-diagonal blocks corresponding to temporally contiguous segments (passages in *Pereira2018*, sentences in *Fedorenko2016*, stories in *Blank2014*). This yields a smooth sequence within each segment and complete orthogonality between segments. By construction, therefore, *OASM* exhibits zero neural predictivity under contiguous train-test splits.

3. l. 93 "activations from GPT2XL using the last token of the stimuli" --> I might be blanking on the details from Schrimpf et al (2020), but how does this "last-token" method work for Fedorenko2016 (ECoG) dataset? In 4.1. you state that the ECoG dataset consisted of a 416 x 97 (words by electrodes) matrix. How do you model that dataset with a single (last/sum/mean) embedding per sentence? Or were the neural data averaged per sentence? Also, it could be useful to briefly remind readers with a short sentence in the main text (as you do in supplementary materials) why it is necessary to keep one embedding or average (due to fMRI temporal resolution etc.). Otherwise it isn't immediately clear from the text.

For Fedorenko2016, neural samples are provided per each word, and so we generate an embedding per word. For multi-token words, we either take the last token, sum across tokens, or take the mean across tokens for last/sum/mean embeddings respectively. For single-token words (which are the majority), the choice of activation extraction method has no effect. We now note more explicitly that the activation extraction is therefore not as critical of a decision not only for *Fedorenko2016* but for electrophysiology datasets in general, where word-level neural samples are the norm.

Changes to Discussion

For example, Jain et al. [2020] (Figure 3b). The choice of activation extraction method is less relevant for electrophysiological datasets due to the high temporal resolution. In the case of *Fedorenko2016*, the activation extraction method only differs in effect for words that are split into multiple tokens, and has a negligible impact on neural predictivity (Figure 3b). Based [illegible]

4. l. 127 "we use a one-sided paired t-test across square error values". What are the square error values referred to here? Sigmas? R^2?

Thank you for this comment. Squared error values are generated by taking the difference from the predicted brain responses and the actual brain responses, and then squaring this difference. For instance, squared error values are computed in *Pereira2018* by taking the regression predictions for each sample for a given voxel, subtracting them from the actual voxel activity, and then squaring this difference. We have added additional text in the results section to clarify what squared error values are, and include this text below. Importantly, squared error values are not independent, which violates one of the assumptions of the t-test. We elected to still use the t-test because the non-independence biases the statistical test towards reporting that LLMs explain meaningful variance over simple features. However, to account for this we also updated the manuscript to include results with a blocked t-test, where squared error values are averaged within each passage/sentence/block of consecutive TRs for Pereira2018, Fedorenko2016, and Blank2014 respectively before applying the t-test. All our statistical results using the blocked t-test are consistent with the original t-test.

Changes to Results

Third, we quantified the fraction of voxels in which *GPT2XL* explained significant additional variance beyond what OASM explained (i.e. where *OASM+GPT2XL* achieved significantly lower mean squared error than *OASM*. We assessed statistical significance using paired *t*-tests on squared error values across recording time points (the sample dimension). For each voxel/electrode/fROI, squared error values were computed by subtracting the predicted brain response from the actual response at each test sample and squaring the result. Test predictions were obtained from the cross-validation folds in which

each sample (sentence, word or TR) appeared in the held-out set, and the predictions from all folds were concatenated to yield one squared error value per sample. For *Pereira2018*, this gave $N = 384$ samples in EXP2 and $N = 243$ samples in EXP3, which we concatenated for participants present in both experiments (total $N = 627$ samples). For *Fedorenko2016*, the sample size was $N = 416$, and for *Blank2014* the sample size was $N = 1317$. We then applied a one-sided paired t-test across square error values to determine the percentage of voxels*t*-test for each voxel/electrodeselectrode/fROIs where fROI to determine whether squared error values were significantly lower when using *OASM+GPT2XL* explained significantly more neural variance than compared to *OASM* alone. We applied FDRcorrection alone. Importantly, these squared error values are not fully independent across samples, as adjacent samples within a passage, sentence, or consecutive TRs are temporally autocorrelated. This violates the independence assumption of the *t*-test in a direction that biases toward finding significant differences; thus, any significant effects should be interpreted conservatively. To mitigate this concern, we also implemented a blocked paired *t*-test, in which squared error values were averaged within autocorrelated blocks—passages in *Pereira2018*, sentences in *Fedorenko2016*, and non-overlapping consecutive TRs in *Blank2014*—and the *t*-test was applied to these block-averaged values. This blocked procedure yielded fewer significant voxels, reflecting its greater stringency. We report both sets of results, with false discovery rate (FDR) correction applied separately for each participant separately for this analysis [Benjamini and Hochberg, 1995](4.15)[Benjamini and Hochberg, 1995].

5. Fig 2b: If each dot in these scatterplots is a unit of analysis (voxel/electrode/fROI) for all participants -- how does one read the color-coded Pereira2018 figure? The color indicates #voxels but each dot is a voxel? Please clarify.

The color-coded figure for Pereira2018 is a 2D histogram, since there are too many voxels to plot each unit individually for this dataset. You are correct that the color represents the number of voxels,

however each dot is not a voxel but rather a histogram bin for the Pereira2018 figure. For the other datasets, we show a scatter plot since there are far fewer units to plot (97 in Fedorenko2016 and 60 in Blank2014). We have included additional text in the paper to clarify this, which we include below:

Changes to Results

Figure 2: **a)** Glass brain plots showing Pearson *r* values across the left hemispheric language network in *Pereira2018*. Plots are generated by averaging Pearson *r* values within the language network across all participants. Glass brain plots for individual participants are displayed in Extended Data Figure 6a. **b-d)** Top row is *Pereira2018*, middle row is *Fedorenko2016*, and bottom row is *Blank2014*. **b)** ~~2D histogram or scatter-plot comparing~~ Comparison of Pearson *r* values for *OASM* and *GPT2XL* at the voxel/electrode/fROI level. Bottom right corner shows the correlation in neural predictivity between the two models, with * indicating $p < 0.05$. A 2D histogram is used for *Pereira2018*, rather than a scatter-plot, due to the high number of voxels. **c)** Left-side shows the neural predictivity of *OASM*, *OASM+GPT2XL*, and *GPT2XL* in R^2. Lines connect dots from

6. l. 245. "LLM-based model of contextual syntactic representations" --> I really appreciate succinct reporting in the main text over all. I would just consider adding here a more descriptive sentence for "contextual syntactic representations". For example, "contextual syntactic representations obtained by averaging transformer embeddings extracted from sentences with the same part-of-speech and dependency tags, but different content words" or similar. Syntax means many things to different scholars and it is useful to briefly highlight your choice in the main text. If at all possible, I would also recommend showing (in supplementary materials) as small table with a sample of these generated sentences (and possibly their loss scores). Such that readers can get an impression of what is being averaged over.

We have added additional text to expand on how the contextual syntactic representations were generated in the Results, which we include below:

Changes to Results

found that $\Omega_{\text{GloVe}}(PWR)$ was around 55% and that $PWR+GloVe$ explained significant neural variance over PWR in 7.6% of voxels (Extended Data Figure 3), indicating that *GloVe* indeed explains neural variance over simple confounds. By contrast, an ~~LLM-based model of contextual syntactic representations~~LLM-derived model of syntactic representations, (*SYNTAX*), which was constructed by averaging LLM representations from sentences with the same part-of-speech and dependency tags, but different content words (4.7) explained much less neural variance over these simple confounds: $\Omega_{\text{SYNTAX}}(PWR)$ was over 80%, and the $PWR+SYNTAX$ model only explains significant neural variance over PWR in 0.00066% of voxels. [illegible]

We have also included a Supplementary Table showing a sample of the generated sentences which we show below:

Changes to Supplementary Section (Supplementary Table 3)

Original Sentences	Syntactically Equivalent Sentences
Beekeeping encourages the conservation of local habitats.	Gold refers the virus of public nests.<br>Gastrulation influences the protein of social cancers.<br>Steel helps the irritation of physical pesticides.
It is in every beekeeper's interest to conserve local plants that produce pollen.	It is in every person's art to categorize common surfaces that extend ability.<br>It is in every civilization's growth to cause pediatric opinions that stimulate nature.<br>It is in every victim's ground to abandon hot crystals that tap caffeine.
As a passive form of agriculture, it does not require that native vegetation be cleared to make way for crops.	As an ordered encephalomyelitis of impact, it does not collect that small loss be honored to keep language for crashes.<br>As a neat unemployment of culture, it does not cause that specific iron be performed to do plant for reptiles.<br>As a seminal appetite of acidity, it does not catch that social lacquer be passed to make mortality for fibroids.
Beekeepers also discourage the use of pesticides on crops, because they could kill the honeybees.	People rather avoid the damage of habitats on ecosystems, because they could grow the compounds.<br>Cockroaches also use the access of pipes on pores, because they could spend the deposits.<br>Species now save the water of grasses on groups, because they could play the stingers.

Table 3: Original sentences from a passage in *Pereira2018*, as well as 3 sample syntactically equivalent sentences for each.

7. Modality effect. It seems to me (Figs 1.b and 2.d) that OASM accounts for more neural predictivity in the listening dataset (Blank2014) compared to the two reading datasets. I am curious if this in your opinion warrants a smal l comment in discussion: Is it the case that the temporal autocorrelation is a a more pronounced problem in auditory stimulation than reading? Or is it just that LLMs happen to explain less variance in the auditory dataset to begin with?

There are many differences between the datasets beyond stimulus modality. The key reason why we believe that the Blank2014 dataset is so well predicted by OASM is simply that adjacent time points are separated only by 2 seconds in this dataset, whereas sentences are separated by 8 seconds in Pereira2018. Although time points are separated by either 450 or 700 ms (depending on the participant) in the Fedorenko2016 dataset, the fact that this dataset is ECoG and hence does not depend on a temporally smeared BOLD signal reduces the extent to which nearby time points are inherently correlated. Hence, although stimulus modality might play some role (e.g. maybe neural activity is more temporally correlated at adjacent time points when listening than when reading), any such effect is confounded with differences in temporal resolution and recording modality.

8. l. 249. "we find that a combination of positional signals, word rate, and static word embeddings predicts almost as much — or more — neural variance than GPT2XL". --> I wonder if you can speculate -- again, briefly -- on what your results say on transformer architecture. Some (most?) transformer-type models, like GPT2, have both "static embedding" and "positional embeddings" (layer 0 in your reporting) that get mixed into the hidden layer representations ("intermediate layers" in your terminology). But layer 0 don't seem to be competitive in predicting neural data based on eye-balling Fig 2.e. But based on the conclusions in this report (which I'm sympathetic to) and the logic behind OASM, wouldn't we expect the initial transformer layers to be the most competitive?

Thank you for these comments. We agree that the pattern of across-layer neural predictivity warrants more discussion given our findings with OASM and the simple models. We offer our speculations for the across-layer predictivity patterns, and specifically the performance of the initial embedding layer, for both the shuffled and contiguous split cases below.

We would like to clarify that when using shuffled splits, the primary determinant of neural predictivity is whether a model represents nearby timepoints similarly (e.g., autocorrelation within passages for Pereira2018) and distant timepoints dissimilarly (e.g., orthogonality between passages for Pereira2018). While positional embeddings can fulfill the autocorrelation-within-passage criteria, they do not satisfy the orthogonality-between-passages criteria. In fact, positional embeddings alone would be highly similar for the same position between different passages. It is possible that after the initial embedding layer, the nonlinear combination of position and word embeddings implemented by transformer layers produces representations that are more orthogonal between passages/sentences/stories. This may explain why the neural predictivity of the initial embedding layer is lower than the subsequent layers across all three neural datasets when using shuffled splits. We add additional text to the results clarifying the distinction between positional signals and OASM.

<u>Changes to Results</u>

processing of linguistic content; other factors might explain this gap. In particular, we introduce a simple baseline, the Position and Word Rate (*PWR*) model, which encodes only positional signals and word rate information (4.5). Positional signals encode stimuli by their position within a passage, sentence, or story, and are distinct from *OASM* in that stimuli across passages, sentences, or stories are not represented orthogonally. While *PWR* matches static models in the *Pereira2018*

When using contiguous splits, Figure 2e shows more competitive performance between the initial embedding layer and subsequent layers. The one exception is when using last token pooling with *Pereira2018,* which is likely because the last token is always a period in this dataset, and so last token pooling only encodes positional information whereas sum and mean pooling also incorporate some degree of linguistic information. In *Pereira2018*, we show that while *PWR+GloVe* is competitive with the intermediate layers of *GPT2XL*, it performs slightly worse (Figure 3a). This is consistent with the observation that the initial embedding layer, which also encodes only positional signals, word rate information, and non-contextual word embeddings, performs slightly worse than the intermediate layers. In *Fedorenko2016*, we observed that *PWR* is almost on par with the best intermediate layer of *GPT2XL*. It is indeed interesting that our *PWR* model achieves slightly higher neural predictivity than the initial embedding layer of *GPT2XL*, which may be because *PWR* is less prone to overfitting due to its small size or because *PWR* represents positional signals and word rate in a way that is easier to linearly map onto the brain. Finally, in *Blank2014 PWR* outperformed the initial embedding layers of *GPT2XL* by a large margin. While the initial embedding layers of *GPT2XL* also contain positional embeddings, we constructed *PWR* for *Blank2014* to primarily encode the initial ramping signal in brain responses based on the observation from Antonello et al., 2023 that fMRI responses at the beginning of stories (~100 s) show a ramping positional artifact. The positional embeddings of *GPT2XL* were not constructed to explicitly account for this positional artifact, which may account for the superior performance of *PWR.*

9. Compute resources. Would it be possible to add a section in Supplementary materials (or similar) on approximate computational resources that you used to run an experiment. I think even just a ballpark indication for the least and the most intense jobs would be helpful for anyone attempting to reuse the code (e.g. To run experiment on dataset 1 with 1 model, we requested between XY amounts of RAM on a ZY cluster and GPU?)

We have added a computational resources section, which we include below:

Changes to Methods

4.16 Computational resources

Figures 1, 2, 5, and 6 were generated using an Ubuntu server with three Nvidia GeForce RTX 3090 GPUs and an AMD Ryzen Threadripper 3960X 24-Core CPU with 125 GiB of memory. Only one GPU was used for any given analysis. Figures 3 and 4 were generated using a single Nvidia GeForce RTX 4090 GPU and an AMD 7950X 16-Core CPU.

Minor

- l.86 "we constructed a trivial model" --> In the context of regression, the term model is reserved for regression coefficients -- OASM is a predictor/design matrix, right? (I understand the term model is loaded and and used across the board, but would be helpful for clarity).

Yes, OASM is a predictor matrix. We updated our manuscript to include a sentence in the beginning of our results clarifying our use of the term "model". We hope that while the use of the term "model" may be somewhat non-standard in the context of regression, this clarification resolves ambiguities.

Changes to Results

Throughout this study, the term model is used to refer to the features used to predict neural activity (i.e. the X matrix in the regression). We evaluated the neural predictivity of several models on three neural datasets: (1) *Pereira2018* (fMRI - passages)

- l. 67 "each outer fold": Terminology (inner-outer folds) not introduced at this point in Introduction. Perhaps add a generic sentence layout out the logic of cross-validation (e.g. "In cross-validation, the dataset is split into N folds, ... on each fold...") to make it clearer.

We have added additional text in the results to make our explanation of the inner-outer folds more clear.

Changes to Results

To assess a model's neural predictivity, we used nested cross-validation (4.10). In ~~each outer fold,~~ nested cross-validation, an outer loop partitions the data into an outer train set and test set, and an inner loop further partitions the outer train set into an inner train set and validation set. The validation set is used to select ridge and banded ridge regression hyperparameters, and the ~~subset of brain responses and model activations used for fitting the regression is termed the train set , and the held-out subset of brain responses and model activations used to evaluate the~~ regression is ~~termed the~~ then fit using these hyperparameters on the outer train set and evaluated on the test set. For models with multiple layers or hyperparameter settings, we focus our analyses on the layer/hyperparameter setting which achieves the highest neural predictivity on the test set (averaged across outer folds). (4.11). Unless otherwise noted, all our results are generated by averaging values within voxels/electrodes/fROIs within each participant, and then reporting the mean of the participant averages.

- l.177 "in the Blank2014 dataset" --> Add figure reference here (Fig 3.a)

We updated the manuscript to include this change.

- Stijn Hawinkel et al. reference lacking year.

We updated the manuscript to include this change.

- l. 270. "used in the most impactful study on LLMs" --> unless you provide a quantification, I would say "in one of the most impactful studies"

We updated the manuscript to include this change.

- Fig 2c: Is there a need to plot yellow and purple bars 3 times within a panel? I assume you've considered this, but wouldn't it be fine to just plot five bars (one for each model combination) since yellow and purple are the same currently?

While the yellow bars are the same within each panel, the purple bars denote the GPT2XL banded onto OASM for each respective activation extraction method. We present the yellow bars and corresponding per-participant results adjacent to each purple bar to make it easier to see how *GPT2XL* adds to *OASM* for each feature extraction method. We apologize for the confusion and added text to denote this.

Changes to Results

voxels. **c)** Left-side shows the neural predictivity of *OASM*, *OASM+GPT2XL*, and *GPT2XL* in R^2. Lines connect dots from the same participant. These neural predictivity values are used to compute the percentage of *GPT2XL* neural predictivity that is accounted for by *OASM*, or $\Omega_{GPT2XL}(OASM)$, displayed on the right side. Purple bars show *GPT2XL* banded onto *OASM* for each respective activation extraction method. **d)** Percentage of voxels/electrodes/fROIs where *OASM+GPT2XL*

- l. 343. "On the other hand," --> Missing citation reference in bibliography (see "?" symbol)

We updated the manuscript to include this change.

- l. 480 "scipy gaussian_filter1d function" --> Do make sure to cite the main software packages you used that provided critical functionality (e.g. Scipy, Numpy, transformers, pytorch). Most provide citation information on their websites (e.g. https://numpy.org/citing-numpy/).

We have included citations for all the mentioned topics.

- l. 539 "L2 penalty" --> Missing citation reference in bibliography (see "?" symbol)

We updated the manuscript to include this change.

Reviewer #3 (Remarks on code availability):

I thank the authors for submitting the code and code checklist! I did not thoroughly review the code base nor have I attempted to reproduce any experiments. I can confirm that a cursory documentation is available on the repository on how to use the code. The dependencies are provided as well and the code for reproducing the figures as well. Given my quick impression, it's a well-structured research code in most parts and I was able to quickly get an impression what a specific piece of code was trying to achieve.

I suspect (based on quick impression) that actually reproducing and reusing code might not be super straightforward. For example, the user is instructed to use `call_banded_reg.py`, but it comes with 10+ input arguments. These arguments have defaults and help entries, which is great, but it would be nicer to just provide an small instructive example of how this function can be called (For example, "To

run a GPT2 model on Fedorenko dataset, you can call it like this"). The organization is lacking in some parts. For example, I found lingering (?) `breakpoint()` statements in the code, the root of the repository contains `figure4_updated.py` which seems it belongs elsewhere, notebook files are stored along with python modules etc. I believe someone with extensive experience of python would be able find their way, however.

Being aware of the realities of code development and sharing in research, it seems in an acceptable shape for a research project code and will provide value to the researchers interested in this work.

We will make sure to update the code repository so that it is easier to use, and greatly appreciate the feedback.

Do make sure to include the license file in the repository as well.

We have included a license file in the updated repository.

Reviewer #4

Hadidi, Feghhi and colleagues show that certain results from a particular thread of papers fall victim to a methodological misstep: using cross-validation with shuffled train and test splits. For many types of data (e.g., fMRI data, natural language, or basically any time series data), using shuffled train and test splits means that samples in the test sets will be correlated with samples in the training sets due to autocorrelation. They show that two core results from a landmark paper by Schrimpf et al., 2021, using shuffled splits, can be captured by a simple “orthogonal autocorrelated sequences” model (OASM). These two results, (1) that unidirectional / autoregressive models (like GPT) outperform bidirectional / masked models (like BERT) and (2) that brain predictions correlate with next-word prediction, both effectively disappear in the Schrimpf et al., 2021, datasets when using properly contiguous cross-validation splits instead of shuffled splits. They also show that, under certain conditions, a simple “position and word rate” (PWR) model provides surprisingly high correlations on par with the language models.

This is a very timely paper, and likely to be somewhat controversial—although I think this paper is unequivocally correct in pointing out certain methodological missteps that undermine core results in prior work. I have two high level concerns: (1) that some readers will overgeneralize these findings and “throw the baby out with the bathwater”, and (2) that some of these “gotchas” are also due in part to relatively odd structure of the original datasets (as well as certain analytic choices that remain unexplored here). This creates a certain tension between accurately reproducing (suboptimal) analytic choices applied to particular datasets used by Schrimpf et al., 2021, and making more general recommendations for the field. I unpack these comments below, followed by a number of more specific methodological questions and clarifications. Overall, I think this is a useful paper, and correctives of this kind are part of a healthy science—but, for a paper like this, the authors need to be extremely judicious and precise in their application of the corrective to avoid causing more harm than good.

Major comments:

1a. This is my highest-priority comment: I think it's extremely important that the authors be very precise about which papers—and which associated results—are known to be affected by certain methodological missteps, and explicitly which studies (and results) are not. There are already plenty of people out there who would love ammunition to argue that (1) encoding models are generally useless for neuroscience (not true at all) and (2) that neural network models for natural language (e.g., LLMs) are not useful for modeling the human neural machinery for natural language (also not true). There is a real risk that the important correctives this paper highlights will be overinterpreted as an indictment of the entire research program—which includes many labs and papers that have in fact been very careful and have managed to avoid these methodological missteps. (This kind of pattern—where a legitimate corrective is introduced, but then overinterpreted—occurs repeatedly in the fMRI literature; for example "voodoo correlations", "cluster failure", etc; Vul et al., 2009; Eklund et al., 2016.) I think the authors are already making a good-faith effort to do this, but the more judicious and explicit the better in this respect.

Thank you for raising these points. We have made several modifications to the manuscript to ensure that the message is delivered more precisely, and avoids making broad, overarching claims about the entire field. These modifications are detailed in the <u>Changes to Framing</u> section.

1b. Concretely, I would first suggest that the authors more explicitly and comprehensively enumerate which papers are subject to particular criticisms. For example, as a spot-check for shuffled cross-validation, I confirmed that Caucheteux et al., 2021, 2022, 2023, Caucheteux & King, 2022, all explicitly state in writing that they're using temporally contiguous splits for cross-validation. Similarly, work from Huth and colleagues typically uses a completely held-out test stimulus; e.g., Antonello et al., 2021, 2023; Antonello & Huth, 2024. Toneva et al., 2022, cross-validate to separate scanner runs. All work from Hasson and colleagues (that I have been personally involved with) has used contiguous cross-validation folds; Goldstein et al., 2022, 2025; Hong, Wang et al., 2024; Kumar, Sumers et al., 2024; Zada et al., 2024; and some studies, like Goldstein et al., 2024, put more stringent tests of generalization at the core of the work. This is of course a biased selection based on my own personal familiarity, but a spot-check of this kind suggests to me that a majority of published work does *not* directly fall victim to the methodological issue of shuffled cross-validation splits. I know it's quite difficult to verify and list out all non-offenders in this way, but I think it's incumbent on the authors to ensure that specific criticisms, e.g., about missteps in cross-validation, do not bleed over into studies that put in the effort to do things right.

We now provide such an enumeration of 35 studies in the field in Supplementary Table 1. An excerpt of this Supplementary Table is provided in the <u>Changes to Framing</u> section. We hope that this table provides a better holistic view of the field and highlights the fact that many studies use contiguous splits and properly account for confounds.

We would also like to note that, in the process of reviewing the literature, we noticed the following line describing train-test splits for encoding analyses in Goldstein et al. 2022: "The neural data were randomly split into a training set (that is, 90% of the words) for model training and a testing set (that is, 10% of the words) for model validation." Hence, as far as we can tell, it seems that Goldstein et al. 2022 also used shuffled splits. Though we make note of this in our literature review table, we emphasize important caveats to this in our Discussion. Specifically, although Goldstein et al. 2022 reported using shuffled splits, most of their analyses occur in the single case where we observe a minimal effect of shuffling: word-embedding models on electrophysiological data. We address this in a paragraph where we also address Mischler et al. 2024, in reference to your Comment 11. This paragraph is provided in the <u>Changes to Framing Section,</u> specifically under <u>Changes to Discussion (A)</u>.

1c. I would also suggest that authors be very clear that failing to replicate certain results from Schrimpf et al., 2021, does not necessarily mean those results won't hold up elsewhere. For example, the correlation between encoding performance and perplexity in Figure 4 has been found by Caucheteux & King, 2022, in both fMRI and MEG, using much larger samples (fMRI N = 100, MEG N = 95). Antonello & Huth, 2024, also replicate this result in a small-N dataset where each subject listened to *5 hours* of naturalistic story stimuli (despite using this result to illustrate a different point). On the other hand, the samples sizes here are quite small (N = 10, N = 5, N = 5) and the stimuli are generally non-naturalistic and more simplistic / impoverished (at least in Pereira2018 and Fedorenko2016; which may undercut the power of LLMs). This suggests that certain results under consideration here are not invalid generally, just that they cannot be replicated using the *specific* datasets from Schrimpf et al (and here) and the *specific* methodology used by Schrimpf et al. There are many possible reasons for this that do entirely hinge on the methodological correctives discussed here; for example, these results may not replicate because the structure induced by the experimental design (e.g., stimulus on/off blocks, matched/isolated sentences, etc) may overpower the structure of the actual language stimuli; or the language stimuli may not be sufficiently diverse or engaging or meaningful to subjects.

We agree that the failure to replicate the next word prediction result does not invalidate its appearance elsewhere and that the oddball nature of Schrimpf et al. 2021's datasets could have influenced these results. We now make this clearer in our Discussion, which we provide in the Changes to Framing Section, specifically under Changes to Discussion (C).

1d. By "specific methodology", the first thing that comes to mind is that Schrimpf et al., 2021, use a very restrictive set of voxels based on individual-specific functional localizer tasks. This analytic choice is imported directly into the current work and otherwise unexplored. These tasks simply contrast actual sentences versus nonword sentences, and will tend to focus on regions that have uniformly high activation for the actual sentences (greater than the nonword sentences). On the other hand, encoding performance in an analysis of this kind will be driven by reliable *variance* across words/sentences with different meanings (the model-based predictions are mapped onto up-and-down fluctuations in the signal across TRs / words / sentences; if the signal is uniformly high, it would result in poor encoding performance). Other research groups have compiled a large body of results indicating that different components of natural language comprehension extend beyond these areas (e.g., Huth et al., 2016; Nastase et al., 2021), and many voxels outside these regions exhibit strong encoding performance with LLMs (e.g., Caucheteux et al., 2022; Kumar, Sumers et al., 2024). I don't know if this is true, but it's certainly possible that this particular way of defining language regions yields a set of voxels that are unusually well modeled by OASM- or PWR-style models. My point here is that, as written, the current set of non-replication results are limited to this method for selecting voxels of interest. If the authors want to make broader claims, they would have to reproduce these null results across a wider set of voxels.

We agree with the reviewer that our claims are restricted to the language network. Although we believe that the use of contiguous splits, consideration of activation extraction methods, and accounting for low-level confounds is critical in other functional networks as well, it is entirely possible that for instance *PWR* will account for less of the neural variance that LLMs explain in other functional networks. We believe this analysis is outside the scope of our current study since the main point of our study is to reanalyze previous results, most specifically Schrimpf et al., 2021 and its follow-up studies, and these previous results almost exclusively focused on the language network. We have added an additional paragraph to our discussion to highlight the focus of our results on the language network as a limitation, which we include below:

Changes to Discussion

Fifth, it is also important to note that, following Schrimpf et al. [2021] and its follow-up studies, we restricted analyses to individually defined language network fROIs previously found using a sentences > nonwords localizer, to maximize comparability. This choice focuses on regions with robust mean responses to sentences, and these regions show reliable variation in response strength across sentences (Tuckute et al. [2024]; Fedorenko et al. [2025]), and such variation correlates with psycholinguistic variables (e.g., Shain et al. [2022]). However, voxels with lower mean, but richer time-varying signal, may fall outside these fROIs. Indeed, prior work suggests that language-relevant representations extend beyond these canonical functional regions [Huth et al., 2016, Nastase et al., 2021, Fedorenko et al., 2024], and LLM features can yield strong encoding outside these regions [e.g. Ayesh et al., Caucheteux and King [2022], Kumar et al. [2024]]. It is possible that simple baselines, specifically position and word rate, are particularly advantaged within our fROIs relative to other regions. Finally,

2. My second high-level comment (related to comment 1c): One of the sources of my confusion when reading this is that I hadn't realized quite how (let's say) "weird" the datasets used by Schrimpf and colleagues (2021) were. As someone who primarily uses longer-form, naturalistic stories/narratives as stimuli, I found myself wondering how many of these methodological and interpretational gotchas are partly symptoms of using these oddball datasets. For example, the authors say these results "depend somewhat unpredictably on the choice of dataset, models, and methods." This is patently true of many results, but all datasets are not created equal—examples where some of these results hold up are in much larger, more diverse datasets (see comment 1c). If the authors' goal is to show that this result does not hold up in the oddball Schrimpf et al., 2021, datasets alone, then that's fine, and they should be very explicit about that. If the authors want to claim that this result does not hold up *in general*, they would need to reproduce the null result on much larger, more diverse, more naturalistic datasets used by other groups; for example, LeBel et al., 2023, Nastase et al., 2021, Zada et al., 2025, or Li et al., 2021. At the very least, the authors should discuss the limitations of the datasets used by Schrimpf et al., 2021, versus larger, more naturalistic datasets.

We totally agree, and we make these points clear in our revised discussion. Specifically, in the Changes to Framing Section, Changes to Discussion (C), makes explicit reference to the oddball aspects of the datasets in Schrimpf et al. 2021, explicitly constrains our claims to these datasets, and notes that other results in the literature regarding the next-word prediction correlation lean positive, and our paper does not adjudicate on these cases. In the Changes to Framing Section, specifically under Changes to Discussion (D), we expand upon our first limitation which more generally emphasizes the unique nature of the three neural datasets we used relative to larger, more naturalistic datasets.

3. Related to the previous comment, I think the authors should unpack the structure of the datasets in a bit more detail, as this will impact the interpretation of their findings. For example, I stumbled when I got to line 58: "participants read short passages presented one sentence at a time, and a single fMRI volume (TR) was acquired after presentation of each sentence." That's strange. I understand that during a reading task, timing of fixations will be variable / idiosyncratic. Does this mean participants read the sentence, pressed the button to indicate they were finished, and following data were used for analysis? The scanner is presumably acquiring TRs continuously across an entire run, so I assume "a single fMRI volume (TR) was acquired" actually means that all *other* TRs acquired during sentence reading and the intervening gap were *discarded* for analysis. What is going into the context window when extracting embeddings for a dataset like this? In a paper like this, I don't think it's sufficient to simply redirect the reader to the original papers for further detail.

You are correct here, and we thank you for pointing out this error in our previous description of the preprocessing in the Pereira2018 dataset. We previously misinterpreted the preprocessing description in Schrimpf et al. 2021 (from which we use all of the same preprocessed datasets). Indeed, TRs were acquired continuously every 2 seconds across the entire run for Pereira2018. Critically, it was not the

case that only a single TR was used per sentence; rather, sentence-level responses were acquired using a mass-univariate GLM, in which a boxcar predictor was used for each sentence and then convolved with a canonical HRF; the resulting beta-weights from each sentence's regressor to each voxel were used as that sentence's response. We now explain the preprocessing details in greater depth, stating all of the details as presented in Schrimpf et al. 2021.

Changes to Methods

4.15 Preprocessing details

We use all preprocessed datasets as provided by Schrimpf et al. [2021]. We summarize their preprocessing pipelines below.

***Pereira2018* (fMRI).** Data were preprocessed in SPM5 (default settings unless noted): motion correction (realignment to the mean of the first functional run, 2nd-degree B-spline interpolation), normalization (estimated on the mean image with trilinear interpolation), resampling to 2 mm isotropic voxels, spatial smoothing with a 4 mm FWHM Gaussian kernel, and high-pass filtering with a 200-s cutoff. A standard mass-univariate GLM was fit in SPM5 to estimate the response to each sentence in each run using a boxcar regressor convolved with the canonical HRF; first-order temporal derivatives were included (but not used downstream) along with nuisance regressors for runs and offline motion parameters. Functional localization targeted the bilateral fronto-temporal language network using the sentences > nonwords localizer (Fedorenko et al., 2010) with group-constrained, participant-specific masks covering six regions per hemisphere (IFGorb, IFG, MFG, AntTemp, PostTemp, AngG); within each mask the top 10% most localizer-responsive voxels (highest t-values) were selected. Stimulus–response matrices were constructed by averaging the sentence-level responses across the three repetitions, yielding one value per sentence per language-responsive voxel.

***Fedorenko2016* (ECoG).** Signals (n=5 participants) were digitized at 1,200 Hz. Preprocessing followed Fedorenko et al. [2016] with noted deviations: (i) high-pass filter at 0.5 Hz; (ii) removal of reference, ground, and high-noise electrodes; (iii) common-average reference applied within amplifiers for electrodes below a preset line-noise threshold; (iv) notch filters removed 60 Hz line noise and harmonics. High-gamma activity was extracted via a Gaussian filter bank centered at 73, 79.5, 87.8, 96.9, 107, 118.1, 130.4, and 144 Hz (SDs 4.68–6.62 Hz); the analytic signal was obtained with the Hilbert transform, the eight band envelopes were averaged to form the high-gamma trace, z-scored per electrode across the session, and downsampled to 300 Hz (Schrimpf et al. [2021] did not additionally low-pass at 100 Hz, unlike in the original study Fedorenko et al. [2016]). Language-responsive electrodes were defined via the same sentences > nonwords contrast as the fMRI localizer ($p < .01$, per participant). For the core 52 sentences (8 words each), responses were averaged over each word's full presentation window, producing a 416 × 97 (words × electrodes) matrix across the five participants.

Blank2014 (fMRI). Five participants listened to 8 narrated stories from the Natural Stories Corpus (TR = 2 s; 1,317 TRs total). Per Blank et al. [2014], signals were filtered between 0.008 Hz and 0.09 Hz, thereby removing slow drifts and high frequency noise. Analyses were restricted to language-responsive voxels defined with the same sentences > nonwords localizer and group-constrained, participant-specific masks. For alignment, story audio was segmented into consecutive 2-s intervals matched to TRs (words straddling boundaries were assigned to the interval in which they ended). Given the HRF delay, each BOLD time point was assumed to reflect text that occurred 4 s (2 TRs) earlier. We also include a supplementary analysis using model representations from the previous 4 TRs concatenated together, as in Huth et al. [2016], in Extended Data Figure 8. For each participant, BOLD signals were averaged within each language fROI to improve SNR, yielding 12 fROIs per participant (6 per hemisphere; 60 total). Concatenating all story fragments produced a 1,317 × 60 (TRs × fROIs) matrix.

4. Following on the previous comment, I'm curious exactly how these datasets were preprocessed and whether the particularities of the preprocessing may impact the results observed here. For example, if linear drifts were not factored out of the fMRI time series, this would certainly contribute to the success of the OASM model. If the on/off structure of stimulus blocks was not accounted for in some way, this could both (a) hurt LLM encoding performance (due to large stimulus on/off signal fluctuations that are not related to the linguistic content), or (b) enhance the performance of the PWR model (e.g., if the signal ramps up at the beginning of a stimulus block, then tapers off over the course of the sentence). For example, in Zada et al., 2025, we explicitly include low-level confounds as well as task-level on/off block regressors to rule out these factors.

Thank you for flagging this. For all datasets, we used the preprocessed data provided by Schrimpf et al. [2021]. For Pereira2018 and Blank2014, this includes a high-pass filter to remove slow

scanner/physiological drifts (as described now in the "Preprocessing details" section discussed in response to Comment 3); thus linear or slow trends are strongly attenuated. With respect to task on/off structure, our analyses do not rely on alternating blocks that would create large stimulus-locked swings: in Pereira2018, all sentence-level responses correspond to when the stimulus was "ON". Blank2014 is continuous naturalistic listening without task/rest alternation; and in Fedorenko2016 (ECoG) we use word-locked neural windows. Because there is no block alternation coincident with the analysis windows, additional task-level on/off regressors are not applicable here. We agree with the general point exemplified by Zada et al., 2025 that low-level confounds and block regressors should be modeled when bona-fide block structure exists (as in the case of dyadic conversations), and we now state this explicitly in the Discussion (see <u>Changes to Framing</u>, specifically <u>Changes to Discussion (B)</u>).

5. It should be explicitly noted that, at least since Huth et al., 2016, many fMRI studies in this vein have included low-level confound regressors like word rate; e.g., Huth et al., 2016, used word rate, phoneme rate, and phonemes alongside their semantic model of interest; similarly for our work in Kumar, Sumers et al., 2024, where we include phonemes, phoneme rate, word rate, and a silence indicator in a separate nuisance band using banded ridge regression (linguistic features of interest will be correlated with these low-level confounds in most cases of natural language).

We agree. We now make this clearer in our discussion, where we include these studies as well as several others that account for low-level confounds. We include this paragraph in the <u>Changes to Framing Section</u>, specifically under <u>Changes to Discussion (B).</u> We also give a more comprehensive list of papers that have made efforts to account for confounds, and we describe the confounds accounted for in depth in the new literature review table in Supplementary Table 1.

6. At lines 555 and 608, you say "For R-squared, we clip results to be at least 0 at the voxel / electrode / fROI level to prevent noisy values from biasing the mean downwards." Is there any precedent for doing this fairly aggressive data imputation? In my experience, out-of-sample R-squared values in this kind of analysis can be *very* low, with many going strongly negative (i.e., providing poorer predictions than the mean on the test set). This can be a useful tool for diagnosing whether the modeling pipeline (cross-validation, normalization, hyperparameter selection, etc) are working properly. I'm curious about what proportion of voxels end up getting clipped in these analyses… For example, if you only have 243 voxels to begin with (Pereira 2018, Experiment 3) and half of them disappear due to negative R-squared values, that would give me major pause when interpreting the current results.

We apologize for the confusion. In the initial submission, we incorrectly state at the referenced line that we clip the results to be at least 0 at the voxel / electrode / fROI level for the R^2 values. This is a vestige of an earlier draft, and it is not true of the submitted work; we do not adjust the R^2 values of individual voxels / electrodes / fROIs before taking the mean within participants. However, we do clip the within-participant means to be at least 0 before averaging across participants to prevent individual noisy participants from biasing mean results downwards, as well as to prevent nonsensical Omega values.

<u>Changes to Methods</u>

For both metrics, we take the mean neural predictivity across voxels/electrodes/fROIs for each participant, ~~and compute the mean value across participants. We refer to R^2_{oos} as R^2 throughout the rest of the paper for brevity. For R^2, we clip results to~~ clip these within-participant mean values to be at least ~~0 at the voxel/electrode/fROI level~~ 0 to prevent noisy ~~values~~ users from biasing the mean downwards, and then compute the mean value across participants.

It is also important to clarify that, as further expanded upon in our response to Comment 8, we have 243 *samples*, not *voxels*, in Pereira2018 Experiment 3. The total number of language network voxels in Pereira2018 across the 10 unique participants in Experiments 2 and/or 3 is 13,553.

7. I was a bit confused about the statistical tests used for model comparison. For example, at line 126, you say: "Third, because Ω is defined at the participant level, we use a one-sided paired t-test across square error values to determine the percentage of voxels / electrodes / fROIs where OASM+GPT2XL explained significantly more neural variance than OASM alone. We applied FDR correction for each participant separately for this analysis." What are the samples going into this t-test if you're doing the test within each participant? (i.e., what are the replicates determining the degrees of freedom in the test?) If I'm reading the Methods correctly, starting at line 617, you're treating voxels (e.g., N = 384) within the language ROIs as samples? This is fairly nonstandard, and I'm not really sure what inferential interpretation it has. I'm also not sure how that relates to the term "percentage" in your description of the test. Alternatively, you might be supplying sentences or test folds into the statistical test? A different approach here to performing a within-subject statistical test would be to randomize the mapping between the time series and the embeddings somehow; e.g., phase randomization, random circular rotation of the time series, or block-wise permutation (as in Lebel et al., 2023). When you say "FDR correction for each participant separately", FDR correction across *what* multiplicity of tests exactly? Across different model comparisons? I don't necessarily think any of this is wrong; but please clarify these points in both the Results and the Methods.

We apologize for the confusion, and we provide a response for each comment individually below.

What are the samples going into this t-test if you're doing the test within each participant? (i.e., what are the replicates determining the degrees of freedom in the test?) If I'm reading the Methods correctly, starting at line 617, you're treating voxels (e.g., N = 384) within the language ROIs as samples?

We apologize for the confusion, the samples for the t-test are not the voxels. The samples for the t-test (denoted as N in the study) are the number of time points of recorded neural activity for each voxel/electrode/fROI. For voxels in participants in *Pereira2018* with only data from experiment 2, N equals to 384. For the voxels in participants in *Pereira2018* with only data from experiment 3, N equals to 243. For voxels in participants in *Pereira2018* with data from both experiments, N=627. For *Fedorenko2016*, N = 416, and for *Blank2014*, N=1317. We apologize for the confusion, and have updated our methods section to make this more clear.

0.05 is used to determine significance. The sample size for this t-test was $N = 384$ (where N refers to the number of samples) for voxels in *Pereira2018* with only data from *EXP2*, $N = 243$ for voxels in *Pereira2018* with only data in *EXP3*, and $N = 627$ for voxels with data in both experiments. For *Fedorenko2016*, the sample size was $N = 416$, and for *Blank2014* the sample size was $N = 1317$.

I'm also not sure how that relates to the term "percentage" in your description of the test.

The term "percentage" refers to the percentage of voxels/electrodes/fROIs where including the LLM to the baseline model (M + LLM*) explains significantly more neural variance than the baseline model (M) alone. We compute this percentage in the following manner:

1) Compute squared error values from both M+LLM* and M for each voxel/electrode/fROI. Squared error values are computed by subtracting the regression predictions from the neural activity for that given voxel/electrode/fROI, and then squaring this difference.
2) Apply a one-sided paired t-test between the squared error values from M+LLM* and M to evaluate whether the squared error values from M+LLM* are significantly smaller than M. A

significant result here indicates that for that given voxel/electrode/fROI, M+LLM* explains significant neural variance over M alone.

3) Apply FDR correction to the p-values obtained from Step 2 within each participant.
4) Compute the of percentage of voxels/electrodes/fROIs where including the LLM to the baseline model (M + LLM*) explains significantly more neural variance than the baseline model (M) for each participant after FDR correction. Percentages for each participant are visualized as dots, and the mean across participants is visualized as a bar in the respective figures.

Alternatively, you might be supplying sentences or test folds into the statistical test? A different approach here to performing a within-subject statistical test would be to randomize the mapping between the time series and the embeddings somehow; e.g., phase randomization, random circular rotation of the time series, or block-wise permutation (as in Lebel et al., 2023).

We updated our manuscript to perform a blocked paired t-test. We first describe the blocked paired t-test. Next, we explain why we used a blocked t-test over a blocked permutation test as recommended by the reviewer. Finally, we justify why we also included results with the standard (non-blocked) t-test throughout the manuscript.

The N for the paired t-test is the number of time points in the dataset. Given that much of the point of our paper is that such time points are correlated (i.e. not independent samples), this violates one of the assumptions of the t-test.

In order to address this concern, we reproduced all our main statistical results with *GPT2XL* and *GPT2XLU* using a blocked paired t-test. In order to perform a blocked paired t-test, we averaged squared error values within each passage (*Pereira2018)*, sentence (*Fedorenko2016)*, and within each group of consecutive 10 TRs (which corresponds to 20 seconds, *Blank2014)*. Averaging within each passage/sentence/non-overlapping TR window mitigates the non-independence issue. These results are displayed in Extended Data Figure 7, and referenced throughout the results section. In summary, our main claims are consistent when using either the blocked paired t-test or the original, "non-blocked" paired t-test.

Importantly, the sample size after averaging was still large enough to assume normality for each neural dataset, with the minimum sample size being 52. Since there were enough samples, we elected to use a blocked paired t-test rather than a blocked permutation test.

We would also like to note that the reason we show results with a "non-blocked" paired t-test throughout the study is based on the following logic. Our null hypothesis was that simple models (e.g., OASM, positional signals, word rate, GloVe) account for the neural variance that a LLM explains. The fact that brain responses are correlated in time inflates the probability of Type 1 error, in other words it inflates the probability that the statistical test indicates that an LLM explains significant neural variance over the simple models when it actually does not. Since a central message of our study is that simple models account for a large fraction of the neural variance LLMs explained on these neural datasets, the inflation of Type 1 error actually makes our claims more conservative. We discussed this in the "Statistical testing" section from the Online methods, and have added a note in the main results section to state this clearly.

<u>Extended Data Figure 7</u>

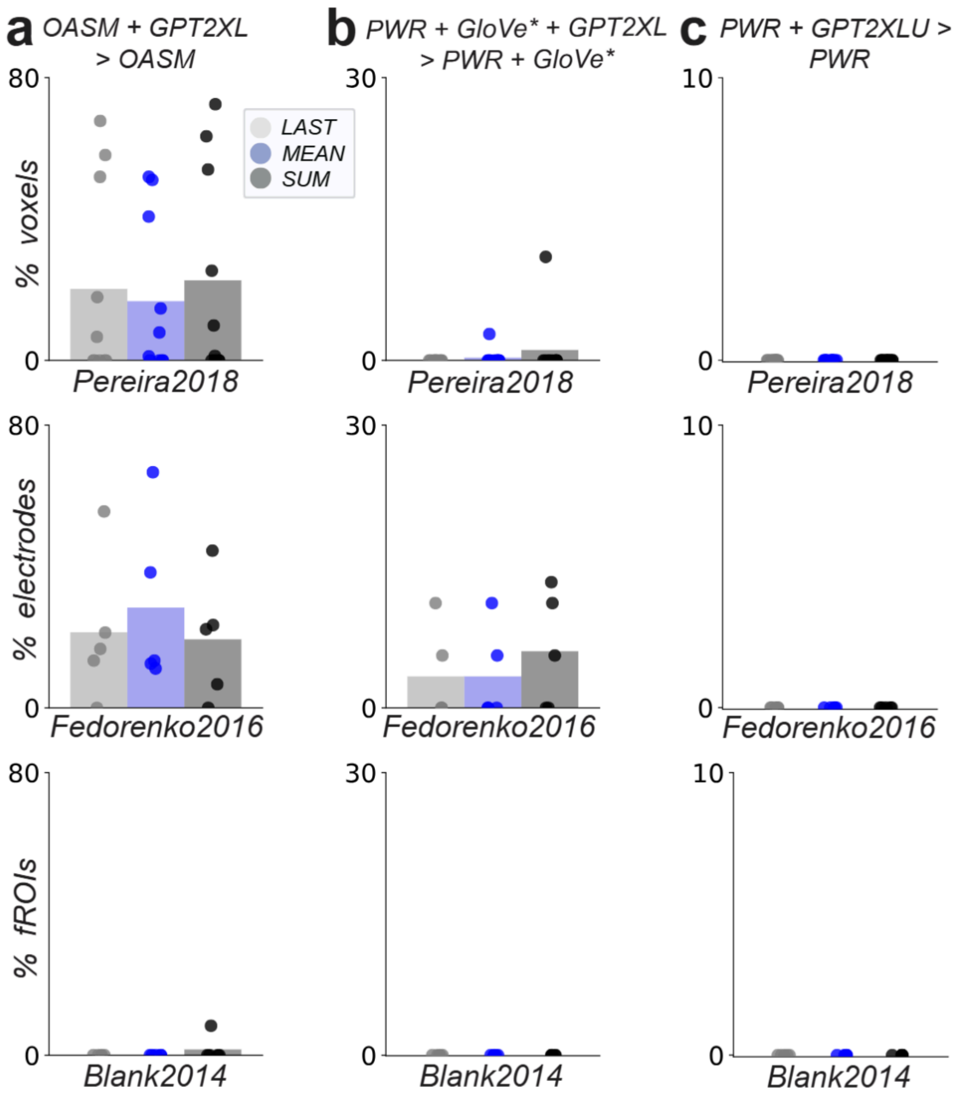

Figure 7: Reproduction of main results obtained from using the paired t-test, except t-test was applied after averaging squared error values within passages for *Pereira2018*, within sentences for *Fedorenko2016*, and for every 10 TRs within each story for *Blank2014*. Color legend indicates activation extraction method used for *GPT2XL/GPT2XLU*. **a)** Same as Figure 2**d** with modified statistical procedure. **b)** Same as Figure 5**e** with modified statistical procedure. * symbol above *GloVe* indicates that *GloVe* was only used for *Pereira2018*. **c)** Same as Figure 6**e** with modified statistical procedure.

Changes to Results

ance than *OASM* alone. Squared error values are computed by subtracting the predicted brain responses from the actual responses and squaring the result (4.14). Squared error values are not independent within a given passage, sentence, or consecutive TRs, which violates the assumptions of the t-test but biases results in favor of *GPT2XL* (see 4.14 for further explanation). As an additional check, we also report results with a blocked paired t-test, where the t-test was applied after averaging squared error values within each passage (*Pereira2018*), sentence (*Fedorenko2016*), or non-overlapping consecutive

... continued on next page in the manuscript

TRs (*Blank2014*) to mitigate non-independence (4.14). We applied FDR correction for each participant separately ~~for this analysis [Benjamini and Hochberg, 1995](4.14)~~[Benjamini and Hochberg, 1995].

Changes to Methods

To ensure findings using the paired t-test were robust, we replicated the analyses relating to *GPT2XL* and *GPT2XLU* using a blocked paired t-test. To perform the blocked paired t-test, we averaged squared error values within each passage for *Pereira2018*, each sentence for *Fedorenko2016*, and within non-overlapping 10 TR (20 sec) segments *Blank2014* (Extended Data Figure 7). Averaging mitigates non-independence since squared error values are most correlated within a given passage, sentence, or consecutive TRs. Sample sizes after averaging were still sufficiently large for the normality assumption: $N = 96$ for voxels in *Pereira2018* with only data from *EXP2*, $N = 72$ for voxels in *Pereira2018* with only data in *EXP3*, $N = 168$ for voxels with data in both experiments, $N = 52$ for *Fedorenko2016*, and $N = 138$ for *Blank2014*. Given that normality assumptions were met, we elected to use the a blocked t-test rather than a blocked permutation test.

When you say "FDR correction for each participant separately", FDR correction across *what* multiplicity of tests exactly? Across different model comparisons? I don't necessarily think any of this is wrong; but please clarify these points in both the Results and the Methods.

We applied FDR correction to the p-values obtained from the paired t-test across voxels/electrodes/fROIs for each participant.

8. At line 92, you indicate that Schrimpf and colleagues (2021) used ordinary least squares (OLS) to map GPT-2 XL embeddings onto the brain activity. Is this correct? I don't understand how this worked. Aren't the GPT-2 XL embeddings 1,600 features wide, with many fewer samples (TRs) in the fMRI data? (i.e., 384 and 243 samples in Pereira2018, 416 samples in Fedorenko2016, 1317 samples in Blank2014…) Wouldn't it be impossible to solve such a regression equation with OLS? Was Schrimpf doing PCA on the embeddings prior to regression? Or some pseudo-inverse trick? My understanding is the authors somehow replicated the same analysis using OLS (lines 92 and 533). How? In any case, I agree that the authors should focus on ridge for the core results, as this has become the standard in the field.

Yes, Schrimpf et al. 2021 used OLS. As you've noted, the OLS objective is underdetermined when num_features > num_samples. Nonetheless, most linear regression implementations (e.g. sklearn) handle this case by returning the unique exact solution with minimum L2 norm of the weights. This solution can be thought of as what you'd get from a ridge regression as your L2 penalty coefficient approaches 0; with any non-zero value for the L2 penalty coefficient, the optimization has to compromise training-set accuracy with L2 minimization, leading to non-exact (though typically more generalizable) solutions. We now make this clear in our Methods section describing regression methods.

Changes to Methods.

4.8 Regression methods

We used three linear regression methods throughout our paper. For all regression methods, we predict brain responses for each voxel/electrode/fROI independently. To compare our findings with Schrimpf et al. [2021], we employed ordinary least squares (OLS), which we implemented ourselves in order to run it on GPU. ~~For all other analyses, we elected not to use OLS regression, because we are generally working with very~~ In our setting where the number of features often exceeds the number of samples, the OLS estimator is computed via the Moore–Penrose pseudoinverse and yields the unique minimum-ℓ_2-norm solution among all least-squares minimizers; equivalently, it is the limit of ridge regression as the L2 penalty $\lambda \to 0^+$. When X has full row rank, this solution can exactly interpolate the training data, but it is typically

high-variance and ill-conditioned ~~linear mappings due to high-dimensional feature spaces, relatively few samples compared to the number of features, and noisy targets~~in practice—hence we restricted OLS to replication of Schrimpf et al. [2021]. We [illegible]

9. At line 542, you say "We z-score all predictors across samples before training regressions." I'm not demanding the authors re-run their models, but this is not the best practice. Z-scoring the entire dataset prior to cross-validation will introduce leakage between training and test sets. For a paper like this that hinges on methodological rigor, ideally you should z-score within the training set of each cross-validation fold; e.g., use scikit-learn's StandardScaler in a Pipeline to .fit_transform() each training set, then .transform() the corresponding test set using the mean and standard deviation of the training set.

We apologized for the confusion. We in fact did follow the exact pipeline that you propose (i.e. z-scoring with the training set of each cross validation fold and using those same statistics to normalize samples in that fold's set set). This is now made clear in Section 4.8:

> feature spaces (see Supplementary for ~~ane~~ an expanded description of banded ridge regression). We fit all L2-regularized regression models with the *himalaya* package from Dupré la Tour et al. [2022]. ~~We~~ Within each outer fold (4.10), we z-score all predictors across samples ~~before training regressions, as is standard when using ridge regression in neural encoding studies~~in the training set before fitting regression models, and we use the same training set statistics to normalize predictors in the validation and test sets.

10. How are you accounting for hemodynamic delays in the fMRI data? Note that many examples of current work use a finite impulse-response model where they horizontally stack lagged duplicates of the model features (embeddings), via Huth et al., 2016. This is a more sensitive approach, as it allows the model to estimate a linear combination of lags for each voxel / electrode in each subject.

We accounted for hemodynamic delays in a similar manner as Schrimpf et al. 2021 We describe the approach used in our Online Methods, specifically in the Preprocessing Details section which is included in response to the comments above.

In Pereira2018, hemodynamic delays are handled upstream when estimating the neural responses: a voxelwise GLM is fit with one boxcar regressor per sentence convolved with the canonical HRF, plus its first-order temporal derivative to absorb small latency shifts. We then analyze the resulting per-sentence beta estimates (averaged across repetitions)—one value per sentence per voxel—rather than TR-wise time series. Because the HRF has already been modeled in generating these targets, the FIR "stacked-lags" approach (e.g., horizontally stacking lagged copies of embeddings) is not applicable here: it's designed for direct regression onto continuous BOLD time courses and would (i) double-model the HRF and (ii) be incompatible with our one-value-per-sentence targets.

For the Blank2014 dataset, we originally only used one lag, as in Schrimpf et al. 2021, using only the embeddings from 4-6 second window preceding the current TR. In Schrimpf et al. 2021, only the last embedding in this window was used (last token approach), whereas here we also tried using the mean as well as the sum of embeddings in that window in our original submission. However, we agree that the horizontally stacked lagged duplicates are likely a superior approach, so we have re-evaluated GPT2-XL on the Blank2014 dataset using these 4 concatenated lags, using the embeddings from 2-4, 4-6, 6-8, and 8-10 seconds back from the current TR. We provide these results in Extended Data Figure 8. We generally see minimal changes in performance, with the models still showing considerably lower Pearson r than the position and word rate (PWR) model.

Extended Data Figure 8

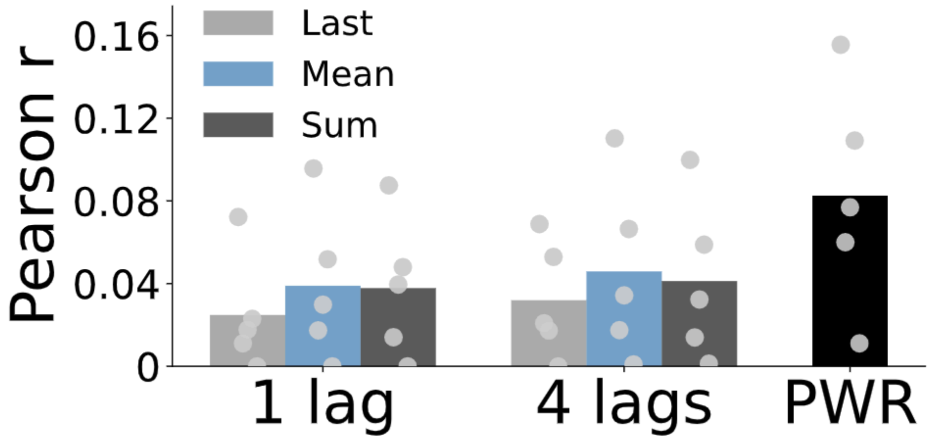

Figure 8: Results with *GPT2XL* on the *Blank2014* dataset when using 4 lags to account for the hemodynamic response. The leftmost group of bars labeled "1 lag" shows the encoding performance when using only the tokens from the 4-6 seconds before the present TR for encoding model estimation, as in Schrimpf et al. [2021] and our main results. The "4 lags" group of bars shows the encoding performance when instead concatenating tokens from 4 lags (2-4 s, 4-6 s, 6-8 s, and 8-10 s), as is commonly done in other language encoding studies with naturalistic datasets (e.g. Huth et al. [2016]). Lastly, we show the PWR model. Although using 4 lags allows *GPT2XL* to achieve slightly higher encoding performance than when using just 1 lag, it still falls far short of *PWR*. This indicates that the relatively low performance of LLMs relative to *PWR* on *Blank2014* persists even when using a more robust and standard method to account for hemodynamic delay. Individual dots correspond to the mean Pearson r for each participant. Each feature extraction method specifies how the tokens within each 2 second TR window are aggregated.

11. Do the authors have an intuition whether this problem of autocorrelation when using shuffled splits is worse for fMRI data? My intuition is that fMRI data is by nature much more temporally autocorrelated than ECoG data. For example, I agree that it's not ideal for Mischler et al., 2024, to use shuffled splits, but with the high temporal resolution and signal properties of ECoG, it might not be as egregious as for fMRI.

Yes, we agree with your intuition that the autocorrelation problem is worse for fMRI data than ECoG (or EEG/MEG) due to the temporally smeared nature of the BOLD signal. This is reflected in our results, where we find that although OASM dramatically outperforms GPT2 on the Blank2014 dataset (fMRI, TR every 2 seconds) and considerably outperforms GPT2XL on the Pereira2018 dataset (fMRI, sentences separated by 8 second delays), it roughly matches GPT2XL on the Fedorenko2016 dataset (ECoG, 450 or 750 ms between words depending on the participant). While this suggests that the problem is less severe for ECoG, it does not indicate that it is not a problem. OASM performs at the same level as LLMs, indicating that a trivial autocorrelation model offering no linguistic insights can match LLM performance.

Beyond just inflating the performance of models with OASM-like autocorrelation, we also find significant correlations between next-word prediction and neural predictivity for the Fedorenko2016 dataset only when shuffling. Considering that much of the results of Mischler et al. relate to correlations between neural predictivity and other task performance metrics, our results suggest that such correlations might not be robust when using contiguous splits. Hence, regardless of whether it is less egregious to use shuffled splits with ECoG than with fMRI, we strongly oppose the use of shuffled

splits in all contexts. In general, it not only inflates performance but also can produce spurious trends across models.

We make note of the fact that the effect of shuffled splits is less egregious for electrophysiological datasets than for fMRI:

Changes to Discussion

Although the majority of language encoding studies using these three neural datasets have employed shuffled splits, the use of shuffled splits is not pervasive in studies using other neural datasets. After conducting a literature review of 35 language encoding studies (Supplementary Table 1), we found that only 2 studies using other neural datasets shuffled: Mischler et al. [2024] and Goldstein et al. [2022]. Both these studies analyze electrophysiological signals, where the impact of shuffling is less pronounced (though still non-negligible). Moreover, Goldstein et al. [2022] focuses its analyses on *GloVe*, and we observed virtually no difference in the neural predictivity of static word embeddings models on *Fedorenko2016* when switching from shuffled to contiguous splits, likely due to the low temporal mixing rate of electrophysiological signals (relative to fMRI) and word embedding models (relative to LLMs). In sum, we emphasize that although the negative effects of shuffled splits might be less egregious in some circumstances, they are generally problematic and should be avoided.

12. The discussion of using shuffled splits (which I agree with the authors should basically never be used in the context of autocorrelated time series like fMRI data or natural language) could be developed into a broader point about generalization in model performance. Using cross-validation with temporally contiguous splits is certainly a good baseline recommendation, but it may be worth indicating to readers that other, even more stringent generalization tests can be theoretically interesting. For example, evaluating model generalization to entirely separate, fully held-out stimuli (e.g., Huth et al., 2016); evaluating model generalization across strictly non-overlapping sets of words (e.g., Goldstein et al., 2024); ensuring that context windows are reset at cross-validation folds (e.g., de Varda et al., 2025); and evaluating model generalization across subjects (e.g., Zada et al., 2024).

We agree that the discussion of shuffled splits can be expanded more broadly to generalization in model performance, and have added the following paragraph to our discussion:

Changes to Discussion

~~In addition to commenting on~~ Beyond just using contiguous splits, there are several ways analyses can be made more stringent. First, one can evaluate on entirely held-out stimuli (e.g., whole stories never seen in any train or validation fold), as in Huth et al. [2016]. Second, one can enforce strictly non-overlapping vocabularies between train and test (e.g., Goldstein et al. [2024]), which is especially stringent for word-level/ECoG settings and helps diagnose lexical memorization versus generalization. Third, resetting model context windows at cross-validation boundaries (e.g., de Varda et al. [2025]) prevents inadvertent context leakage when folds split within long stimuli. Finally, cross-subject generalization (e.g., Zada et al. [2024]) tests whether mappings learned on some individuals transfer to unseen individuals, though it introduces variability from inter-subject alignment. We view temporally contiguous cross-validation as a baseline, and these stricter regimes as complementary stress tests whose adoption should be matched to the stimulus structure and analysis goals. If one insists on using shuffled splits, ~~our findings~~ one can insert temporal buffer zones (e.g., ± 20 s) around train–test boundaries to reduce autocorrelation leakage; however, this approach discards substantial amounts of already limited data and is generally inferior to simply using temporally contiguous splits.

Minor comments:

Line 238: Can you summarize the paragraph starting at line 238 with a take-home message? Is the point here that this result is conceptually congruent with the paper by Kauf?

Yes, we have now added a take-home message here. The point is that linguistic features (especially syntactic features) are too confounded with simpler explanations like sentence position and word rate for Pereira2018 to be a reliable dataset for disentangling their contribution to LLM encoding performance.

Changes to Results

A previously explored scientific question on *Pereira2018* is whether the mapping between LLMs and brains on *Pereira2018* reflects lexical-semantics or syntactic information [Kauf et al., 2024]. Here, we found that combining *GloVe* with *PWR* explained more neural variance than either model alone, suggesting that lexical-semantic content (which we model with *GloVe*) ~~plays an important role in explaining LLM-to-brain mappings~~ accounts for some neural variance beyond simple confounds. To further examine this, we examined how much of the neural variance explained by *GloVe* could be accounted for by simple confounds. We found that $\Omega_{GloVe}(PWR)$ was around 55% and that *PWR*+*GloVe* explained significant neural variance over *PWR* only in 7.6% of voxels (Extended Data Figure 3), indicating that *GloVe* ~~indeed explains~~ explains some neural variance over simple confounds. By contrast, an ~~LLM-based model of contextual syntactic representations~~LLM-derived model of syntactic representations, (*SYNTAX*), which was constructed by averaging LLM representations from sentences with the same part-of-speech and dependency tags, but different content words (4.7) explained much less neural variance over these simple confounds: $\Omega_{SYNTAX}(PWR)$ was over 80%, and the *PWR*+*SYNTAX* model only explains significant neural variance over *PWR* in 0.00066% of voxels. Hence, our results indicate that the *Pereira2018* dataset is poorly suited for disentangling the linguistic features underlying LLM neural predictivity, as lexical-semantic and syntactic representations are strongly confounded with simpler explanations.

Line 343: “On the other hand, ?, Hong et al. [2024]”—missing citation (probably Antonello & Huth, 2023?)

We have fixed this issue in the updated draft.

Line 375: “contextual syntactic representation”—this doesn’t seem like very standard terminology to me (e.g., many people who care a lot about syntax work on “context-free grammars”; might be worth unpacking a bit more).

We agree that this language is somewhat confusing. It was taken directly from Caucheteux et al. 2021, where they distinguish “contextual” syntactic representations (which are attained by averaging the *intermediate* layers of GPT2-XL in response to many “syntactically equivalent sentences”) from “static” syntactic representations (which are attained by averaging the *static* layers of GPT2-XL in response to such sentences). However, because we do not use any static contextual embeddings here, we realize that the term “contextual” here is more confusing than its worth. We’ve revised the paper to now refer to these embeddings as *LLM-derived syntactic representations*. We include a detailed description of how we derived these embeddings in the Methods section titled “Syntax”, and now provide some intuition as to how they are formed when first mentioning them in Section 2.3

Changes to Methods

4.7 Syntax

Syntactic embeddings (*SYNTAX*) are derived by averaging together the embeddings of sentences that all share the same syntactic structure but differ in their content words. This procedure averages away differences in word/phrase/sentence-level meaning between the sentences, and keeps what is shared between them: syntactic structure (and abstract semantics associated with that structure). First, we create a word bank for each part-of-speech and dependency tag in the original sentences by passing 300,000 sentences from the Generics KB corpus ~~Bhakthavatsalam et al. [2020]~~ [Bhakthavatsalam et al., 2020] through the SpaCy transformer-based model [Honnibal and Montani, 2017]. Then, we run each sentence in *Pereira2018* through the SpaCy transformer-based model. We then generate new sentences, in which each content word from an original sentence is replaced with a content word of matching part-of-speech and dependency tag randomly sampled from the word bank. Each newly generated sentence is then once again passed through the SpaCy transformer-based model to get the token indices of the subtrees associated with each token; we ensure that, for each token index, the token indices of the subtree in the generated sentence match those in the original sentence. If not, we discard the generated sentence and try again. We continue until 170 new sentences with valid subtrees are generated for each original sentence. Finally, we take the cross-entropy loss of *GPT2XL* on each newly generated sentence and keep the 100 sentences with the smallest loss. To get a syntactic representation for each original sentence, we run each of these 100 new sentences through *GPT2XL* and average their representations. This method is highly similar to that of [Caucheteux et al., 2021], the main difference being that we keep function words from the original sentence. We keep function words for two reasons: (i) representations of function words have previously been used to generate representations of syntactic form for neural encoding ~~Kauf et al. [2024]~~[Kauf et al., 2024], and (ii) coherent sentences were generated much more often when function words were left intact. Altogether, our syntactic representations combine the constraints on part-of-speech and dependency structure, as in Caucheteux et al. [2021], and the preservation only of function words, as in Kauf et al. [2024]. We used sum pooling to extract activations for *SYNTAX* because it resulted in higher neural predictivity than mean pooling or using the last token.

Changes to Results

over simple confounds. By contrast, an ~~LLM-based model of contextual syntactic representations~~LLM-derived model of syntactic representations, (*SYNTAX*), which was constructed by averaging LLM representations from sentences with the same part-of-speech and dependency tags, but different content words (4.7) explained much less neural variance over these

In section 4.2 of the Methods, can you add a little more detail about GPT-2 XL? The main thing that occurred to me here is that I couldn't find the embedding dimensionality for XL… If I recall correctly, it's 1,600?

We have added text specifying the dimensionality of GPT2-XL.

Changes to Methods

4.2 Language models

For the majority of our analyses, we focus on *GPT2XL* [Radford et al., 2019]. We do so because *GPT2XL* was shown to be the best-performing model on *Pereira2018*, and performed on par or better than other models in *Fedorenko2016* and *Blank2014* [Schrimpf et al., 2021]. Additionally, it has been the main language model used in other neural encoding studies using these datasets [Hosseini et al., 2024a, Kauf et al., 2024]. *GPT2* is a unidirectional transformer model, meaning that it can only attend to current and past tokens (but not future tokens). It is trained on next-token prediction. The *XL* variant has $\sim 1.5B$ parameters and 48 layers, with an embedding dimensionality of 1600. We additionally replicate our findings in Figure 5 with three other models: *RoBERTa-Large*, *Llama-3.2-3B-Instruct*, and *RWKV-4-3B-Pile*.

Line 439: "L2 penalty [Dupré la Tour et al., 2022] ??." —missing citation?

We have corrected this citation in the updated draft.

Line 451: "ane expanded" > "an expanded"

We have corrected this in the updated draft.

Line 630: "We note that squared error values from a model are correlated, which means that the t-test is biased towards positive results which show that an LLM contributes significant variance over a set of simpler models." I didn't follow this sentence. You mean squared error values will be correlated across cross-validation folds? Or squared error values will be correlated with model complexity?

Sorry for the confusion. We meant that, across consecutive samples (i.e. timepoints), squared error values are not independent. This is because both the neural responses as well as the model predictions each exhibit some degree of temporal autocorrelation. However, t-tests assume that samples are independent, an assumption that is violated by us using the squared error values at each time point as individual observations. The point we were trying to get across is that, although this is a violation of the t-test's assumption, it is one that would have the effect of producing *false positives*, not false negatives, as it would effectively overstate the sample size (i.e. number of independent timepoints). Because our goal in this work was to minimize the number of voxels/electrodes/fROIs for which an LLM contributes significant variance beyond the simple models, this bias towards false positives should only work against us (i.e. it should only increase the number of voxels/electrodes/fROIs in which we report that LLMs explain significant additional variance beyond simple features). However, we have also replicated all our analyses using a blocked paired t-test which mitigates this concern. We describe the blocked paired t-test in the response to an earlier comment above where the reviewer mentioned using a blocked permutation test.

Changes to Methods

While squared error values are not always normally distributed, our sample sizes were large: (the minimum sample size was 243), so we still opted to use a t-test over a non-parametric alternative Lumley et al. [2002]. We note that squared error values from a model are correlated across consecutive samples, which means that the t-test is biased towards positive results which show that an LLM contributes significant variance over a set of simpler models i.e., the t-test has an inflated Type 1 error). While this bias is not ideal, it is conservative in our case, making it harder to account for the neural variance that an LLM explains with simpler models.

Line 745: Hawinkel reference is missing the date.

We have updated the manuscript to include this reference.

I include many references below because I think it's critical for the authors to be comprehensive about who's done what in a piece like this.

We have included the majority of these references into Supplementary Table 1 which provides a more comprehensive overview of the field.
